# Assessing the Health of the U.S. West Coast with a Regional-Scale Application of the Ocean Health Index

**DOI:** 10.1371/journal.pone.0098995

**Published:** 2014-06-18

**Authors:** Benjamin S. Halpern, Catherine Longo, Courtney Scarborough, Darren Hardy, Benjamin D. Best, Scott C. Doney, Steven K. Katona, Karen L. McLeod, Andrew A. Rosenberg, Jameal F. Samhouri

**Affiliations:** 1 National Center for Ecological Analysis and Synthesis, Santa Barbara, California, United States of America; 2 Bren School of Environmental Science and Management, University of California, Santa Barbara, California, United States of America; 3 Imperial College London, Silwood Park Campus, Ascot, United Kingdom; 4 Marine Chemistry & Geochemistry Department, Woods Hole Oceanographic Institution, Woods Hole, Massachusetts, United States of America; 5 Conservation International, Arlington, Virginia, United States of America; 6 COMPASS, Oregon State University, Department of Zoology, Corvallis, Oregon, United States of America; 7 Union of Concerned Scientists, Cambridge, Massachusetts, United States of America; 8 Conservation Biology Division, Northwest Fisheries Science Center, National Marine Fisheries Service, National Oceanic and Atmospheric Administration, Seattle, Washington United States of America; 9 Digital Library Systems & Services, Stanford University, Stanford, California, United States of America; Northwest Fisheries Science Center, NOAA Fisheries, United States of America

## Abstract

Management of marine ecosystems increasingly demands comprehensive and quantitative assessments of ocean health, but lacks a tool to do so. We applied the recently developed Ocean Health Index to assess ocean health in the relatively data-rich US west coast region. The overall region scored 71 out of 100, with sub-regions scoring from 65 (Washington) to 74 (Oregon). Highest scoring goals included tourism and recreation (99) and clean waters (87), while the lowest scoring goals were sense of place (48) and artisanal fishing opportunities (57). Surprisingly, even in this well-studied area data limitations precluded robust assessments of past trends in overall ocean health. Nonetheless, retrospective calculation of current status showed that many goals have declined, by up to 20%. In contrast, near-term future scores were on average 6% greater than current status across all goals and sub-regions. Application of hypothetical but realistic management scenarios illustrate how the Index can be used to predict and understand the tradeoffs among goals and consequences for overall ocean health. We illustrate and discuss how this index can be used to vet underlying assumptions and decisions with local stakeholders and decision-makers so that scores reflect regional knowledge, priorities and values. We also highlight the importance of ongoing and future monitoring that will provide robust data relevant to ocean health assessment.

## Introduction

As decision-makers shift towards more comprehensive approaches to managing ecosystems [1]–[3], management goals and targets increasingly focus on overall ecosystem health rather than on single sectors or stressors. This trend is particularly apparent for marine systems where efforts to implement ecosystem-based management (EBM) often have the stated objective of improving ocean health [3]–[6]. Along the United States west coast this emphasis exists in the regional governing body (West Coast Governor's Alliance on Ocean Health; [2]), NOAA's National Marine Sanctuaries' regular assessments of condition [7], ecosystem based approaches to fisheries management plans [8], and state-level and local efforts such as the Marine Life Protection Act, Puget Sound Action Agenda, and the west coast EBM network [9]–[11]. Until recently a standard tool to measure and track changes in ocean health in a repeatable, transparent, quantitative and goal-driven manner was lacking, although it is key to informing management and policy [3], [12]–[13]. Within the United States, both federal and state agencies must make decisions regarding changing ocean uses, new regulations, balancing needs of multiple stakeholders, and supporting coastal economies, along with many other issues. We developed the Ocean Health Index (hereafter, the Index) in part to help address these needs [14].

**Figure 1:**
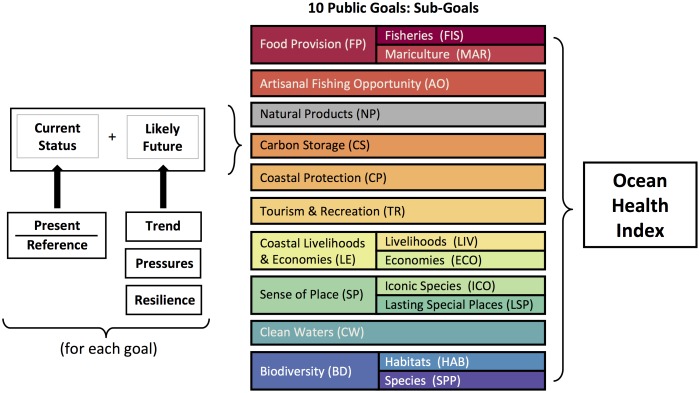
Schematic of the Ocean Health Index showing the 10 goals that comprise it, some with sub-goals, and broadly how each goal is calculated. Natural products is grey to indicate that it was not relevant in the U.S. west coast and thus not assessed.

Public policy necessarily serves multiple interests and goals, such as biodiversity conservation, food production and many others, and thus relies on assessments of ecosystem health through the human lens of meeting societal goals and delivering desired benefits (see File S1). This perspective on ecosystem health is a departure from traditional conservationist views that focus on health as a measure of pristineness (recently debated by [15]–[17]). Assessments of ocean health are thus measured and bounded by human interactions with the ecosystem, not solely by the state of the natural ecosystem. A growing scientific literature also focuses on ecosystems as coupled social-ecological systems (e.g., [18]–[20]). We thus define a healthy ocean as one that sustainably delivers a range of benefits to people now and in the future (Fig. 1; [14]).

As a consequence of this human focus, individuals may perceive and understand ecosystems differently and have different views and sets of values that influence their assessment of ecosystem health. These differences require addressing questions such as: which aspects of ecosystem health are more or less important, how does one set reference points used to quantify health (e.g., ambitious versus practical), and which proxy measures can and cannot be used to estimate the status of each dimension of ecosystem health. Answering these kinds of questions exemplifies the types of inherently subjective decisions that must be made when developing an indicator of ecosystem health. The role of subjectivity cannot be ignored or avoided, but instead it should be made fully transparent, allowing subjective decisions to be modified to suit specific applications in a way that reflects regional values. The Ocean Health Index was designed in part to address this challenge with respect to measuring the health of marine ecosystems.

The Index has many potential applications. One, comparison of performance among regions, requires data consistency across regions. A second, comparison of ocean health within a region over time, makes using the best regionally (as opposed to globally) available data paramount. Halpern et al. [14] exemplifies the first type of assessment. Here we demonstrate an application of both types of assessment, exploring how well the Index performs at a regional scale in a relatively data-rich setting and how sub-regional comparisons might inform local and regional management decisions along the west coast of the United States.

We addressed three core questions: 1) How can the Index framework be adapted to a regional setting and make the best use of locally-available information, i.e., calculating components based on new and different data? 2) What is the health of the U.S. west coast and how is it changing? 3) How can the Index and its underlying framework tie into regional policy decisions? In addressing these questions we also hope to provide guidance and insight to other scientists and practitioners who may want to apply the Index to a new region.

## Methods

### Case study region

**Figure 2:**
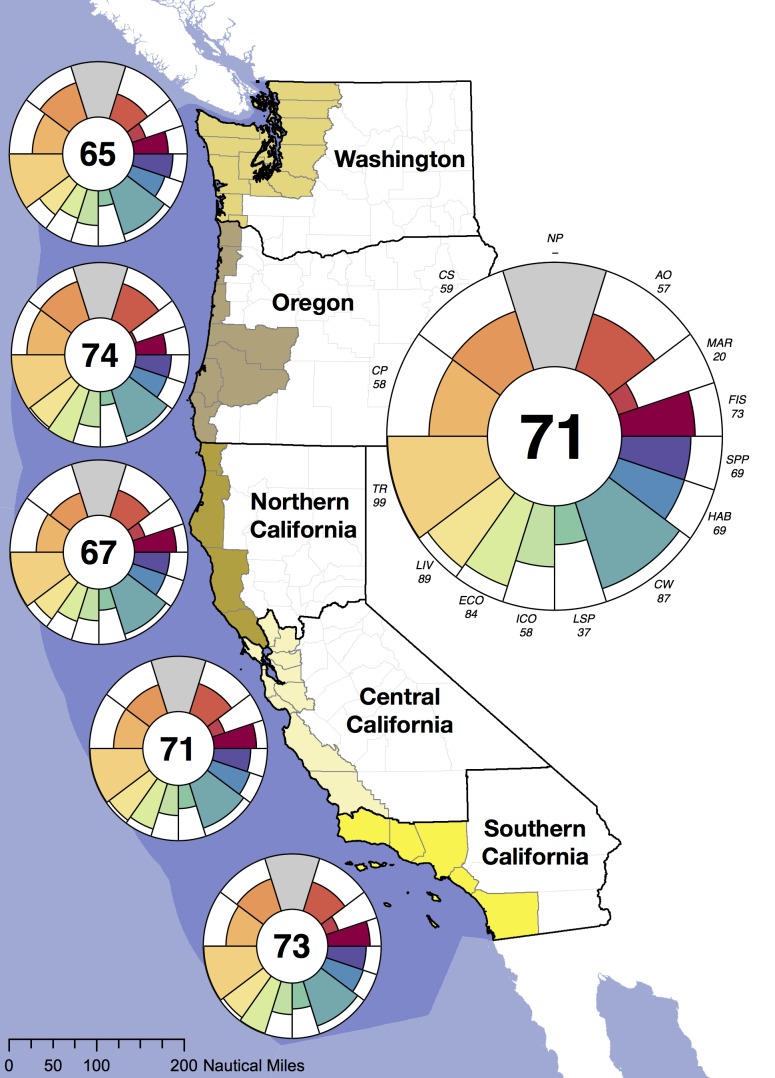
Map of the study region with each goal score per sub-region (left) and for the overall U.S. west coast (right). Each petal in the plots represents the score (radius) and weight (width) for the goal or sub-goal; see Fig. 1 for color legend and goal names. The number in the center is the overall Index score. Natural products is not assessed. Regions are depicted with coastal counties and the 200 nm exclusive economic zone is shaded in darker blue for reference only; regional scores are the area-weighted average of sub-region scores.

We calculated Index scores for five coastal sub-regions of the U.S. west coast –Washington, Oregon, Northern California, Central California, and Southern California – as well as an area-weighted average of these sub-regions to produce an overall regional score. These sub-regions were chosen based on a compromise between political and ecological boundaries. Our focus here is primarily on political boundaries, as most data are gathered and reported by agencies and organizations based on jurisdictional boundaries. However, because California's large geographical extent and ecological, social and economic diversity justified further subdivision, we used county boundaries, which closely align with biogeographic boundaries (Fig. 2), to define three sub-region boundaries. We did this because many data are reported at the county level, which facilitated calculating the Index scores within these sub-regions. The Index could be applied to ecological regions (such as marine ecoregions; [21]), but insufficient data are currently reported at these scales to make this feasible.

From a socio-economic perspective, the five sub-regions differ substantially (e.g., [22]). Southern California contains the heavily urbanized and densely populated coasts of San Diego and Los Angeles but also includes eight large, nearly uninhabited offshore islands. Central California is more sparsely populated but also includes the densely-populated San Francisco Bay. Northern California is even less densely populated, except in Sonoma county bordering San Francisco Bay. The coast of Oregon is uniformly sparsely populated, with just 16,000 inhabitants in the largest town (Coos Bay). Washington state includes two distinct regions – the outer coast, which is similar to Oregon's coast, and Puget Sound, which includes many urbanized areas, most notably Seattle and Tacoma.

### Index calculations

The Index is comprised of ten widely-held goals for healthy oceans that capture the different aspects of how people use, benefit from and value ocean ecosystems: food provision, artisanal fishing opportunities, natural products, carbon storage, coastal protection, sense of place, tourism & recreation, coastal livelihoods & economies, clean waters, and biodiversity (see Table 1 for goal definitions). These goals broadly map to the set of ecosystem services described by others (e.g., [23]), but have important differences (such as inclusion of coastal livelihoods and economies, which is not an ecosystem service) that motivate calling them public goals rather than ecosystem services.

**Table 1:** Details for the current status calculation of goals and sub-goals that comprise the Ocean Health Index.

Goal	Sub-goal	Definition	Reference point type	Reference point	Data used
**Food provision**	**Fisheries**	Harvest of sustainably caught wild seafood	Functional Relationship	Single species biomass at maximum sustainable yield (BMSY) and single species fishing mortality at maximum sustainable yield (FMSY)	B/BMSY and F/FMSY estimates from stock assessments; mean annual commercial catch per species
	**Mariculture**	Production of sustainably cultured seafood	Established Target	350% increase in production from 2005 levels, distributed evenly among farmable areas in all sub-regions	Tons of shellfish produced; areas deemed safe for mariculture farming by NOAA
**Artisanal fishing opportunity**		Opportunity to engage in artisanal-scale fishing for subsistence or and/or recreation	Established Target	**Physical Access:** One coastal access points per mile of coastline	**Physical Access:** Number of coastal access points per mile
				**Economic Access:** No increase in the ratio of fuel price to median income over a five-year period	**Economic Access:** Change in gas price over time
				**Resource Access:** Perfect sustainability score for all fish stocks	**Resource Access:** Condition of fish stocks as measured by the NOAA fish Stock Sustainability Index (FSSI)
**Natural products**		Sustainable harvest of natural products, such as shells, algae, and fish oil used for reasons other than food provision	N/A	N/A	N/A
**Carbon storage**		Conservation status of natural habitats affording long-lasting carbon storage	Temporal Comparison (historical benchmark)	**Salt Marshes:** 50% of historical areal extent (roughly since 1850s)	**Salt Marshes:** Areal extent in 2006, 2002, 1996, and the 1850s
				**Seagrasses:** Zero pressure to coastal areas from nutrient input	**Seagrasses:** Nutrient input model applied within the 100 m depth contour
**Coastal protection**		Conservation status of natural habitats affording protection of the coast from inundation and erosion	Temporal Comparison (historical benchmark)	**Salt Marshes:** 50% of historical areal extent (roughly since 1850s)	**Salt Marshes:** Areal extent in 2006, 2002, 1996, and the 1850s
				**Sand Dunes:** 100% of the areal extent in 1960	**Sand Dunes:** Areal extent in 2006, 2002, 1996, and 1960
				**Seagrasses:** Zero pressure to coastal areas from nutrient input	**Seagrasses:** Nutrient input model applied within the 100 m depth contour
**Tourism & recreation**		Opportunity to enjoy coastal areas for recreation and tourism	Temporal Comparison (moving target)	No net loss in participation in marine-related activities over a 10 year period	Model of per capita participation rates in 19 marine-related activities based on demographic variables
**Coastal livelihoods & economies**	**Coastal livelihoods**	Jobs and wages from marine-related sectors	Temporal Comparison (historical benchmark)+Spatial Comparison	**Jobs:** No net loss in the number of jobs in marine-related sectors relative to all job sectors in each region over a five-year period	Jobs and wages data for 20 marine-related sectors; total jobs (marine and non-marine sectors)
				**Wages**: Highest per capita average annual wages across all regions and marine sectors	
	**Coastal economies**	Revenues from marine-related sectors	Temporal Comparison (moving target)	No net loss in revenue in marine-related sectors relative to all economy sectors over a five-year period	Revenue data for 20 marine-related sectors; total revenue (marine and non-marine sectors)
**Sense of place**	**Iconic species**	Cultural, spiritual, or aesthetic connection to the environment afforded by iconic species	Established Target	All assessed species coservation status classified as of least concern	Species conservation status as determined by NatureServe criteria
	**Lasting special places**	Cultural, spiritual, or aesthetic connection to the environment afforded by coastal and marine places of significance	Established Target	30% of all marine and terrestrial areas protected	Marine and terrestrial areas protected and managed for conservation
**Clean waters**		Clean waters that are free of nutrient and chemical pollution, marine debris and pathogens	Established Target	Zero marine debris, nutrient run-off, beach closures due to pathogens, and chemical contaminants in sediments and bivalve tissue	Nutrient plume models; beach closure data; beach clean-up data; concentration of chemicals in sediment and bivalve tissue samples
**Biodiversity**	**Habitats**	The existence value of biodiversity measured through the conservation status of habitats	Temporal Comparison (historical benchmark)	**Salt Marshes:** 50% of historical areal extent (roughly since 1850s)	**Salt Marshes:** Areal extent in 2006, 2002, 1996, and the 1850s
				**Sand Dunes:** 100% of the areal extent in 1960	**Sand Dunes:** Areal extent in 2006, 2002, 1996, and 1960
				**Seagrasses:** Zero pressure to coastal areas from nutrient input	**Seagrasses:** Nutrient input model applied within the 100 m depth contour
				**Soft-bottom**: Zero pressure from bottom trawl fishing	**Soft-bottom**: Amount of fish caught using bottom-trawl methods and location of current soft-bottom habitats
	**Species**	The existence value of biodiversity measured through the conservation status of marine-associated species	Established Target	All assessed species extinction risk status classified as of least concern	Species extinction risk status as determined by IUCN criteria

An overall Index score, *I*, is calculated as the weighted sum of the scores for each goal assessed in the Index ([14] and Fig. 1), such that:
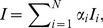
(1)where α is the importance (i.e. weight) placed on each goal *i*. For the U.S. west coast analysis we assumed equal weights for all *N* goals. In a previous application of the Index at the global scale, when a goal was not relevant to a location (for example, extractive goals for uninhabited islands or assessments of coral health in countries that do not have coral reefs), it was dropped from the assessment as it was deemed not applicable. In other words, in these cases the community assigns no value to the goal (i.e., the goal weight is zero; [14]), thus resulting in its irrelevance to the overall assessment. In this U.S. west coast application, we exclude the natural products goal because for most products there is no recorded trade within the region, even though it likely occurs at small scales, and for kelp, limited commercial harvest existed in Southern and Central California but no longer occurs (for unknown reasons). We further explain our rationale for excluding this goal in File S1.

Goal scores were calculated as the average of current (*x_i_*) and likely future status (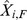) Current status was measured as the present value (*X_i_*) relative to a reference value (*X_i,R_*), such that *x_i_* = *X_i_*/*X_i,R_*. Likely future status was measured as current status modified by the recent trend (*T*), cumulative pressures (*p*), and resilience (*r*), such that:
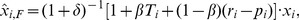
(2)where δ is the discount rate (δ = 0) and β is the relative importance of trend versus the difference between pressures and resilience in determining the likely future status (we assumed β = 0.67, following Halpern et al. [14]). Note that the likely future status does not predict the future, but only estimates what the status score is likely to be approximately 5 yr hence, given what is known today about recent trends and the counterbalance of pressure versus resilience metrics. Reference points for each goal are described in Table 1 (and further detailed in File S1). These reference points are similar but not equivalent to management targets; a reference point as we define it here is the maximum sustainable level of production of each goal. In some cases management may choose a target different than these reference points for practical or sociopolitical reasons [24]. Resilience measures focused on the presence of relevant institutions, but in general could not evaluate their effectiveness due to lack of such data. Details of goal models and parameters are provided in Table 2.

**Table 2:** Models and parameter used to calculate each goal and sub-goal.

Goal	Sub-Goal	Status Model Equations	Variables
Food Provision (*x_FP_*)		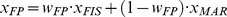	
		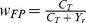	*Y_r_* = Total sustainable mariculture harvest; *C_T_* = Total current wild-caught fishing yield; *w_FP_* = weight per seafood sector
		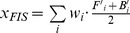	*w_i_* = weight per stock *i*; *F* = current fishing mortality of stock *i; B* = current biomass of stock *i*
		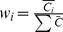	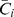 = mean catch of stock *i* throughout the time-series
	Fisheries (*x_FIS_*)	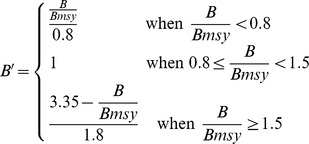	*B_msy_* = biomass of stock *i* producing maximum sustainable yield
		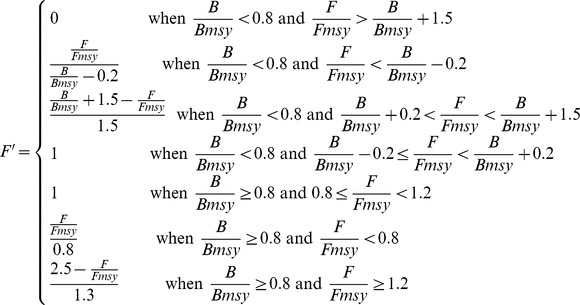	*F_msy_* = Fishing mortality that yields long term maximum sustainable yield of stock *i*
	Mariculture (*x_MAR_*)	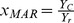	*Y_C_* = Current total sustainable mariculture harvest
		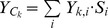	*S* = Sustainability coefficient for species *i; k* = sub-region
		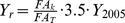	*FA* = farmable area; *Y_2005_* = yield in 2005
Artisanal Fishing Opportunities (*x_AO_*)		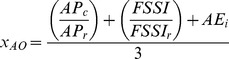	*AP* = average distance between coastal access points; *c* = current; *r* = reference; *FSSI* = catch-weighted average NOAA Fish Stock Sustainability Index score
		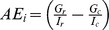	*AE* = economic access; *G* = gas price per gallon; *I* = median income
Natural Products (*x_NP_*)		Not assessed for this region	
Carbon Storage (*x_CS_*)		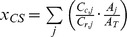	*A_k_* = area covered by habitat *j; A_T_* = total area covered by all habitats; *C_j_* = condition of habitat *j*
Coastal Protection (*x_CP_*)		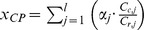	*r_j_* = protective ability rank of habitat *j*
		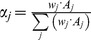	
		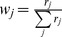	
Coastal Livelihoods & Economies (*x_LE_*)		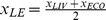	
	Livelihoods (*x_LIV_*)	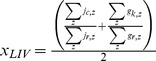	*j* = adjusted number of direct and indirect jobs within sector *z; g* = average PcPPP-adjusted wages within sector *z; c* = current year; *r* = reference year (*j, e*) or reference location (for *g*)
	Economies (*x_ECO_*)	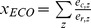	*e* = total adjusted revenue generated directly and indirectly from sector z
Tourism and Recreation (*x_TR_*)		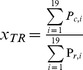	*P_c_* = current predicted participation in each recreation activity *i* (of 19); *P_r_* = observed participation in recreation activity i in year 2000
Sense of Place (*x_SP_*)		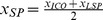	
	Iconic Species (*x_ICO_*)	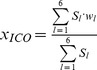	*l* = IUCN threat category; *S_m_* = number of assessed iconic species in category *l; w_l_* = weight per threat category l
	Lasting Special Places (*x_LSP_*)	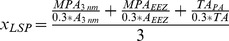	*MPA* = fully protected marine area; *_EEZ_* = offshore waters (3–200 nm); *3 nm* = coastal waters (0–3 nm); *TA* = area on coastal land (0–1 mi); *TA_PA_* = protected area on coastal land
Clean Waters (*x_CW_*)		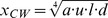	*a* = population without access to sanitation relative to global maximum; *u* = 1 – nutrient inputs; *l* = 1 – chemical inputs; *d* = 1 – marine debris
Biodiversity (*x_BD_*)		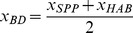	
	Species (*x_SPP_*)	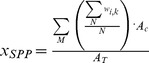	*n* = number species per grid cell *c; m* = number of grid cells in the assessment region; *A_c_* = total area of grid cell *c; A_T_* = total area of the assessment region
	Habitats (*x_HAB_*)	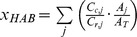	See variables above

See File S1 for details on data and rationales for each.

### Changes to Index calculations for regional application

As compared with the global application of the Index [14], higher resolution data and longer time series, along with a better understanding of the regional context, allowed for improved approaches to modeling and setting reference points for many goals. Briefly, the following changes were made (see also File S1):

1. **Higher resolution data**. We used regional-scale data wherever possible, relying on national data used in Halpern et al. [14] in only 20% (10 of 49) of data layers. In particular, we were able to use higher resolution data from local sources for the status and trend calculation of all goals. Most of the pressure layers, and all of the resilience layers, were also calculated using local data sets.
2. **Models adapted to better represent regional goals for ocean health**. For several goals we modified the approach to assessing current status based on higher-quality regional data. (a) For food provision derived from wild-caught fisheries, we were able to use formal stock assessments routinely employed for local fisheries management to capture the status of major commercially-caught species. These estimates are derived from complex models developed by working groups of experienced local experts. In contrast, the global analysis had to rely on models requiring many simplifying assumptions, leading to higher uncertainty. (b) For food provision derived from mariculture, we improved our estimate of potential sustainable productivity by assuming cultivation could only increase in areas already under production. In contrast, the global analysis assumed potential productivity scaled to total coastal area and highest observed production density, i.e. China. (c) For the tourism & recreation goal, we were able to use information on participation rates in a range of coastal and marine tourism and recreational activities. Participation rates more closely match the intent of this goal and are a more robust proxy than the international tourist arrivals data used in the 2012 global study [14] and are a more direct measure than the tourism employment proxy used in the 2013 global study [25]. We also changed the reference point from spatial (used in the global analysis) to temporal, because adequate time-series data were available.
3. **Reference points based on U.S. west coast priorities.** (a) For the mariculture sub-goal, the reference point was based on regional projections of nationally-desired economic and food security targets. (b) For the habitat health scores used in coastal protection, carbon storage, and the habitats sub-goal of biodiversity, we used reconstructions of historic extents, rather than recently recorded trends, to set targets that were more ambitious than in the global analysis. (c) For the lasting special places sub-goal, in addition to evaluating protected areas inland and within 3 nm of the coast, we included a third zone (3 to 200 nm offshore) because we assume assessment of places offshore as well as nearshore is important to people in this region.

We conducted a number of analyses to assess how results were affected by various assumptions and data constraints (File S1), three of which we focus on here. First, to assess how results changed when goals were modeled differently, we calculated the regionally-modified goals using methods from the global study for comparison, when possible. Second, we assessed how alternate reference points modified goal scores, in particular for the mariculture sub-goal (see File S1). Finally, we assessed the consequences of using empirically-derived unequal weights, elicited from regional experts representing a diverse cross-section of stakeholders [26], for combining goals into a single Index score (Table S11 in File S1). Experts weighted sense of place and clean waters goals highest, and three to four times more heavily than the tourism & recreation and coastal livelihoods & economies goals, the two lowest-weighted goals (Table S11 in File S1).

Although we were able to estimate past status values for all goals and sub-goals except tourism and recreation, iconic species, and species diversity (which had insufficient time series; see File S1), most pressure and resilience metrics were not available for past time periods, precluding calculation of the ‘likely future status’, and thus the overall Index scores, for past years.

### Scenario analyses

To further explore how the Index could be used within typical regional-scale decision contexts and to illustrate how the Index responds to typical management actions, we simulated several scenarios and recalculated the overall Index. The intent of this analysis was not to model precise changes but rather to illustrate expected types and relative magnitudes of change across goals. Rather than being prescriptive, these scenarios were chosen to illustrate how one can use the Index to explore consequences of management decisions. We recognize that realistic implementation would require engagement with decision-makers, normative decisions about management goals, fine-tuning of assumptions, and model-based simulations of future conditions. Our heuristic scenarios assessed what scores would be if 1) regulations had been adopted 5 years ago that successfully reduced land-based runoff of nutrients and pollutants each by 25%, 2) habitat restoration activities had been successfully implemented such that coastal wetlands and sand dunes were increased in extent by 10%, and 3) the Marine Life Protection Act (MLPA) process in California had not occurred and the currently-existing network of MPAs therefore had not been established within the state. For this third scenario we implemented three successive versions intended to measure changes over time in how the system, and thus the Index, would respond (for details see File S1).

## Results

Overall the U.S. west coast scored 71 out of 100. Washington scored lowest of all sub-regions (65), with increasingly higher scores in Northern California (67), Central California (71), Southern California (73), and Oregon (74; Fig. 2). Goal scores varied from 22 (mariculture) to 99 (tourism and recreation), with tourism and recreation and clean waters scoring highest, and carbon storage, coastal protection, lasting special places and mariculture scoring lowest for all 5 sub-regions (see also Table S33 in File S1). Despite biophysical and socioeconomic differences among sub-regions, overall Index scores for the sub-regions were within a 7 point range. Differences in scale, available data, and goal methodologies for several goals preclude direct quantitative comparison of these results to global scores [14].

**Figure 3:**
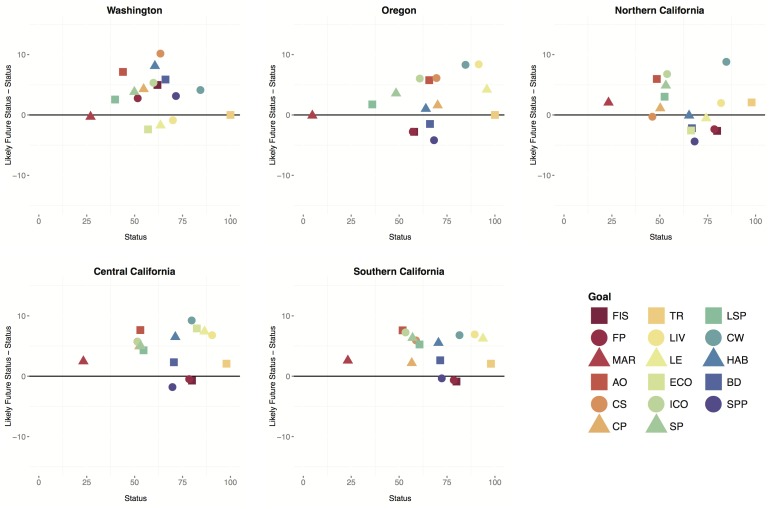
Current status versus the difference between likely future and current status for each goal and sub-goal within each sub-region. Values above the y-axis indicate the likely future status is greater than the current status. Note that y-axis is scaled −10 to 10.

**Figure 4:**
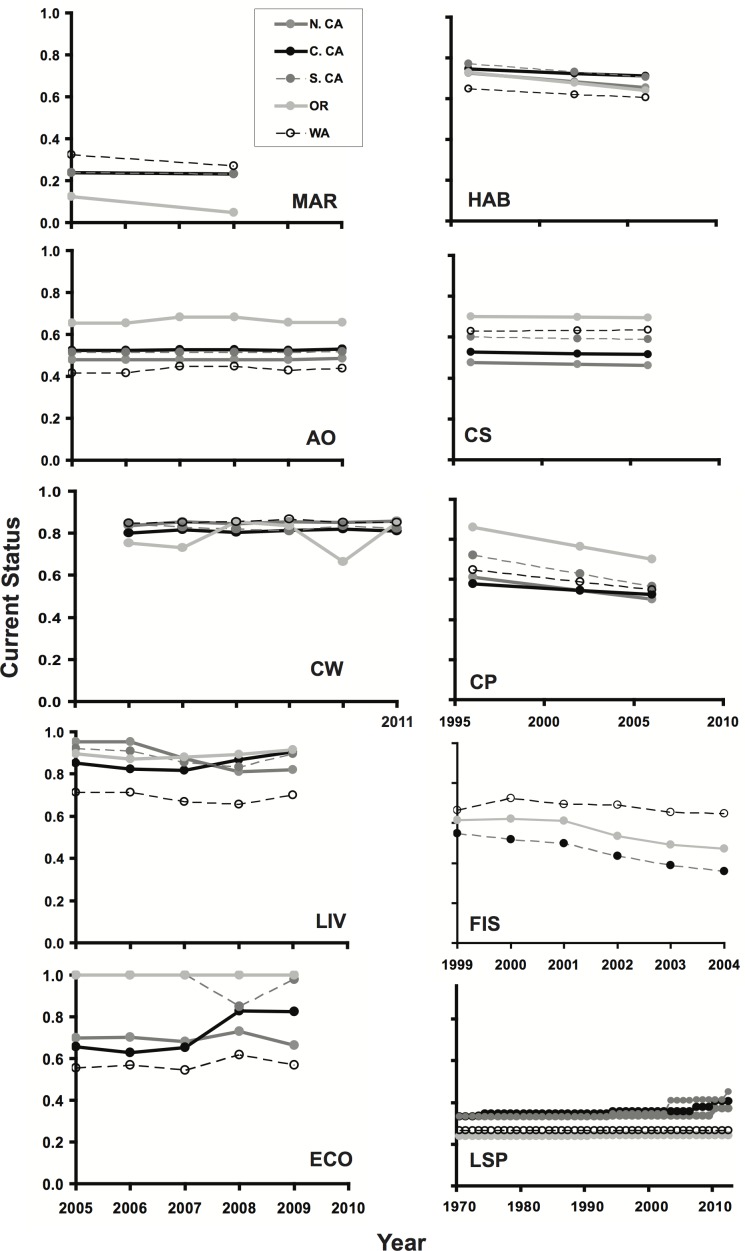
Time series of current status scores for goals and sub-goals with available historical data. Note different time scales on x-axes in right-hand plots. Plots are for the habitat sub-goal (HAB), carbon storage (CS), coastal protection (CP), artisanal fishing opportunities (AO), mariculture sub-goal (MAR), clean waters (CW), fisheries sub-goal (FIS), coastal livelihoods sub-goal (LIV), coastal economies sub-goal (ECO), and the lasting special places sub-goal (LSP). FIS could not be assessed for sub-regions within California and so a single state-level result is presented in that case.

For nearly every goal in each of the five sub-regions, likely near-term future scores were greater than those for the present (Fig. 3). Although the likely future state is strongly influenced by the recent trend (see Eq. 2), and the recent trend for many goals was (slightly) negative, the likely future status is also influenced by the balance between resilience measures and cumulative pressures, and in many cases resilience was greater than pressures (Table S34 in File S1). In only 24% of cases (11 of 45) were likely future scores worse, namely coastal livelihoods & economies in Washington; fisheries and species biodiversity in Oregon; fisheries, species biodiversity, carbon storage, and coastal livelihoods & economies in Northern California; and species biodiversity and fisheries in Central and Southern California. The potential future declines in the health of the fisheries (food provision) and species (biodiversity) sub-goals, despite significant resources being committed to their improvement in the region, is largely due to recent declines creating a negative trend (see below, Fig. 4, and Table S34 in File S1).

The likely future status does not tell the whole story, however. For goals and sub-goals with sufficient data to calculate past values of the current status, different patterns emerged. Habitat-based goals, notably habitat diversity and coastal protection, showed declines (8–17% respectively) across all sub-regions over the past ten years (Fig. 4). Coastal livelihoods and economies showed initial small declines in some sub-regions but recent recovery in many cases (Fig. 4). Because these values are standardized to remove broader economic patterns, this result suggests stronger effects of the global recession that began in 2008 and slower economic recovery in marine sectors compared to other sectors. Lasting special places showed recent improvements, in large part because of California's MPA initiatives, while remaining goals showed little recent change (Fig. 4). Because risk status of most species is rarely assessed more than once, we were not able to calculate past status scores for species diversity or iconic species sub-goals.

Comparisons of results obtained for several goals when assessed with the previous global approaches versus the refined regional approaches showed important differences. For the tourism and recreation goal, scores from the regional analysis were considerably higher than those obtained applying the global model, most likely reflecting both use of more informative data on participation rates instead of international tourist arrival data and the choice of a local temporal reference point instead of an across-region spatial one (Table 3). For large countries with coasts spanning sizable biophysical gradients or bordering different oceans, such as the United States, sub-national assessments such as the one here are likely to produce Index scores that differ from those derived from the national-level global assessment. In contrast, the choice of a very different approach to modeling the artisanal fishing opportunity goal had relatively small effects on resulting scores (Table 3). Changes in the mariculture reference point significantly increased scores relative to the global approach, with highly variable results using other methods for setting reference points (Table 3). Unequal goal weighting [26], which represents one example of how people value goals differently, produced lower Index scores for some sub-regions and higher scores for others (Table 4).

**Table 3:** Changes in goal scores for which sensitivity analyses were conducted using alternate methods for calculating goal status.

	Fisheries		Mariculture					Tourism and Recreation		Artisanal Fishing Opportunity	
Region	Orig without data poor stocks	Alt with data poor stocks	Orig federal target	Alt1 spatial ref; global target	Alt2 spatial ref; national target	Alt3 temporal ref	Alt4 production function	Orig temporal ref	Alt spatial ref	Orig regional model	Alt global model
Washington	64	55	27	1	81	80	10	100	55	47	61
Oregon	56	54	5	0.1	6	0	1	100	41	69	61
California	79	65		0.1	9	96	19	99	91		
N. California			24							51	59
C. California			24							57	58
S. California			25							55	59

Results are reported for each sub-region separately when possible. ‘Orig’ is the original approach used for reporting main results; ‘Alt’ is the alternative approach used for sensitivity analyses. Separate analyses for each sub-region within California were only possible for the artisanal fishing opportunity goal and for the original mariculture goal; results for other cases are reported for all of California as a single value. For mariculture we tested four different alternate reference points. See File S1 for details.

**Table 4:** Changes in Index scores for each subregion and the U.S. West coast with goals weighted equally or unequally based on regionally-specific, empirically-derived preferences (Halpern et al. 2013b).

Region	Equal	Unequal
Washington	65	66
Oregon	74	74
N. California	67	66
C. California	71	69
S. California	73	71
U.S. west coast	71	70

**Figure 5:**
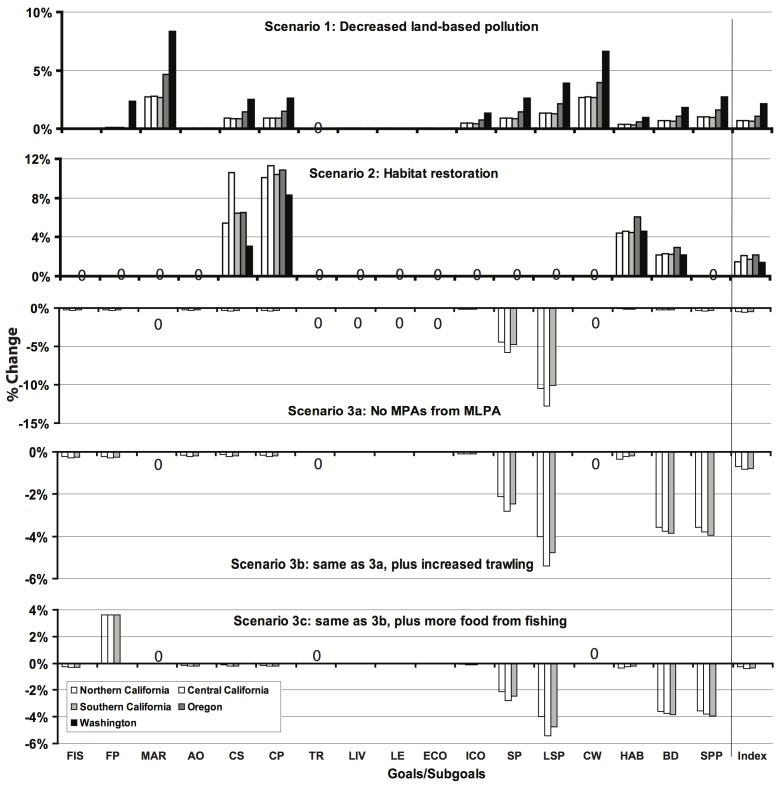
Scenario results as percent change in goal and Index scores for each sub-region. Goals with no change are indicated with a zero. Overall Index scores are on the far right, separated by the horizontal gray line. Note different scales on y-axes.

The three management scenarios showed goal score changes from about −12% up to +11% depending on the goal and scenario (Fig. 5). For example, because land-based pollution is a pressure on nearly every goal, hypothetical decreases in this stressor led to modest increases in most goals (scenario 1). Simulated habitat restoration had a relatively large effect on habitat-based goals, a result that was influenced by choice of habitat reference points (see File S1, scenario analysis section), but not on other goals (scenario 2). The three versions of scenario 3 illustrate how an initial action (or in this case hypothetical lack thereof) could have cascading effects across multiple goals that may lead to increases in some goals and decreases in others. In the example here, the hypothetical removal of MPAs decreased the lasting special places score, increased the food provision score (through increased fishing), and decreased scores for other goals as a result of increased fishing pressure.

## Discussion

By calculating the Index for the U.S. West coast, we were able to take advantage of regional data and knowledge of the system to assess how particular goals and overall ocean health are faring at a regional scale, and whether conditions are getting better or worse. We found that current status scores for individual goals have gotten worse in the past decade or so (with the exception of recent improvements in lasting special places an livelihoods and economies for some sub-regions), but in most cases the near future looks better than the present (Figs. 3, 4). Assessment of the likely future status rewards the presence of regulatory and management measures; however, as data do not often exist on effectiveness of these measures, future estimates may be overly optimistic. In addition, the likely future status makes incorporates the potential impacts of climate change only as current climate-related pressures and not estimates of future conditions. Regardless, these differences highlight the importance of having time-series and maintaining on-going records of both ecological and governance information in order to understand likely future changes. The Index was designed explicitly to capture and quantify these different temporal components.

Spatial comparisons of sub-regional scores offered additional insights. Sub-regional scores had relatively small differences compared to the large range of scores globally in Halpern et al. [14]. This is understandable because biophysical and socioeconomic characteristics within the study region vary much less than among countries across the globe. Regional scores from this assessment were all higher than the score for the entire U.S. calculated from the global assessment. We cannot distinguish whether this difference stems from the use of different methods and data sources or if it suggests support for the widely-held view that the U.S. west coast is a relatively healthy and sustainably managed system. The overall U.S. score derived in the global study integrates scores from diverse coastal regions (i.e., Alaska, Hawaii, Gulf of Mexico, and east and west coasts of the U.S.) that vary historically, ecologically and in resource management actions.

Individual goal scores showed bigger sub-regional differences, but these differences were dampened when averaged with other goal scores to create overall Index scores (assuming equal weights for all goals; see File S1). For example, the fisheries sub-goal scored considerably higher in California (79; the Californian sub-regions could not be assessed separately for this goal due to the resolution of reported data) than in Oregon (56) and Washington (64; Fig. 2, Table S33 in File S1). This difference is in part due to differences in the dominant stock in the different regions; hake historically constituted roughly 30% of total catch in Oregon and Washington and are currently at low biomass and fishing effort levels (giving a score of 0.39 for the stock), whereas in California yellowfin tuna historically represented roughly 40% of total catch and are currently at ideal biomass and fishing effort levels (a score of 1.0).

The coastal livelihoods and economies sub-goals had very high scores for Central and Southern California and Oregon yet much lower scores for Northern California and Washington (Fig. 2, Table S33 in File S1). This goal uses a ‘moving window’ reference point, comparing each region to itself five years prior (while correcting for broader economic patterns, such as the global recession, that are independent of the condition of marine and coastal systems), based on the assumption that people mostly care about how they are doing economically relative to recent and local economic conditions. This technique avoids direct comparison, for example, of the absolute size of Southern California's coastal economy to that of Oregon. Consequently, Washington and Northern California scored lower because the largest sectors had significant declines in the last five years (in Washington, large declines occurred for jobs in tourism and transportation sectors and for revenue in tourism and living resources sectors; in Northern California, declines occurred for jobs and revenue in tourism), while such declines generally did not occur in the other regions.

Variation among sub-regions for the carbon storage goal (as well as the generally low scores for this goal for all sub-regions) is primarily due to the status of salt marsh habitats. Although salt marsh habitat loss has occurred throughout the U.S. west coast, this has been particularly severe in Central and Northern California. The exact values of the scores for this goal are highly dependent on the choice of reference point, which in this case is challenged by both practical and philosophical issues. Practically, few spatial data exist prior to the 1990s. Therefore, to set an ambitious yet realistic reference point, we relied on estimates of historical loss of these habitats from pre-industrial times and set the reference point to a fraction (50%) of this original extent. Philosophically, one must (subjectively) decide what serves as an ambitious yet realistic target (cf. [24]) for restoring this habitat, given that a great deal of potential ecosystems services were lost but also considering that the massive alteration driven by urbanization of estuarine systems is unlikely to be completely reversed.

Finally, differences among sub-region scores for the artisanal fishing opportunity goal were driven primarily by differences in coastal access, which together with economic factors and fish stock status determined this goal's score. Public access is provided along Oregon's entire coastline, leading to a higher score, whereas Washington and California both allow privately-owned access to the coast and have large stretches of restricted-access coastline. The choice of a common reference point for all sub-regions allows for direct comparison, but ultimately may not reflect sub-regional differences in management objectives, such as how local people want artisanal fishing to occur. If such differences are significant enough and the objective of local managers is primarily on managing at this finer scale, then a separate Index score, calculated with sub-regional best available data and locally-determined reference points, would be more appropriate.

Other goals are consistently low or high across all sub-regions but with results that may not immediately seem intuitive. For example, all sub-regions would like to increase coastal tourism, yet all scored nearly perfectly on this goal. One might hope that a different type of reference point could resolve this paradox. A functional relationship between people's values and the effects on the ecosystem caused by different levels of participation in coastal recreation would be ideal, as it could indicate what absolute levels of coastal tourism are both wanted by local communities and sustainable for local ecosystems. Unfortunately we do not have the information to construct one and it could differ in the different sub-regions. A spatial comparison reference point would probably not be appropriate as coastal communities in Oregon, for example, are very different biogeographically than those in Southern California, with local population density, weather and beach access being some of the place-specific factors affecting the number and frequency of people recreating in and around the ocean. Consequently, spatial comparison using a region-wide reference point would unfairly penalize one of the locations and would not be a useful indicator of ocean health. Currently no stated objectives for desired levels of tourism exist that could be used for an ‘established target’ reference point. This leaves temporal reference points as the best choice in this specific case, and, for reasons similar to economically-based temporal reference points for the livelihoods & economies goal, we used a moving-window reference point (i.e., that conditions are as good or better than they were 5 years ago). For all sub-regions, participation in coastal recreational activities has remained the same or increased in the past 5 years.

A similar issue of choice of reference point affected the scores for the mariculture sub-goal. We used an established target (increase mariculture by 350% from 2005 to 2020; [27]), which was based on socio-economic projections of seafood demand. This target produced relatively low scores, but our assessment of a range of other types of reference points showed that these scores are strongly dependent on choice of reference point (Table 3). Ideally we would have used a functional relationship reference point based on biophysical variables and societal preferences for how much available ocean space should be allocated to mariculture versus all the many other uses that currently exist and that will emerge in the near future. Unfortunately we currently have very little of these data, and so we relied on rough estimates of socially and ecologically desirable ‘farmable area’ in each sub-region. Although the estimate from Nash [27] is potentially arbitrary, a 350% increase in production is not unreasonable from an environmental perspective (i.e., very little area is currently dedicated to shellfish farms and the production of shellfish species has a minimal environmental impact), such that the socially/economically desirable reference point reported by federal managers seems reasonable using SMART principles for setting reference points [24]. A production function based on these parameters would likely lead to higher mariculture scores, as the reference points (i.e., targets) would likely be lower. Uncertainty in what these target values should be remains an important gap in our current understanding.

### Lessons learned for regional assessments

Although from a global perspective the U.S. west coast is a relatively data-rich location, data from the recent past were largely lacking, and historical data even more so. Most notably these gaps include habitat condition (current quality, and historical and current extent), conservation status of most species, fisheries stock assessments, historical levels of human pressures on ecosystems, and the nature and effectiveness of regulatory measures – data gaps common nearly everywhere [28]–[29]. The process of pulling together the information necessary to calculate the Index serves as a means to systematically evaluate where key gaps remain. Such gaps are a perpetual challenge for managers and policy makers. Prioritizing efforts to fill those gaps remains critical. The assessment here also highlights the need for new or continued assessment of pressures and resilience measures, not only of status variables, for effective assessment of overall ocean health. Our study offers a valuable starting point, or baseline, for future assessments in this region, but only by filling key data gaps will we gain the ability to determine trends in overall ocean health, a critical need for ecosystem-based management [30]. The sooner that other regions can begin comprehensive and repeatable assessments of ocean health, the better equipped they will be to make fully informed and strategic resource management decisions.

A key challenge for any assessment of ecosystem health is to detect meaningful and significant change in condition. Ideally one can then attribute that change to natural versus anthropogenic drivers of change, although such attribution is notoriously difficult. Along the U.S. west coast efforts to detect and attribute change face the challenge of distinguishing between broad shifts in the ecosystem due to natural climate variability on timescales of interannual (such as El Nino-driven changes) to multi-decadal (such as Pacific Decadal Oscillations; PDO) from longer-term trends driven by human impacts such as climate change. In the Index most goals document the cumulative effect of natural and anthropogenic change but do not explicitly attribute the underlying cause. For example, the biodiversity, carbon storage, coastal protection and clean water goals all have fixed targets that are independent of the cause of change, and the human-focused goals of coastal livelihoods & economies and tourism & recreation are more indirectly affected by such natural variation and are also independent of the cause of change. The fisheries sub-goal of food provision is one case where attribution of change is important, as fisheries management benefits from knowing the cause(s) of mortality. In this case, stock assessment models are usually refined to adjust the reference points and assessments in consideration of known changes in key oceanographic conditions (i.e., adjusting B_MSY_ to account for what is sustainable for a given oceanographic regime). By using information from local stock assessments, the index is adjusted for these effects whenever local information and modeling tools allow.

Importantly, and perhaps unsettling to some, is the reality that assessing something as diverse and comprehensive as ‘ocean health’ requires accommodating regional values and perspectives of the people that are part of the coastal ecosystems being assessed. Within the Ocean Health Index, this means that some conceptual aspects of implementing the Index are inherently subjective. Although the Index framework provides guidelines that can help adapt models to available regional data, using simpler models or different proxies when necessary [14], it cannot prescribe which available regional data sets are preferred. Nor does the framework dictate the most appropriate models to use or choices of reference points, proxy data or goal weights. For example, the way we modified models or reference points used in the global assessment [14] for regional use in the food provision, tourism & recreation, and artisanal fishing opportunities goals (see File S1) has important consequences for the resulting goal scores (Table 3). These adaptations highlight the flexibility of the Index to incorporate different perspectives on how goals should be assessed, as reflected by debates on how fisheries were modeled [31]–[34]. The Index can also accommodate a different set of goals if they better reflect what local communities value, although we posit that the ten goals currently defined within the Index are sufficiently broad to capture a vast majority of values. To some this flexibility may seem to come at the cost of comparability or objectivity, but we argue that any local assessment of ecosystem health faces similar challenges of accounting for local variables and community values. No indicator is exempt from such subjective decisions. The Index's framework, however, requires one to identify, justify and track such assumptions explicitly and thus fosters careful and well-documented assessment of the sensitivity of results to such decisions.

### Policy implications

The Index was explicitly designed to help inform decision-making by providing a comprehensive, comparable, and quantitative assessment of the range of components that drive overall ocean health. As with any decision support tool, the scale of assessment should match the scale of decision-making [35]. Our assessment here is thus most valuable to regional-scale (e.g., West Coast Governors Alliance, [2]; California Current LME, [36] and state-level (e.g., California's Marine Life Protection Act, [37]) decision-making. Decisions at smaller scales (such as Puget Sound or San Francisco Bay) would be best informed by recalculating the Index using best available local-scale information where possible. To help support application of the Index in these (and other) processes in the future, we have developed a software tool (www.ohi-science.org) that allows people to explore Index results as well as recalculate (or calculate anew) scores as new data become available. A key strength, and challenge, of the Index is that it requires an explicit statement of all assumptions and assignment of specific targets for each ocean health goal. The strength lies in providing stakeholders and decision-makers a forum to articulate their reference points and assumptions, while leveraging their values and knowledge, and a means to disentangle and clearly define their multiple, interacting objectives. The challenge arises from the practical (e.g., data constraints) and political (e.g., managing expectations, achieving consensus) process of making these important decisions, and the inherent sensitivity of Index scores to these choices [24]. The Index offers a tool to engage stakeholders and decision-makers in these difficult but necessary discussions, while also helping agencies fulfill their mandates.

For example, the ability to use scenarios to evaluate the likely consequences of any particular management action for overall ocean health provides a powerful decision-support tool, but requires additional assumptions and decisions about how things will likely change in the future. We illustrated such a process with several heuristic scenarios (Fig. 5) intended to show how the Index could inform regional-scale decision-making on issues such as land-use regulations and MPA creation. Scenarios intended to inform decision-making at these or smaller scales in the future would benefit from vetting model assumptions through a planning process, and require that the Index be applied at the relevant spatial scale. Although hypothetical, the scenarios demonstrate several key aspects of the Index relevant to decision makers: 1) it responds quickly to management actions, giving initial ‘credit’ for those actions, and then further responds over time as the system (social, economic, and ecological) changes; 2) tradeoffs inherent in many decisions are captured by the Index (either explicitly as they are built into the Index or implicitly as they would emerge after management actions); and 3) the Index allows one to compare very different management actions in a transparent and quantitative way across different sub-regions, thus supporting strategic decision-making. The magnitude of expected change in the Index will necessarily be related to the scale of management action relative to the scale of assessment.

Such scenario analyses are also a key way that the Index can be used to explore potential implications of climate change on ocean health. As with the other scenario examples, because the Index does not model the future it cannot predict future ocean health. Instead, dynamic process models can be used to simulate ecological and social conditions, and then these results can be fed in as input parameters for calculating an alternate Index score. In this case, the Index can be used to indicate the likely overall ocean health in the future under status quo conditions and a changing climate. Additional management scenarios could then be layered on top of those outputs to better understand the likely effect of climate change on future ocean health.

Scenario analyses also illustrate how the Index can be used to identify and understand tradeoffs among goals. Some of these known tradeoffs are built into the architecture of the Index, for example in how increased (sustainable) fishing produces higher scores for food provision but lowers other goals due to its negative pressure on them. Other more complex, emergent tradeoffs become visible only when the Index is measured over time and one can track how goal scores change in similar or opposite directions. Because of the complexity of ecosystem responses, full attribution of a change in one goal causing a change in another goal is difficult, but such patterns can provide insight on where to direct further exploration of such possible tradeoffs. The ability to calculate past status scores, and then correlate changes in the Index with past management actions, illustrates a key way it can be used to assess management effectiveness. If the Index were adopted as a management tool, recalculating scores regularly could reveal whether management actions had the intended effect on both overall ocean health and particular goals. This objective demonstrates the power (and necessity) of having a quantitative, repeatable, transparent and comprehensive method for assessment.

The process of adapting the Index to finer geographic scales highlights its flexibility but also the limits to comparability of Index scores across scales. Most decision making focuses on optimizing outcomes for a region of interest (e.g., a particular country, or a state within a country), regardless of how other regions are performing, such that adapting the Index to the best available regional information is appropriate and ideal. However, it is human nature to ask how one is doing relative to others, and that desire for comparability can lead to misunderstanding of Index results if the comparisons are made across assessments at different scales (e.g., global version regional). Here we focus on results within and among U.S. west coast regions and minimize comparisons to global results for the U.S. for these exact reasons.

Many other assessment frameworks and tools have been applied to the U.S. west coast to evaluate different aspects of its health (see Table 5 for a summary of several prominent ones). Although it is instructive to compare the approaches to understand their strengths and weaknesses, it is important to note that each method was developed and applied for specific purposes, such that direct comparison among them is not always appropriate. Integrated Ecosystem Assessments (IEAs), Fisheries Ecosystem Plans (FEPs) and CalCOFI reports are all part of the Pacific Fisheries Management Council's (PFMC) decision process, such that those assessments are directly affecting and assessing management actions, however the Ocean Health Index is too new to have had a chance to be vetted and potentially included in the PFMC process. All of the other methods directly and explicitly assess the ecological and biophysical aspects of the system, whereas in the Ocean Health Index these assessments are not separately available because they are combined within integrated socio-ecological indicators. On the other hand, for this reason the Index is currently the only method to offer a fully integrated assessment. Most of the methods assess the full range of sectors active in the region, but the Ocean Health Index generally combines them together into overall goal measures rather than tracking individual sectors separately. Finally, the Ocean Health Index makes explicit the process of defining and setting quantitative reference points that establish when goals are fully achieved, whereas the other methods tend to rely on expert judgment and informal evaluations.

**Table 5:** Comparative summary of assessment tools and methods that have been applied to regions of the US West coast.

	OHI	IEA	PSP	FEP	CalCOFI
Ecological system assessed explicitly	**no**	**yes**	**yes**	**yes**	**yes**
Social system assessed explicitly	**no**	**yes**	**no**	**no**	**no**
Integrated assessment of socio-ecological systems	**yes**	**no**	**no**	**no**	**no**
Scalable to sub-regional level	**yes**	**yes**	**yes**	**no**	**yes**
Includes scenario analyses	**yes**	**yes**	**no**	**no**	**no**
Part of PFMC process	**no**	**yes**	**no**	**yes**	**yes**
Part of WCGA process	**no**	**yes**	**no**	**no**	**no**
Addresses most/all sectors	**yes**	**yes**	**yes**	**yes**	**no**
Combines all sectors into an overall quantitative assessment	**yes**	**no**	**no**	**no**	**no**
Reference points are explicitly delineated	**yes**	**no**	**no**	**no**	**no**

OHI, IEA and FEP methods have been applied to the entire west coast; PSP and CalCOFI are sub-regional assessments but are included for comparative purposes. Attributes which all or none of the methods achieve are not included in this table. See legend below for definition of acronyms.

Legend: OHI = Ocean Health Index; IEA = Integrated Ecosystem Assessment; PSP = Puget Sound Partnership; FEP = PFMC Fisheries Ecosystem Plan annual reports; PFMC = Pacific Fisheries Management Council; WCGA = West Coast Governors Agreement; CalCOFI = California Cooperative Oceanic Fisheries Investigations.

Another important policy implication of applying the Index is to help prioritize data collection and primary research efforts. Most monitoring focuses on biological impacts without connecting them explicitly to benefits that people want and need. The Index framework, by explicitly showing the connection between societal goals and the ability of the system to provide those goals, highlights the importance of collecting ecological, social, institutional, and economic data to monitor and inform management, and motivates all stakeholders to strive for a more sustainable human-ocean system.

Application of the Ocean Health Index to the US west coast not only provided an assessment of ocean health for the region but also guidance on the opportunities and challenges in applying and adapting the general Index framework to a regional setting. In the relatively data-rich US west coast, we were able to take advantage of the best available knowledge and information and make sub-regional assessments, sub-regions that share some ecological and socio-economic aspects but also show many differences that are important for defining management strategies. Such sub-regional assessments are likely to be important in most regions of the world. In particular, this downscaled, regional application of the Index offers a means and a medium for conversations among disparate marine use sectors by providing measures of diverse aspects of ocean health in a common currency.

## Supporting Information

File S1 Complete set of supplementary information, including supplementary methods, tables S1–S38, and figures S1–S2.
(DOCX)
