# Supplementary material for: Assessing the Health of the U.S. West Coast with a Regional-Scale Application of the Ocean Health Index
Source: PLoS One. 2014 Jun 18;9(6):e98995. doi: 10.1371/journal.pone.0098995 (PMC4062411; doi:10.1371/journal.pone.0098995)
Supplement: File S1 — Complete set of supplementary information, including supplementary methods, tables S1–S38, and figures S1–S2. (DOCX) [file pone.0098995.s001.docx]

Supplementary Information

Overview 3

Defining Ocean Health 3

Conceptual Framework 5

Reporting Units 6

Methods: Goal-Specific Models 8

A. Food Provision 8

Fisheries: 8

Mariculture: 11

Combining Sub-goals: 13

B. Artisanal Fishing Opportunities 14

C. Biodiversity 15

Species sub-goal: 16

Habitats sub-goal: 17

D. Carbon Storage 18

E. Coastal Protection 19

F. Sense of Place 19

Iconic Species sub-goal 20

Lasting Special Places sub-goal: 21

G. Clean Waters 22

H. Tourism and Recreation 23

I. Coastal Livelihoods and Economies 26

Livelihoods sub-goal: 26

Economies sub-goal: 27

J. Natural Products 28

Methods: Additional Analyses 29

Time Series of Status Scores 29

Sensitivity Analyses 30

Scenario Analyses 33

Specific Data Layers 35

Alien Invasive Species 35

Clean Water Enforcement and Regulation- 35

Climate Change Resilience 36

Coastal Access Points 36

Coastal Counties 37

Coastal Land and Ocean Area 39

Coastal population 39

Coastline and Coastal Zone Area 39

Ecological integrity 39

EPA Beach Closure data 39

FDA Action Levels 40

Fisheries Catch Totals 40

Fishing Pressure 41

Gas Price 41

Gas Price Solution Score 41

Genetic escapes 42

Grading the States Report (Governance) 42

Habitat Destruction, Intertidal Construction 43

Habitat Destruction, Intertidal Trampling 43

Habitat Destruction, subtidal soft-bottom trawling 43

Iconic species 43

Lenfest Report: An Economic, Legal and Institutional Assessment of Enforcement and Compliance in Federally Managed U.S. Commercial Fisheries 44

Mariculture Sustainability Index (MSI) Scores 45

Marine and Coastal Recreation Participation 45

Marine Jobs, Wages, and Revenue 45

Marine Protected Areas 47

Marine species 47

NatureServe Species Assessments 48

NOAA Fish Stock Sustainability Index (FSSI) Scores 49

NOAA MusselWatch Data 50

Noise and Light Pollution 51

Nutrients 51

Opportunity Index (SocialCapital) 52

pH 53

Public Land Ownership 53

Sea Surface Temperature (SST) anomalies 53

Shellfish Growing Areas 53

Shellfish Mariculture Yield 54

Socioeconomic Census Data 54

State Competitiveness Report 55

State level GDP 56

State level average wages 56

State level employment 56

Supplemental Marine Species 57

Trash 57

USDA NASS fertilizer data 58

UV 58

ALL HABITATS 58

Salt marshes 58

Sand Dunes 60

Seagrasses 61

Soft-bottom 61

Additional Tables 63

Supplemental Figures 87

# Overview

Here we provide a brief summary of the conceptual framework of the Ocean Health Index and then focus on explaining and detailing differences between this regional analysis and the global assessment (Halpern et al. 2012). Additional details can also be found in the supporting documentation for the global study.

## Defining Ocean Health

There continues to be some debate around the definition of ocean health that serves as the foundation for the Ocean Health Index, namely that a healthy ocean is one that sustainably delivers a range of benefits to people now and in the future (Halpern et al. 2012). Critique arises from some people’s discomfort with the idea that human values determine what constitutes ocean health, i.e. some people feel that health should instead be defined only by natural traits and thus equate with pristineness. However, from a purely pragmatic standpoint, defining healthy as pristine is irrelevant to nearly all policy contexts because they nearly always focus on inhabited regions and are tasked with managing human actions and values. In addition, the definition we use matches the one commonly used in the academic literature. The following quotations and statements from a wide range of sources support our position.

“A healthy ecosystem is one that provides the ecosystem services supportive of the human community, such as food, fiber, the capacity for assimilating and recycling wastes, potable water, and clean air. . . . Ecosystem health as a design and management goal can be contrasted with the more typical goal of ecological restoration – a return to some prior state of the system with lower human impact. As we have discussed, the ‘prior state’ goal is arbitrary and unrealistic, since humans have been an integral part of ecosystems for eons and the concept automatically precludes the possibility of a healthy ecosystem that includes humans.”

Costanza, R. 2012. Ecosystem health and ecological engineering. Ecological Engineering 45:24-29

“A healthy ecosystem is defined as a social-ecological unit that is stable and sustainable, maintaining its characteristic composition, organization, and function over time while remaining economically viable and sustaining human communities. The breadth of this definition indicates that ecosystem health is an integrative notion that acknowledges societal values in defining future desired conditions while relying on scientific criteria.”

Muñoz-Erickson, T. A., B. Aguilar-González, and T. D. Sisk. 2007. Linking ecosystem health indicators and collaborative management: a systematic framework to evaluate ecological and social outcomes. Ecology and Society 12(2): 6

“Ecosystem health assessments require analysis of linkages between human pressures on ecosystems and landscapes, altered ecosystem structure and function, alteration in ecosystem services, and societal response. Effective diagnosis requires exploring and identifying the most critical of these links.”

Rapport, D.J., R. Costanza and A.J. McMichael. 1998. Assessing Ecosystem Health. TREE 13:397-402

“A healthy Puget Sound includes a thriving natural world, high quality of life for people, and a vibrant economy. . . In a healthy ecosystem there are opportunities for growth and prosperity for people, while the other ecosystem benefits we enjoy can be sustained.”

Puget Sound Partnership. 2008. Puget Sound Action Agenda: Protecting and restoring the Puget Sound ecosystem by 2020. Olympia, WA.

“A healthy marine environment feeds our Nation, fuels our economy, supports our cultures, provides and creates jobs, gives mobility to our Armed Forces, enables safe movement of goods, and provides places for recreation. Healthy, productive, and resilient oceans, coasts, and Great Lakes contribute significantly to our quality of life.”

United States National Ocean Council (NOC). 2013. National Ocean Policy Implementation Plan.

“A healthy ecosystem is capable of providing ecological goods and services to people and to other species in amounts and at rates comparable to those that could be provided by a similar undisturbed ecosystem.”

Pew Oceans Commission. 2003. America’s living ocean: Charting a course for sea change. A report to the nation. Washington, D.C.

“Healthy oceans: cared for, understood and used wisely for the benefit of all, now and in the future.”

Commonwealth of Australia. 1998. Commonwealth of Australia. Australia's Oceans Policy.

“Oregon’s vision for its ocean and coastal health was articulated in 1973 by Statewide Planning Goal 19, Ocean Resources, which is ‘conserve marine resources and ecological functions for the purpose of providing long-term ecological, economic, and social value and benefits to future generations.’”

West Coast Governors’ Agreement on Ocean Health. September 18, 2006. http://www.westcoastoceans.org/media/WCOceanAgreementp6.pdf

” In this plan, a “healthy ocean” means that marine, coastal, and estuarine ecosystems, the watersheds that drain into these waters, the plant and animal communities therein, and the physical, chemical, and biological processes involved are diverse and functioning, and the economies and people dependent on them are thriving. A healthy ocean provides aesthetic, cultural, and recreational values. It also supports the character and quality of life of coastal communities and a vibrant, sustainable economy.”

West Coast Governors' Agreement on Ocean Health (page 10). 2008. Agreement on Ocean Health Action Plan. <http://www.opc.ca.gov/webmaster/ftp/pdf/docs/Documents_Page/Reports/WCGA_ActionPlan_low-resolution.pdf>

“Acknowledging the importance of achieving an overall long-term vision that ensures prosperous and healthy ocean and coastal environments providing for conservation, productivity and sustainable resource use.”

United Nations General Assembly. 2010. Sustainable development: Report of the Governing Council of the United Nations Environment Programme. Eleventh special session (24-26 February, 2010) . General Assembly. Official Records. Sixty-fifth session. Supplement 25. p.23.

A/65/25. 12 November 2010.

“Ecosystem well-being: A condition in which the ecosystem maintains its diversity and quality — and thus its capacity to support people and the rest of life — and its potential to adapt to change and provide a viable range of choices and opportunities for the future.”

FAO. 1995. Code of Conduct for Responsible Fisheries, Rome, 41p.

“‘Good environmental status’ means the environmental status of marine waters where these provide ecologically diverse and dynamic oceans and seas which are clean, healthy and productive within their intrinsic conditions, and the use of the marine environment is at a level that is sustainable, thus safeguarding the potential for uses and activities by current and future generations.”

European Parliament Council. 2008. Marine Strategy Framework Directive. Directive 2008/56/EC of the European Parliament and Council establishing a framework for community action in the field of marine environmental policy.

“A healthy aquatic ecosystem is one that can sustain its intended uses.”

USEPA Atlantic Slope Consortium (ASC). Brooks, et al. 2006. Integration of ecological and socioeconomic indicators for estuaries and watersheds of the Atlantic Slope. Final Report to the U.S, Environmental Protection Agency STAR Program.

## Conceptual Framework

The Index assesses the current and likely future status of ten goals for healthy oceans (Figs. 1 and 2 in the accompanying paper), and then averages the scores for these two status scores to give a single goal score. The current status is the present value relative to a specific reference point, with reference points established in one of four different ways (Samhouri et al. 2012). The approach to setting reference points for several of the goals was changed relative to approaches used in the global assessment (see Table S1).

The process of determining reference points is both scientific and socio-political (Samhouri et al. 2012). Science can provide information on thresholds or sustainable limits of delivering a goal, but often we do not know enough about such limits, and regardless, setting of reference points is always ultimately a social and political choice. A few examples can help illustrate this process. Decades of fisheries research has provided a wealth of information about how to set sustainable harvest levels, yet uncertainties, measurement error, and different levels of risk tolerance by policy makers and/or stakeholders leads to different specific reference points. For mariculture, we know that appropriate reference points are both a function of sustainable production densities (an active area of research) and total proportion of suitable coastal area available for mariculture (almost entirely a social decision). For habitat based goals (such as carbon storage and coastal protection), setting reference points requires information on past extent of habitats (which is often poorly known) and social decisions about how much habitat restoration is feasible and/or desired. For species-based goals (iconic species and species biodiversity), science provides a wealth of information about how to assess the viability of individual species, but it is ultimately a social decision about whether reference points should be set at pristine conditions, impacted but sustainable populations, or even to allow some level of threat or loss to species. In the goal descriptions below we provide details on how and why we selected each reference point. This transparency allows decision makers who may be interested in using the Index to evaluate if they agree with the reference points or would instead choose to change them.

We acknowledge that choices about parameter values in the main equation for the Index (equation 2 in the manuscript) and for many of the goal models, while informed by scientific understanding and the best available information, are ultimately subjective choices. Throughout the descriptions below we make an effort to clearly articulate where and why such choices were made, and note here that regional applications of the Index, such as was done here, could develop parameter values unique to the region based on input from community members, stakeholders, and decision makers.

**Table S1.** Comparison of type of reference point used for calculation of status for each goal and sub-goal in the global (Halpern et al. 2012) and U.S. west coast regional analyses.

| **Goal** | **Sub-Goal** | **Global Reference Point Type** | **Regional Reference Point Type (if different)** |
| --- | --- | --- | --- |
| Food Provision (*x_FP_*) | Fisheries (*x_FIS_*) | Functional Relationship |  |
|  | Mariculture (*x_MAR_*) | Spatial Comparison | Established Target |
| Artisanal Fishing Opportunities (*x_AO_*) |  | Functional Relationship | Established Target |
| Carbon Storage (*x_CS_*) |  | Temporal Comparison (historical benchmark) |  |
| Coastal Protection (*x_CP_*) |  | Temporal Comparison (historical benchmark) |  |
| Coastal Livelihoods and Economies (*x_LE_*) | Livelihoods (*x_LIV_*) | Temporal Comparison (historical benchmark) |  |
|  | Economies (*x_ECO_*) | Temporal Comparison (moving target) + Spatial Comparison |  |
| Tourism and Recreation (*x_TR_*) |  | Spatial Comparison | Temporal Comparison (moving target) |
| Sense of Place (*x_SP_*) | Iconic Species (*x_ICO_*) | Established Target |  |
|  | Lasting Special Places (*x_LSP_*) | Established Target |  |
| Clean Waters (*x_CW_*) |  | Established Target |  |
| Biodiversity (*x_BD_*) | Species (*x_SPP_*) | Established Target |  |
|  | Habitats (*x_HAB_*) | Temporal Comparison (historical benchmark) |  |

## Reporting Units

We subdivided the U.S. west coast into 5 sub-regions based on a combination of political (i.e. state) boundaries and biogeographic provinces (which divides California into three sub-regions). To produce the spatial boundaries of these reporting units (i.e., the GIS shapefiles associated with them) we first extracted the west coast USA Exclusive Economic Zone (EEZ) from the global region assessment (Halpern et al. 2012). We buffered the EEZ by 100 km to ensure we captured all land in San Francisco Bay and Puget Sound, where the land-sea mask is less certain, and then projected and extracted all ocean data for the global land-sea model to establish a definitive land-sea interface (i.e., coastline) . These data were then aligned with SRTM Water Body Data (Farr & al. 2007) and the Global Self-consistent, Hierarchical, High-resolution Shoreline Database, version 2 (Wessel & Smith 1996), as used previously (Halpern et al. 2009). For California, we updated the land-sea model by including all points within California state waters (CAOCEAN 2008). We then computed an offshore 3 nmi boundary per state for use with some goals, and extended these boundaries to the outer EEZ (200 nmi) boundary for use with other goals. The intersections of the EEZ waters for the US (to exclude Canadian and Mexican waters) and these two versions of sub-regional boundaries for the 3 states (CA, OR, WA) formed the five U.S. west coast sub-regions, with the 3 California sub-regions divided at county lines as shown in Fig. 2 in the accompanying paper.

Our focus is on assessment of the entire EEZ, divided into these five sub-regions, but we account for the fact that different goals play out at different scales. As such, sub-regional assessments represent a combination of assessments within state waters and assessments that include the full extent out to the EEZ boundary. Practically this means that some goals are assessed against a reference point that incorporates the area out to the boundary of the EEZ (e.g., fisheries, biodiversity) while other goals are assessed for area within nearshore state water boundaries (e.g., carbon storage, mariculture) even though the assessments are used to represent the score for the goal for the entire area out to the EEZ boundary. The scale at which each goal and sub-goal primarily act is described in Table S1a, and in more detail in goal model descriptions. Overall US west coast regional scores are the EEZ area-weighted averages of these sub-regional assessments.

**Table S1a**. Scale at which each goal primarily delivers its value, and thus the scale at which reference points are set (i.e., these scales determine the area used to assess current status relative to a reference point). Ultimately the sub-region scores represent the score for the area out to the boundary of the EEZ.

| **Goal** | **Sub-Goal** | **Primary Scale of Goal** |
| --- | --- | --- |
| Food Provision | Fisheries | EEZ |
|  | Mariculture | State waters |
| Artisanal Fishing Opportunities |  | State waters |
| Carbon Storage |  | State waters |
| Coastal Protection |  | State waters |
| Coastal Livelihoods and Economies | Livelihoods | EEZ |
|  | Economies | EEZ |
| Tourism and Recreation |  | State waters |
| Sense of Place | Iconic Species | EEZ |
|  | Lasting Special Places | EEZ |
| Clean Waters |  | State waters |
| Biodiversity | Species | EEZ |
|  | Habitats | EEZ |

Several datasets are at a county-level resolution, so we assigned each coastal county into one of the five regions (see Fig. 1 in main text). For our spatial analyses within the U.S. west coast region, we use an Albers Equal Area Conic projection (centered at -125**°** longitude with parallels at 30**°** and 50**°** latitude to minimize distortion of area) and a WGS84 datum.

# Methods: Goal-Specific Models

## Food Provision

The aim of this goal is to maximize the sustainable harvest of seafood in regional waters from wild-caught fisheries and mariculture. Wild caught fisheries harvests must remain below levels that would compromise the resource and its future harvest, but the amount of seafood harvested should be maximized within the bounds of sustainability, i.e., maximum sustainable yield (MSY). Similarly, mariculture practices must not inhibit the future production of seafood in the area, i.e. they must engage in sustainable practices, while maximizing the amount of mariculture that is possible and desired for a coastline that has many other uses as well. In short, sub-regions are rewarded for maximizing the amount of sustainable food provided and penalized for unsustainable practices and/or underharvest. A region may deliberately underharvest resources for conservation or other purposes, in which case its score for food provision would decrease, but its score for other goals (e.g., biodiversity, sense of place) might increase. Because fisheries and mariculture are separate industries with very different features, we track each separately as a unique sub-goal before combining them into the Food Provision goal.

Fisheries: The status of the Fisheries sub-goal (*x_FIS_*) was calculated as a function of the ratio between the single species current biomass at sea (B) and the reference biomass at maximum sustainable yield (B_MSY_), as well as the ratio between the single species current fishing mortality (F) and the fishing mortality at maximum sustainable yield (F_MSY_) (see Fig. S1), such that for n stocks:

(Eq. S1a)

where (bracketed letters next to specific equations given below refer to state-space within the Kobe plot shown in Fig. S1 and are provided for convenience and cross-referencing):

| $B'=\left\{\begin{matrix} \frac{\left(\frac{B}{Bmsy}\right)}{0.8} & \text{when }\frac{B}{Bmsy}<0.8 \\ 1 & \text{when 0.8 }\leq\frac{B}{Bmsy}<1.5 \\ \frac{\begin{matrix} \\ 3.35-\frac{B}{Bmsy} \end{matrix}}{1.8} & \text{when }\frac{B}{Bmsy}\geq1.5 \end{matrix}\right.$ | [A]  [B]  [C] | (Eq. S1b) |
| --- | --- | --- |

and

| $\begin{matrix} F'=\left\{\begin{matrix} 0 & \text{when }\frac{B}{Bmsy}<0.8\text{ and }\frac{F}{Fmsy}>\frac{B}{Bmsy}+1.5 \\ \frac{\frac{F}{Fmsy}}{\frac{B}{Bmsy}-0.2} & \text{when }\frac{B}{Bmsy}<0.8\text{ and }\frac{F}{Fmsy}<\frac{B}{Bmsy}-0.2 \\ \begin{matrix} \\ \frac{\frac{B}{Bmsy}+1.5-\frac{F}{Fmsy}}{1.5} \end{matrix} & \text{when }\frac{B}{Bmsy}<0.8\text{ and }\frac{B}{Bmsy}+\text{0.2 <}\frac{F}{Fmsy}<\frac{B}{Bmsy}+1.5 \\ 1 & \text{when }\frac{B}{Bmsy}<0.8\text{ and }\frac{B}{Bmsy}-0.2\leq\frac{F}{Fmsy}<\frac{B}{Bmsy}+0.2 \\ \frac{\frac{F}{Fmsy}}{0.8} & \text{when }\frac{B}{Bmsy}\geq0.8\text{ and }\frac{F}{Fmsy}<0.8 \\ 1 & \text{when }\frac{B}{Bmsy}\geq0.8\text{ and }0.8\text{ }\leq\frac{F}{Fmsy}<1.2 \\ \frac{2.5-\frac{F}{Fmsy}}{1.3} & \text{when }\frac{B}{Bmsy}\geq0.8\text{ and }\frac{F}{Fmsy}\geq1.2 \end{matrix}\right. & \end{matrix}$ | [a]  [b]  [c]  [d]  [e]  [f]  [g] | (Eq. S1c) |
| --- | --- | --- |

The weight, *w_i_*, is based on the relative average contribution of stock *i* to total catch, such that:

$w_{i}=\frac{\overline{C_{i}}}{\sum\overline{C}}$, (Eq. S2)$x_{\mathrm{FIS}}= \frac{{\sum_{1}^{i} S_{i}\mathrm{FSSI}}_{i}}{{\sum_{1}^{i} S_{i}\mathrm{FSSI}}_{R}}$

where $\bar{C}_{i}$ is the mean weight of each species *i* across all years of catch data (1950-2011) in each region, as an estimate of mean potential contribution of each species to total food provision, independent of yearly stochastic fluctuations of the population and possible recent declines. In this formulation the scoring for the *F’* component is reliant on the corresponding single species *B’* in order to account for responsible management actions taken to lower fishing effort when current at-sea biomass is below MSY (Fig. S1). This scoring method for the *B’* component produces lower scores for species that are both underfished and overfished as both of these conditions detract from the overall achievement of maximized food provision. The formulae show that a stock receives a score of zero if either it is completely depleted, i.e.$\left( \frac{B}{B_{\mathrm{MSY}}} \right)$ B/ B_MSY_ = 0, or strongly underfished, i.e. B/ B_MSY_ = 3.35, with 3.35 representing the local observed maximum value. Any past or future B/ B_MSY_ values greater than 3.35, as well as the species with this maximum value, would receive a zero score for food provision to denote that the species is severely underfished. However, given that underutilization of resources is generally easier to remediate than depletion, we apply an asymmetrical buffer for values of B/ B_MSY_ close to 1 that get assigned a perfect score, (i.e., overfished stocks achieve a perfect score if B/B_MSY_ is up to 0.2 points below 1 but underfished stocks achieve a perfect score if B/B_MSY_ is within 0.5 points of 1). Thus, overfished species negatively influence the long term sustainable delivery of the food provision goal more than do underfished species. The *F’* component produces lower scores for species where both underfishing and overfishing are occurring, but does not punish as severely for underfishing of stocks where B<B_MSY_ as it is assumed that F<F_MSY_ results from responsible management and these lower fishing mortality values should be considered optimal under current low at-sea biomass conditions.

We used B/B_MSY_ and F/F_MSY_ estimates from stock assessments for 41 different species across the study area, and included an estimated score for California market squid based on the California Department of Fish and Game’s market squid fishery management plan (see Table S2 for a full list of species included). These species accounted for ~59% of the total average catch across the NMFS catch time series data. Stocks were considered data poor when B/B_MSY_ and F/F_MSY_ estimates were unavailable. We tested the use of a recently published data-poor approach (Costello et al. 2012) to obtain B/ B_MSY_ scores for the remaining stocks. In order to validate the results we compared estimates with stock assessment values and found the latter to be outside the confidence bounds predicted by the model. This is most likely due to the fact that catch in the area has become strongly regulated since the mid-90s (Sustainable Fisheries Act, 1996), thus causing the model to confound a decline in catch that is driven by lower fishing effort with a decline in biomass at sea due to overfishing. Other estimates of overfishing are available for data poor stocks (Dick & MacCall 2010), however these only cover ~2% of overall catches. Therefore, we focus analyses only on assessed stocks. However, to assess the potential influence of including data poor stocks on this sub-goal, we also calculated the goal with all stocks included (described below, sensitivity analyses). For the assessed stocks, we used the most recent year of assessment as the ‘current’ year (estimates of B/B_MSY_ and F/F_MSY_ are available up to the year prior to the year of assessment), such that the status of this goal represents the combination of the ‘most recent years’ for these stocks (see Table S2 for most recent year of assessment).

For each fish stock, B/B_MSY_ and F/F_MSY_ scores were calculated for the entire region. Fisheries scores were then assigned to each sub-region based on the contribution of each species in each sub-region to the overall catch in that sub-region. These weights were assigned based on the average catch of each species across all years of catch data (1950-2011). The average catch over time was selected as it reflects the mean potential contribution of each species to total food provision, independent of stochastic fluctuations and possible recent declines. This means that each species gets a single score, and what differentiates the scores from region to region is which species are in that region and how much each of them contributes to the overall historical catch. These catch data were only available at the state level, so all sub-regions within California received the same status score.

**Table S2.** Full list of assessed species in the fisheries sub-goal and their most recent year of assessment.

| **Species** | **Assessment Year** | **Year for B/B_MSY_ and F/F_MSY_ values** |
| --- | --- | --- |
| Anoplopoma fimbria | 2011 | 2010 |
| Eopsetta jordani | 2011 | 2010 |
| Hexagrammos decagrammus | 2005 | 2004 |
| Doryteuthis (Loligo) opalescens | NA | Value estimated from literature |
| Merluccius productus | 2011 | 2010 |
| Microstomus pacificus | 2011 | 2010 |
| Ophiodon elongatus | 2009 | 2008 |
| Platichthys stellatus | 2005 | 2004 |
| Pleuronectes vetulus | 2007 | 2006 |
| Prionace glauca | 2009 | 2009 |
| Reinhardtius stomias | 2007 | 2006 |
| Sardinops sagax | 2011 | 2011 |
| Scorpaena guttata | 2005 | 2004 |
| Scorpaenichthys marmoratus | 2009 | 2008 |
| Sebastes alutus | 2011 | 2010 |
| Sebastes carnatus | 2005 | 2004 |
| Sebastes chlorostictus | 2011 | 2011 |
| Sebastes crameri | 2011 | 2010 |
| Sebastes diploproa | 2009 | 2008 |
| Sebastes elongatus | 2009 | 2008 |
| Sebastes entomelas | 2011 | 2010 |
| Sebastes flavidus | 2005 | 2004 |
| Sebastes goodei | 2007 | 2006 |
| Sebastes jordani | 2007 | 2006 |
| Sebastes levis | 2009 | 2008 |
| Sebastes melanops | 2007 | 2006 |
| Sebastes melanostomus | 2011 | 2010 |
| Sebastes mystinus | 2007 | 2006 |
| Sebastes paucispinis | 2011 | 2010 |
| Sebastes pinniger | 2011 | 2010 |
| Sebastes ruberrimus | 2011 | 2010 |
| Sebastolobus alascanus | 2005 | 2004 |
| Sebastolobus altevelis | No catch data, so dropped from analysis | |
| Squalus acanthias | 2011 | 2010 |
| Thunnus albacares | 2011 | 2011 |
| Thunnus obesus | 2011 | 2011 |
| Thunnus orientalis | 2010 | 2010 |
| Xiphias gladius | 2009 | 2009 |

The trend was calculated as the slope of the status scores over the past five years (2006-2011) only using data from assessed stocks. Most of the ecological and social pressures included in the Index were considered to have an impact on fisheries, noted in Table S31, as were most of the resilience measures, as indicated in Table S32.

Mariculture: The status of the Mariculture sub-goal was calculated as the sustainable production of shellfish biomass from mariculture relative to a target level of production for each state within the region. Species considered in the analysis were limited to shellfish (clams, mussels, and oysters) because these species comprise nearly all (estimated at 99%) of the current mariculture production of seafood in the region (Dumbauld et al. 2009, USDA 2005, California Department of Fish and Game 2008). Finfish mariculture currently only exists in Washington and represents only a small portion of total mariculture there (exact numbers are not available because data are proprietary). As such, the inclusion of any mariculture of finfish would not be relevant to 4 of the 5 sub-regions and would be negligible in the overall score for Washington because production scores are weighted by their relative contribution to overall food provision by tonnage. In addition, much of the finfish culture in Washington is not for food provision purposes, but rather for restocking or restoration purposes so is not included in the Food Provision calculation. Finally, production data for the few farms producing finfish in the region are mostly proprietary and could not be used in this analysis.

The Mariculture sub-goal (x_MAR_) is calculated as the current sustainably-harvested shellfish yield (Y_c_) within each region relative to the desired reference point yield (Y_r_), such that:

$x_{MAR}=\frac{Y_{C}}{Y_{r}}$ (Eq. S3)

$Y_{C_{k}}=\sum_{i} Y_{k,i}S_{i}$ (Eq. S4)

$Y_{M}=\left( \left( .5*\sum_{1}^{c} A_{c} \right)* \frac{\sum_{1}^{k} D_{k}}{k} \right)$ $- \sum_{1}^{k} \mathrm{WC}_{k}$ (Eq. S5)

where *Y_i_*$Y_{k}$ is the yield for each *i* shellfish species harvested in the region (*k*) in the most recent year available, 2008, e.g., *Y_i,2005_* is the yield for species *i* in 2005 (see below), *S_i_* $S_{k}$is the sustainability score for each *i* mariculture species, *FA_k_* is the potential farmable bay area in each state, *FA_T_* $D_{k}$is the total potential farmable bay area across the entire region, and 3.5 is the targeted production increase established by NOAA (i.e., 350%; see below).

Given the different contexts and potential for mariculture production in each sub-region, we set unique reference points for each one. To do this, we first used a national target established by NOAA of a 350% increase in production of shellfish from 2005 by 2020 and calculated this value for total west coast production. This desired 350% increase is based on growing domestic seafood consumption and a scenario for increasing national production developed by Nash (2004) based on “best available data, current technology, market demand, access to sites, and the advice of agency experts.” To establish a target production value for each state, we then distributed this increase in production among the three US west coast states based on the relative amount of farmable area within bays where shellfish mariculture currently occurs, per state (Dumbauld et al. 2009; see Table S3 below for breakdown of values), with all three sub-regions in California treated as a single aggregate sub-region and thus receiving the same score. As such, we assume that any growth in shellfish production capacity will come from the bays currently being farmed and that while the entire area of each bay cannot be dedicated to shellfish production, the proportion of each bay in each region that can be dedicated to mariculture is constant. We then used the total farmable bay areas in each state to distribute the shellfish production increase by multiplying the percentage of total farmable bay areas in each state by the 3.5-fold increase in yield over the entire region.

**Table S3:** Farmable Bay present in each state

| **State** | **Farmable Bay (hectares)** | **Percentage of Total area** |
| --- | --- | --- |
| CA | 10,470 | 12% |
| OR | 11,980 | 14% |
| WA | 63,770 | 74% |

The yield for all shellfish species harvested in each region comes from data compiled by the Pacific Coast Shellfish Growers Association. These data are the most comprehensive and inclusive available for the region, as many other sources do not include full production statistics on a per-farm or per-region basis due to the proprietary nature of these data. The sustainability score (*S_i_*) for each species (Table S4) comes from the Mariculture Sustainability Index (MSI) (Trujillo 2008) and is the average of three sub-indicators used in the MSI: wastewater treatment, the origin of feed, and the origin of seed. The three specific sub-indicators were chosen because they reflect the long-term sustainability of the mariculture practice, but are not reflective of the impacts the mariculture practices may have on the surrounding environment or species, as such impacts would not hinder the future production and sustainability of the mariculture sub-goal itself even though they might affect the delivery of benefits from other goals.

**Table S4:** Sustainability values for harvested shellfish species

| **Species** | **Sustainability Score (S_k_)** |
| --- | --- |
| **Clam** | 0.80 |
| **Mussel** | 0.93 |
| **Oyster** | 0.90 |

The trend was calculated as the slope of the actual shellfish production values in each state from 2005 to 2009. Pollution-related pressures and pH were the only ecological pressures included in the calculation of this goal along with the general social pressures, as indicated in Table S31. Resilience measures included water pollution enforcement and compliance scores as well as the social resilience measures indicated in Table S32.

Combining Sub-goals: The two sub-goals for the Food Provision goal were aggregated to produce a single goal score based on a proportional yield-weighted average, such that:

$x_{FP}=\left(w_{FP}*x_{FIS}\right)+(1-w_{FP})x_{MAR}$ (Eq. S6)

$w_{FP}=\frac{C_{T}}{\left(C_{T}+Y_{r}\right)}$, (Eq. S7)

where w is the weighting applied to each sub-goal based on the relative contribution of *C_T_*, the total wild caught yield of all species in the current year (2009), and *Y_r_*, the current sustainably-harvested shellfish yield in 2008 to overall food provision (See Table S5 for weightings used per region).

**Table S5:** Per-region weights used to combine Fisheries (FIS) and Mariculture (MAR) sub-goals for calculation of the Food Provision goal score in Eq. S6.

| **Code** | **Region** | **FIS** | **MAR** | **w (FIS)** | **1-w (MAR)** |
| --- | --- | --- | --- | --- | --- |
| CA1 | Northern California | 56,669 | 1,447 | 97.5% | 2.5% |
| CA2 | Central California | 56,669 | 1,447 | 97.5% | 2.5% |
| CA3 | Southern California | 56.669 | 1,447 | 97.5% | 2.5% |
| OR | Oregon | 90,218 | 1,008 | 98.9% | 1.1% |
| WA | Washington | 74,361 | 30,709 | 70.8% | 29.2% |

## Artisanal Fishing Opportunities

The artisanal opportunity goal seeks to measure the potential for local people to engage in artisanal-scale fishing, for either subsistence or cultural identity reasons. We adopt the definition of small-scale or artisanal-scale fishing used by NOAA, i.e. a fishery based on traditional or small-scale gear and boats, noting that this includes both intertidal, nearshore, and offshore fishing (NOAA Fisheries Glossary 2006). There are no data available on the number of people actively participating in artisanal fishing activities, nor a good approximation of what a reasonable reference condition would be were these data available. Instead, we consider physical and economic access to fishing opportunities and the condition of fish stocks in the region to be reasonable approximations of how well artisanal fishing opportunities are being provided in the region. This approach differs from what was done at the global scale (Halpern et al. 2012), where artisanal fishing opportunities were assessed as a function of need (based on the level of poverty present in a region) and the effectiveness of small-scale fisheries management, mainly focusing on artisanal opportunities for subsistence purposes.

Members of the public in the region fish artisanally from shore-based coastal access points (like beaches and jetties) as well as from boats. The key variables affecting access to these two modes of artisanal fishing differ and so we treat them differently in our assessment. Shore-based fishing is primarily constrained by physical access to fishing locations and is thus measured as coastal area within a mile of coastal access points. The target here is to maximize the amount of public access along the coast, therefore a perfect score results when each part of a region’s coastline has a coastal access point within 1 mile. We calculate these scores using a raster allocation model with 1-mile resolution intersecting at the coastline (see Fig S2 for example).

Access to boat-based artisanal fishing opportunities is instead more a function of economic constraints, in particular fuel prices, as boats can be trailored to distant boat launches or travel almost anywhere from a particular boat launch given no limitations on fuel prices. Thus we measure access for boat-based fishing as the 5-year trend in diesel prices as a percentage of median income. To measure the condition of fish stocks we use NOAA’s Fish Stock Sustainability Index (FSSI) score (NFMS 2012; described below). Thus the status of this goal is calculated as:

$x_{AO}=\frac{\frac{AP_{m}}{AP_{r}}+\frac{FSSI_{i}}{FSSI_{r}}+AE_{i}}{3}$ (Eq. S8)

$AE_{i}=\left(\frac{G_{r}}{I_{r}}-\frac{G_{c}}{I_{c}}\right)$ (Eq. S9)

Where *AP_m_* is the coastal area within 1 mile of an access point, *AP_r_* is the target reference for the amount of accessible coastal area and is set to 100%, *A_E_* is the current ratio of diesel gas price (*G_c_*) to median income *I_C_* relative to a reference ratio of gas price (*G_T_*) to median income (*I_T_*) from 5 years prior, *FSSI_i_*$\mathrm{FSSI}_{i}$ is the weighted average score in the region, and *FSSI_r_*$\mathrm{FSSI}_{R}$ is the maximum potential FSSI score.

The FSSI is considered a sustainability performance measure for important commercial and recreational fisheries throughout the U.S. It is based on whether there is adequate knowledge available to determine if a species is being overfished, if overfishing is occurring, and whether the stock biomass is at or above 80% of the biomass that produces maximum sustainable yield (B_MSY_) (See Table S6 for complete scoring criteria). FSSI scores increase as the stock status of selected fisheries increases, as overfishing decreases, and as stock levels increase to values that allow for maximum sustainable yield. The rationale for using this index is that stocks in better condition provide more sustainable opportunity for artisanal/recreational fishing. For the region, the FSSI values are calculated for the 54 species (see Table S36) that are considered most important to commercial and recreational fisheries under Pacific Fisheries Management Council jurisdiction. While this accounts for only 36% of the commercial species currently fished in the region, these stocks are assumed to be representative of overall regional status and account for ~80% of total regional commercial landings in 2011 (77% of catch based on average landings from 1950-2011). NOAA updates and releases FSSI scores for all regions on a quarterly basis. For this status and trend, the fourth quarter scores in each year were used, with 2011 as the current year.

**Table S6**. NOAA FSSI Scoring criteria and points awarded.

| **FSSI Criteria** | **Points Awarded** |
| --- | --- |
| “Overfished” status is known | 0.5 |
| “Overfishing” status is known | 0.5 |
| Overfishing is not occurring (for stocks with known “overfishing” status) | 1 |
| Stock biomass is above the “overfished” level defined for the stock | 1 |
| Stock biomass is at or above 80% of the biomass that produces maximum sustainable yield (BMSY) | 1 |
| **Total** | **4** |

The trend is calculated from the slope of the status scores over the last five years (2006-2011). The pressures include most of the ecological pressures and all of the social pressures as noted in Table S31. Most of the resilience measures were also included as indicated in Table S32.

## Biodiversity

People value marine biodiversity for its existence value. Biodiversity can also play a supporting role in the provision and sustainability of many other public goals; however this supporting role is not captured here. Instead, it is included in the resilience dimension, which is used in calculating the likely future state, for other public goals. Here we measured biodiversity through two sub-goals: habitats and species. Because the status of only a small portion of species has been assessed, we also measure the status of habitats as a proxy for the many species that rely upon these habitats. A simple average of these two sub-goal scores was used to obtain a single biodiversity goal score.

Species sub-goal: As was done in the global analysis, species status was calculated using each species’ conservation risk category, as determined by the International Union for Conservation of Nature (IUCN) Global Marine Species Assessment, for all species for which distribution maps were available (from a global 0.5° grid). These data were clipped to the extent of the region, allowing for the assessment of 141 species throughout the range. These data are based on global rather than regional assessments because regional data currently do not exist for most species. Though this is a very small sub-sample of the actual marine species present in the range, it represents the most comprehensive species status dataset available for the region and is used as a proxy of overall species status in the area. We were able to supplement these data with results from a recent assessment of fish species based on IUCN criteria for 23 additional species occurring within the U.S. west coast (See Table S26 for list of fish species added) (Davies & Baum 2012), resulting in a total of 164 species assessed in this sub-goal.

The target reference point for this goal is to have all species within the region classified with a risk status of Least Concern. This goal also requires setting a lower limit (i.e., when status = 0) because setting this lower bound as the point at which every single species is gone is not meaningful to human values. Instead, we set this lower bound as when 75% of species are extinct, a level comparable to the five geologically documented mass extinctions (Barnosky et al. 2011). This score could also result from fewer extinct species but more in highly threatened categories; here we treat these scenarios equivalently. The consequence of this choice was explored elsewhere (Selig et al. 2013). Weights for each threat category are assigned to species by their established IUCN threat category based on the weighting scheme developed by Butchart et al. (Butchart et al. 2007) (see Table S7 for IUCN threat categories and weights). The original weighting scheme developed by Butchart et al. (2007) to quantify extinction risk, which ranged from 0-5 (extinct = 5), was rescaled from 0-1 and inverted to represent a lack of extinction risk for our purposes. See Halpern et al. (2012) for the full methodological description.

**Table S7.** IUCN risk categoriesle SX. IUCN risk category and weight as and weights derived from weights developed by Butchart et al. (2007).

| **Risk Category** | **IUCN code** | **Weight** |
| --- | --- | --- |
| Extinct | EX | 0.0 |
| Critically Endangered | CR | 0.2 |
| Endangered | EN | 0.4 |
| Vulnerable | VU | 0.6 |
| Near Threatened | NT | 0.8 |
| Least Concern | LC | 1.0 |

The status for the species sub-goal was calculated as the area-weighted average species risk status, as was done in Halpern et al. (2012). The threat category weight (*w*) for each species (*i*) is summed for all of the *M* 0.5 degree grid cells (*c*) and divided by the total number of species (*N*) within each cell. The resulting score is an area-weighted mean across all species *i* within cell *k*. These values are summed across all cells in each *k* sub-region and divided by the total area within the sub-region (*A_T_*) such that:

$x_{SPP}=\frac{\sum_{k=1}^{M} \left(\frac{\sum_{i=1}^{N} w_{i,k}}{N}\right)*A_{c}}{A_{T}}$ (Eq. S10)

The trend was calculated using available trend values assigned by IUCN for assessed species (N=53), with increasing populations receiving a score of 0.5, stable populations a 0, and decreasing populations receiving a -0.5. Trends were aggregated in the same way as the status scores above. All pressures were applied in the species sub-goal except human pathogens and gas prices (see table S31 for full list). Most resilience measures were also applied, except climate change regulations and gas prices. We also did not include the ecological integrity measure as it utilizes the same IUCN risk category data applied in the status calculation (see table S32 for full list).

Habitats sub-goal: The status of the habitat sub-goal ($x$_HAB_) was calculated using publicly available data for habitats including salt marshes, seagrasses, sand dunes, and soft-bottom habitats. These habitats were chosen because they represent a large portion of regional coastal and marine environments and have data with relatively comprehensive temporal and spatial coverage. Other important habitats such as kelp forests, rocky reefs, and the rocky intertidal could not be included due to lack of data on current and/or past spatial extent and condition. The status of the habitat subgoal ($x$_HAB_) is calculated based on the current condition (C_C_) compared to the reference condition (C_r_) of each $k$habitat such that:

$x_{HAB}=\frac{\sum_{k}^{N} \left(\frac{C_{C}}{C_{r}}\right)}{N}$ (Eq. S11)

In the global study, the current condition of salt marshes, seagrasses, mangroves and corals was compared to a reference year that is intended to represent optimal conditions (1980 for salt marshes and sand dunes, varied by site for seagrasses; Halpern et al. 2012). However, reliable, comprehensive habitat extent data prior to the 1990s are unavailable for most coastal regions within the U.S. Estimates of habitat loss since European settlement have been extrapolated in some regions for some habitats. While the habitat extent from the 1990s would represent a very un-ambitious target, a pre-industrialized reference point for habitat extent is considered an unrealistic goal under current conditions. To establish our temporal reference points we instead set our reference uniquely for each habitat, as a percentage (50%) of pre-industrialized habitat coverage for salt marshes, or habitat extent between the 1950s and 1960s for sand dunes. For seagrasses and soft bottom habitats we utilized relevant pressures as a proxy of habitat condition. These reference points were selected to provide ambitious yet realistic goals following principles for desirable reference point qualities (Samhouri et al. 2012). See specific habitat layer descriptions for full data source information and modeling details.

## Carbon Storage

Coastal habitats play a significant role in the global storage of organic carbon as they have the highest per-area storage rates of any habitat (McLeod et al. 2011). While the pelagic oceanic carbon sink plays a large role in the sequestration of anthropogenic carbon, the pelagic ocean mechanisms are not amenable to local or regional management intervention. This assessment instead focuses on the status of coastal marine habitats with high carbon storage capacity. Globally, coastal marine habitats occupy <0.05% of ocean area, but sequester at least 50% of the total carbon storage in ocean sediments (Nellemann et al. 2009). The destruction of these marine habitats has been shown to generate large quantities of carbon emissions (Donato et al. 2011), damaging the overall health of coupled marine systems. Our assessment here focuses on the two habitats known to provide the most significant and measurable amount of carbon sequestration in the region: salt marshes and seagrass beds. The status of this goal is measured as a function of the condition of each habitat (*C_c_*) relative to a reference condition (*C_r_*), and the relative contribution of each habitat (*k*) to overall sequestration in the area, measured as the area covered by each habitat *A_k_* relative to total coverage of all *n* sequestering habitats in the sub-region, *A_T_*. $A_{T}).$ As was done in the global analysis, we assume here that each habitat has the same rate of carbon sequestration. The status is thus measured using the same functional relationship as was used in the global analysis:

$x_{CS}=\sum_{1} \frac{C_{c}}{C_{r}}\cdot\frac{A_{k}}{A_{T}}$ (Eq. S12)

To calculate the habitat condition scores we used different methods depending on the habitat type and available data (see section C and full description below where habitat data layers are described).

The trend for this goal is calculated as the slope of the change in status as outlined in the general methods provided above. Pressures on salt marshes include pollution, intertidal habitat destruction, and invasive species, as well as governance and social capital pressures. Resilience measures include clean water enforcement and regulation measures, protected area coverage, and governance and social capital measures (quantified with the Social Capital/ Opportunity Index) were included. For seagrasses, we included pollution, intertidal habitat destruction, invasive species, and sea surface temperature as ecological and physical pressures likely to impact seagrasses, as well as governance and social capital pressures (see Table S31). For resilience measures, Clean Water Act enforcement and other regulatory measures, protected area coverage, climate change regulations, governance and social capital measures were utilized (see Table S32).

## Coastal Protection

This goal assesses the role of marine associated habitats in protecting coastal areas that people value, both inhabited (e.g. cities) and uninhabited (e.g. park). In the U.S. west coast we measured the role of salt marshes, seagrasses, and sand dunes as these habitats provide the most significant and measurable amount of biological coastal protection (we do not evaluate protection afforded by human-made or geological features). Ideally one would also know the value of the land and vulnerability of inhabitants being protected by these habitats, as has been done for estimates of current coastal protection (Arkema et al. 2013), but to do this within the Index requires knowing at very high spatial resolution where each habitat type currently is and where it used to be (for setting reference points) as well as a measure of the value of what is/was protected by the habitats in each time period. We currently do not have this information, and thus this goal assesses the potential value of coastal protection provided by habitats.

The status of this goal was calculated as the condition of each habitat relative to a reference condition and the ranked protective ability of each habitat, such that:

$x_{CP}=\sum_{k} \left(\alpha_{k}\frac{C_{c,k}}{C_{r,k}}\right)$ (Eq. S13)

$\alpha_{k}=\frac{w_{k}A_{k}}{\sum_{k} \left(w_{k}A_{k}\right)}$ (Eq. S14)

$w_{k}=\frac{r_{k}}{\sum_{k} r_{k}}$ (Eq. S15)

where *α_k_* is the area-weighted rank for habitat *k*, *r_k_* is the protective rank for habitat *k*, *A_k_* is the area of habitat k, *C_k_* is the current (*c*) and reference (*r*) conditions for habitat *k*. Protective habitat ranks are the same as those used in the global analysis and come from the Natural Capital Project (Natural Capital Project 2011), which ranks the protective ability of salt marshes as 3, sand dunes as 2, and seagrasses as 1.

Salt marsh, sand dune, and seagrass extent and trend were calculated in the same way as was done in the biodiversity model. We identified a subset of pressures that may affect these protective habitats and the delivery of this goal including pollution, intertidal habitat destruction, invasive species, and sea surface temperature, as well as governance and social capital pressures (see Table S31 for full details by habitat). Resilience measures included a combination of clean water enforcement and regulation, protected area coverage, climate change regulations, governance and social capital measures depending upon the specific habitat (see Table S32 for full details by habitat).

## Sense of Place

The Sense of Place goal aims to capture aspects of the coastal and marine system that contribute to a person’s sense of cultural identity. This goal is difficult to measure quantitatively because many attributes that define one’s cultural identity are not measured. Several reasonable proxy measures of aspects of sense of place do exist, and we used those here. To measure how well this goal is being delivered, we focused on two components of how people connect with the ocean: iconic species and lasting special places. The overall sense of place goal score is then the arithmetic mean of the two sub-goals scores.

Iconic Species sub-goal**:** Iconic species are defined as those that are relevant to local cultural identity through one or more of the following: 1) traditional activities such as fishing, hunting or commerce; 2) local ethnic or religious practices; 3) existence value; and 4) locally-recognized aesthetic value (Burns et al. 2006, Halpern et al 2012). To define a list of iconic species specific to the region, local experts were consulted and a list of 17 species based on the above criteria was compiled (see Table S16 for list of species). This list includes species that live either completely or primarily in the ocean.

To assess the status of these iconic species within the region we used the same methods outlined in the global assessment (Halpern et al. 2012), but replaced the global IUCN source data with regionally specific species assessments provided by NatureServe (www.natureserve.org). Like the IUCN assessments, NatureServe uses 6 categories to assess the status of species. These categories have been shown in the literature to correlate well with IUCN categories at the global scale (Goodenough 2012; Keith et al. 2004; Mehlman et al. 2004; Regan et al. 2004). Thus we scored the status categories in the same manner as was done with the IUCN categories in the global assessment utilizing weights developed by Butchart et al. (2007). This weighting system was also used in the species sub-goal for biodiveristy in this regional assessment (see Table S21 for categories and weights).

The IUCN species assessments were used for the calculation of the biodiversity goal because they cover a broad range of species chosen in a systematic way, regardless of conservation concern or charisma. These are more likely to be broadly representative of the status of unassessed species. The NatureServe database rather than the IUCN global assessments was selected for the iconic species sub-goal rather than the IUCN global assessments because NatureServe has regionally specific assessments of a sub-set of species whereas IUCN provides global-scale assessments. Furthermore, NatureServe includes assessments for most of the species identified as iconic for the U.S. west coast while IUCN has only assessed the status of 7 of the iconic species (and the trend for only 5).

The status of the species sub-goal (*x_SPP_*) is measured as the weighted average of species extinction risk weights, such that:

$x_{ICO}=\frac{\sum_{i=1}^{6} S_{i}*w_{i}}{\sum_{i=1}^{6} S_{i}}$ (Eq. S16)

where *S_i_* is the number of species in each threat category$i$, and *w_i_* is the risk status weights assigned to each of these categories. This formulation essentially gives partial credit to species that still exist but are vulnerable or imperiled. The target reference point here is that all species are assessed as “Secure”, giving a goal score of 1.

The trend was calculated as the average of the recorded categorical trend for all assessed iconic species, giving scores of 0.5 for increasing population, 0.0 for stable populations, and -0.5 for decreasing populations. Because all species are affected by pressures from human activities both on land and at sea, we assessed pressures based on all ecological pressure categories (except human pathogens) and all social pressures (except diesel gas price; see Table S31 for full list). All resilience measures were used except climate change regulations (see Table S32 for full list).

Lasting Special Places sub-goal: As was done in the global assessment, the lasting special places sub-goal focuses on the conservation status of geographic locations that hold significant aesthetic, spiritual, cultural, recreational, or existence value for people. Measuring the status of this goal proved difficult as places hold special value for people for myriad reasons and personal associations with places are difficult to quantitatively assess. Ideally one would have (or develop) a list of all the places that people within a region consider special, and then assess what percent of and how well those areas are protected. No such data exist. For the regional assessment we chose to focus on the protection status of all marine and coastal areas, as was done in the global assessment, under the assumption that efforts to protect places suggest that they are significant to people. We recognize that for some individuals, placing regulations on an area to protect it may prevent them from the very activities that made those places special to them in the first place, such that higher protection may not represent a healthier state in their view. However, we use this approach here because it is viable (i.e. data exist to calculate it), and because, although imperfect, it does convey some information about lasting special places. If a place is special and appropriate regulations/protection are placed on that location (for example, limited access, fishing, etc.), we feel that this ensures long-term sustainability of a place people care about.

To calculate the lasting special places goal we focus both on the marine environment, through the assessment of fully protected marine protected areas, and the coastal terrestrial environment, through the assessment of the percentage of lands under public protection. The status calculation is therefore:

$x_{LSP}=\frac{\frac{MPA_{3nm}}{0.3*A_{3nm}}+\frac{MPA_{EEZ}}{0.3*A_{EEZ}}+\frac{TA_{PA}}{0.3*TA}}{3}$ , (Eq. S17)

where *MPA_3nm_* is the marine area within coastal waters of the sub-region (from the coast to 3nm) that falls within a fully-protected marine protected area, *MPA_EEZ_* is the amount of offshore area (3-200nm offshore) within each subregion that is within a fully-protected MPA, *A_3nm_* is the total nearshore marine area within that sub-region, *A_EEZ_* is the total offshore area within that sub-region, *TA_PA_* is the terrestrial area within 1 mi of the coast that is publicly owned and protected and/or managed, and *TA*$\mathrm{TA}_{\mathrm{tot}}$ is the total terrestrial area within 1 mi of the coast. The 30% target (i.e., the 0.3 multiplier in the denominator of the fractions in the numerator of Eq. S17 used to calculate the reference state) for both marine and terrestrial areas is somewhat arbitrary; for marine protected area coverage we used guidelines from the Fifth Worlds Park Congress (Hughes et al. 2003), acknowledging that those guidelines relate to biodiversity conservation rather than special places, and we simply matched this value for terrestrial areas based on the idea that people want some fraction of land to be owned and managed in the public’s best interest into perpetuity.

The trend is calculated based on the change in the total marine area protected in each sub-region from 2008 to 2012 (no data were available to calculate trends in terrestrial managed areas). Pressures on this sub-goal include most pollution and habitat destruction pressures, as well as governance and social capital (see Table S31). Resilience measures included clean water enforcement and regulations, MPA coverage, governance and social capital (see Table S32).

## Clean Waters

People enjoy the presence of unpolluted estuarine, coastal, and marine waters for their aesthetic value and because they help avoid detrimental health effects to humans and wildlife. To calculate this goal we measure the status of 4 different contributors to water pollution: nutrients, pathogens, chemicals, and trash. As was done in the global assessment, we focus on assessment of nearshore waters. Although clean waters are relevant and important anywhere in the ocean, coastal waters drive this goal both because the problems of pollution are concentrated there and because people predominantly access and care about clean waters in coastal areas. We also have severe data limitations for open ocean areas with respect to measures of pollution.

The status of this goal (*x_CW_*) is calculated as the geometric mean of 4 components, such that:

(Eq. S18)

where *a* = 1 – (mean weighted pathogen score), *u* = 1 – (nutrient input score), *l* = 1 – (chemical input score), and *d* = 1 – (marine debris input score).

For the nutrients component we used the nutrient input layer developed by Halpern et al. (2009), which modeled nutrient plumes from fertilizer and nitrogen input into watersheds. Present value of nutrients was then calculated as 1-x where x is the zonal mean out to 3 nm in each sub-region. For the pathogens layer we used EPA beach closure data to determine the percent of beach days of which water samples exceed national pathogen standards by region (these are county level data that we aggregate to our sub-regions using the mean value). We then rescale these data on a 0-1 scale so that the maximum exceedence value (0.37) is set to 1.0. Present value of pathogens is then calculated as 1 – x per state, where x is the rescaled exceedence value for each region in 2010, the most recent year of data. For the trash layer we used beach cleanup data from the Ocean Conservancy (www.oceanconservancy.org/our-work/marine-debris). These data measure the amount of trash cleaned up in each state on their yearly International Coastal Cleanup day. We assumed that data represent all trash present on the beach (i.e., that total trash collected was independent of effort, measured as the number of people participating in the clean up). Thus we standardized trash without respect to effort as the density, i.e. pounds per mile of coastline, for each state in each year, setting 0 pounds per mile as the target and rescaling all values from 0 to 1 so that the highest density of trash recorded over the entire time period for all states in the U.S. is 1.0 (Oregon in 2010) and delivers a score of zero using the formula 1 – x.

To calculate a score for the chemicals layer we used NOAA MusselWatch data which consist of marine sediment and bivalve tissue samples collected from U.S. coastal and estuarine regions from 1986-2009 (O’Conner & Lauenstein 2005, 2006). These samples have measured concentrations for major and trace elements and a suite of organic chemical constituents. Our analysis filters these data to include only the bivalve tissue samples from sampling sites in California, Oregon, and Washington. For the present value of chemicals we focus on 11 contaminant categories determined to be most influential on human well-being and selected by the NOAA State of the Coast report to be of significant concern: Arsenic, Cadmium, Chlordane, Chromium, DDT, Dieldrin, Lead, Mercury, Mirex, Nickel, and PCB. Although this is a subset of all chemical pollutants, these in situ measurements are temporally and spatially replicated and widely used by many monitoring and assessment groups. We scored each sample categorically as follows, using specific threshold values for tissue samples that come from the NOAA State of the Coast Report and are originally derived from FDA Action Levels: 0.0 (bad), 0.5 (ok), and 1.0 (good) (see Table S8 for NOAA derived Chemical threshold values). We aggregated the scores by computing the mean for each contaminant category, grouped by state and year.

**Table S8**. MusselWatch NOAA State of the Coast chemical threshold values.

| **Contaminant** | **ppm (Good)** | **ppm (OK)** | **ppm (Bad- Above FDA Action Level)** |
| --- | --- | --- | --- |
| **Arsenic** | 0 | 43 | 86 |
| **Cadmium** | 0 | 2 | 4 |
| **Chlordane** | 0 | 0.1 | 0.3 |
| **Chromium** | 0 | 6.5 | 13 |
| **DDT** | 0 | 2.5 | 5 |
| **Dieldrin** | 0 | 0.1 | 0.3 |
| **Lead** | 0 | 0.8 | 1.7 |
| **Mercury** | 0 | 0.5 | 1 |
| **Mirex** | 0 | 0.1 | 0.1 |
| **Nickel** | 0 | 44 | 80 |
| **PCB** | 0 | 1 | 2 |

Trend data for the nutrients layer comes from USDA NASS fertilizer data in units of “acres applied” reported by county for the 3 most recent data years (1997, 2002, 2007). The minimum and maximum acres applied for any county in any year determine the min/max reference points to rescale the layer from 0 to 1, with 1 being the highest amount applied. The scores for 1997, 2002, and 2007 are then calculated on a per county basis as 1-x, where x is the rescaled acres applied per total county acreage. The trend per county is then calculated using the slope in scores for the 3 available years. The trend in pathogens data is calculated as the change in status scores from 2006-2010. Trend for the chemicals layer comes from the same MusselWatch categorical data, with trends calculated as the slope of a linear regression for values between 2000 and 2008 for each state. For the trash layer the trend is calculated over the status scores from 2007-2011.

## Tourism and Recreation

This goal captures the value people have for experiencing and taking pleasure in coastal areas. There are many ways to potentially measure the delivery of this goal. In the global analysis (Halpern et al. 2012), data on international arrivals were used as a proxy for the value of tourism and recreation in each region, as this was the most comprehensive data available on a global scale. Here we instead chose to focus on the changes in participation in 19 different marine and coastal specific recreational activities over time (see Table S9 for all recreational activities included). These data come from the National Survey on Recreation and the Environment (NSRE), which has been conducted 8 times nationally since 1960, with the most recent data available for coastal and marine specific activities from 2000. Since these data were no longer collected after 2005, Leeworthy et al. (2005) provided participation rates forecasted nationally for 2005 and 2010 using logit models of participation as a function of socioeconomic factors by activity type (see table S9a for full list of socioeconomic factors included; for full methodological detail see Leeworthy et al. 2005). Their models were applied using projected census data for 2005 and 2010 to estimate participation in each activity, but we instead used actual 2010 US census data (U.S. Census Bureau 2010). We regionalized the models for each state and for each activity using a 6 step process, which involved: 1) extracting the coefficients for the 33 input variables in the Leeworthy et al. (2005) models, 2) extracting the input data from the US Census for 2000, 3) calculating the participation rates, per activity, using coefficients and input data, 4) extracting real values for participation rates for 2000 and re-calculating a new adjustment factor, i.e. a modifier for the intercept, from the difference between observed and predicted values, 5) extracting input data for 2010 from the US Census, and finally 6) applying the model with the new adjustment factor and the 2010 input data.

Table S9: Participation rates for 19 activities.

|  |  | 2000 | | | 2010 | | |
| --- | --- | --- | --- | --- | --- | --- | --- |
| **Activity** | **Activity Code** | **CA** | **OR** | **WA** | **CA** | **OR** | **WA** |
| Visiting Beaches | BeachSW | 6.11 | 1.01 | 0.98 | 6.15 | 1.10 | 1.08 |
| Bird Watching in Saltwater Surroundings | BirdsSW | 1.25 | 0.28 | 0.42 | 1.28 | 0.35 | 0.48 |
| Canoeing | CanoeSW | 0.09 | 0.01 | 0.08 | 0.05 | 0.06 | 0.14 |
| Personal Watercraft Use | JSkiSW | 0.33 | 0.00 | 0.01 | 0.34 | 0.15 | 0.16 |
| Kayaking | KayakSW | 0.21 | 0.01 | 0.07 | 0.28 | 0.17 | 0.24 |
| Motorboating | MBoatSW | 0.75 | 0.07 | 0.23 | 0.77 | 0.14 | 0.32 |
| Hunting Waterfowl in Saltwater Surroundings | MhuntSW | 0.05 | 0.00 | 0.01 | 0.19 | 0.17 | 0.22 |
| Viewing or Photographing Scenery in Saltwater Surroundings | OVSW | 2.03 | 0.51 | 0.58 | 2.05 | 0.58 | 0.65 |
| Rowing | RowSW | 0.14 | 0.01 | 0.02 | 0.16 | 0.11 | 0.13 |
| Sailing | SailSW | 0.53 | 0.01 | 0.09 | 0.59 | 0.12 | 0.22 |
| Scuba Diving | ScubaSW | 0.14 | 0.01 | 0.04 | 0.28 | 0.23 | 0.29 |
| Saltwater Fishing | SFish | 1.32 | 0.16 | 0.24 | 1.32 | 0.20 | 0.29 |
| Snorkeling | SnorkSW | 0.34 | 0.02 | 0.02 | 0.40 | 0.16 | 0.17 |
| Surfing | Surf | 0.54 | 0.00 | 0.01 | 0.61 | 0.17 | 0.21 |
| Swimming | SwimSW | 4.07 | 0.31 | 0.34 | 4.10 | 0.42 | 0.45 |
| Visiting Waterside Besides Beaches | WaterSW | 0.73 | 0.14 | 0.21 | 0.77 | 0.20 | 0.28 |
| Water-skiing | WSkiSW | 0.13 | 0.01 | 0.03 | 0.14 | 0.09 | 0.15 |
| Wind Surfing | WSurfSW | 0.04 | 0.00 | 0.01 | 0.10 | 0.18 | 0.21 |
| Viewing Other Wildlife in Saltwater Surroundings | WVSW | 1.24 | 0.33 | 0.36 | 1.25 | 0.39 | 0.42 |

**Table S9a**. Socioeconomic factors included in the Leeworthy et al. 2005 analysis.

| **Factors** | **CODE** |
| --- | --- |
| Total Population |  |
| Total Pop (Civilian Non-Institutionalized) |  |
| Total Pop (16+) |  |
| **Age** | |
| 16-24 years old | age16_24 |
| 25-34 years old | age25_34 |
| 35-44 years old | age35_44 |
| 45-54 years old | age45_54 |
| 55-64 years old | age55_64 |
| 65+ years old | age65p |
| **Coastal County Resident** | ccounty |
| **Urban Resident** | urban |
| **Educational Attainment** | |
| Less than High School | educ11 |
| High School | educhs |
| Some College or College Degree | educcoll |
| Master's, Professional Degree, or Doctorate | educgrad |
| Other | educoth |
| **Household Income** | |
| $0-$25,000 | inc25 |
| $25,000-$50,000 | inc50 |
| $50,000-$100,000 | inc100 |
| $100,000+ | inc100p |
| Income Missing | incmiss |
| **Race/Ethnicity** | |
| White, not Hispanic | white |
| Black, not Hispanic | black |
| Native American or Pacific Islander, not Hispanic | native |
| Asian, not Hispanic | asian |
| Hispanic | hispanic |
| Other, 2+, etc. |  |
| **Sex** | |
| Male | male |
| **Census Division** | |
| New England | cendiv1 |
| Middle Atlantic | cendiv2 |
| South Atlantic | cendiv3 |
| East South Central | cendiv4 |
| West South Central | cendiv5 |
| East North Central | cendiv6 |
| West North Central | cendiv7 |
| Mountain | cendiv8 |
| Pacific | cendiv9 |

The goal is then calculated as:

$x_{TR}=\frac{\sum_{i=1}^{19} P_{c}}{\sum_{i=1}^{19} P_{r}}$ (Eq. S19)

where *P_c_* is the estimated current participation rate in 2010 per 100 people in each recreation activity$i$, and  *P_r_* is the observed participation rate per 100 people in 2000. The reference point is thus based on no-net-loss, i.e. that the rate of overall participation across all individual recreation activities remains the same or improves. We set the maximum score possible at 1 for all regions. For the trend, we calculated the rate of change in participation across all recreation activities from 2000 to 2010. Pressures on this goal included water pollution pressures, and all social pressures (see Table S31). For resilience, clean water enforcement and regulation and all of the social resilience measures were included (see Table S32).

## Coastal Livelihoods and Economies

This goal focuses on avoiding the loss of ocean-dependent livelihoods and productive coastal economies while maximizing livelihood quality. We measure the status of this goal through two sub-goals: livelihoods (i.e., jobs and wages) and economies (i.e., revenues). Each goal is measured using sector-specific data from the National Ocean Economics Program (NOEP); sectors include: living resources, tourism and recreation, shipping and transport, marine related construction, and ship and boat building/repair. For each of these sub-components we use sector-specific multipliers derived from the NOEP data so that we assess both direct and indirect effects. We recognize that sectors and economic activity within a region can be influenced by activities outside the region (e.g., fish caught in Alaska could be brought to Washington for processing, or vice-versa), thus leading to an over- or under-estimate of economic benefits derived from marine ecosystems within the study region. As with any ecosystem study, defining boundaries for the ecosystem is an artificial operation, and linkages with external elements necessarily exist but are challenging to account for. We currently have insufficient information to make accurate estimates of this exogenous input into regional economies, and interpretations of results must take this into account.

Livelihood sub-goal: As was done in the global analysis, coastal livelihoods is measured by two equally weighted sub-components, the number of jobs (*j*), which is a proxy for livelihood quantity, and the per capita average annual wages (*g*), which is a proxy for job quality. For jobs we used a no-net loss reference point whereas for wages we used a spatial comparison. Therefore, the number of jobs is calculated by summing the total value in each *k* sector across all *n* sectors in the current year *c* relative to the value in a recent moving reference period, *r*, defined as 5 years prior to *c*, and average annual wages as the total value across all *n* sectors in the current year relative to the highest value across all years and regions (as a spatial reference point), such that:

$x_{LIV}=\frac{j'+g'}{2}$ (Eq. S20)

$j'=\frac{\left(\frac{\sum_{k=1}^{n} j_{c,k}}{\sum_{k=1}^{n} j_{r,k}}\right)}{\frac{M_{c}}{M_{r}}}$ (Eq. S20a)

where M is each State’s employment rate as a percent (M = 100 – unemployment) at current (c) and reference (r) time periods, and:

(Eq. S20b)

where W is each State’s average annual per capita wage at current (c) and reference (r) time periods.

Economies sub-goal: The coastal economies sub-goal is composed of a single component, revenue (*e*), measured in 2000 USD (to make them equivalent – the choice of year does not affect results). As was done for the livelihoods sub-goal, status is based on a no-net loss reference point. Therefore, status is calculated as revenue from each k sector in the current year *c* relative to revenue from a recent moving reference period, *r*, defined as 5 years prior to *c*, such that:

$x_{ECO}=\frac{\sum_{k=1}^{n} \frac{e_{c,k}}{e_{r,k}}}{\frac{E_{c}}{E_{r}}}$ (Eq. S21)

where E is each State’s annual total GDP at current (c) and reference (r) time periods.

The denominators in equations S20a, b and S21 represent adjustments for broader economic forces that may be affecting livelihoods and economies independent of changes in ocean health, using individual State-level metrics. As noted, jobs were adjusted by the overall State-level employment, wages were adjusted by the State’s average annual per capita wages, and revenue was adjusted by the State’s GDP. Absolute values for jobs and revenue were summed across counties and sectors and absolute values for wages were averaged for both current and reference periods before calculating relative values per region. For status we used 2009 as the current year (due to data constraints), such that the reference year was 2004.

**Table S10:** Sectors included in Livelihoods and Economies calculations:

| **Living Resources** | Fish Hatcheries and Aquaculture |
| --- | --- |
|  | Fishing |
|  | Seafood Markets |
|  | Seafood Processing |
| **Tourism and Recreation** | Amusement and Recreation Services |
|  | Boat Dealers |
|  | Eating and Drinking Places |
|  | Hotels and Lodging |
|  | Marinas |
|  | Recreational Vehicle Parks and Campsites |
|  | Scenic Water Tours |
|  | Sporting Goods Retailers |
|  | Zoos, Aquaria |
| **Shipping & Transport** | Deep Sea Freight |
|  | Marine Passenger |
|  | Marine Transportation Services |
|  | Dredge and Navigation Equipment |
|  | Warehousing |
| **Marine Related Construction** | Marine Related Construction |
| **Ship & Boat Building/Repair** | Ship and Boat Building and Repair |

Trend was calculated as the percentage change in score for 2004 and 2009 using a linear model across the individual sector values (aggregated across counties but not sectors) for the adjusted jobs, wages and revenues. We then calculate the average trend for jobs and wages across all sectors, weighted by the number of jobs in each sector in 2009, and the average trend for revenue across all sectors, weighted by the revenue in each sector in 2009. We then average the wages and jobs average slopes to get a trend value for coastal livelihoods, and use the weighted average slope in revenue for coastal economies*.* We included different pressures and resilience measures for each sector (see Tables S31 and S32 for a full breakdown of how these measures were applied). To calculate ecological pressures we took the average weight across all sectors for each pressure, and for social pressures we applied all measures included in the matrix evenly. Only the social resilience measures were used in the overall resilience score.

## Natural Products

The decision to exclude natural products from Index calculations was based on several factors. Most notably, there are no data available on local- or regional-scale harvest for most products, even though such harvest likely occurs at small scales throughout the region. Without harvest data, we cannot calculate current status scores or estimate sustainable harvest levels. The lack of harvest data suggests that natural products constitute a very small part of coastal economic activity. Seaweeds (i.e., kelp) represent the only natural product for which harvest data exist, but this harvest only occurred in Southern and Central California. Thus for Washington, Oregon and Northern California we have no known products that were harvested or data on harvest levels, and thus this goal drops out of the analysis for these sub-regions.

For kelp, harvest ceased in 2008 according to NMFS catch data. The peak in harvest of seaweeds in California was in 1975, with 171,597 tons of kelp landings reported. In the last year that harvest was recorded (2007) this value dropped to just 2% of this historical peak (just over 4000 tons of seaweeds landed in 2008). In a case where a product was overharvested to commercial collapse, a lack of harvest would be penalized in the Index and given a score of zero. Because kelp is still relatively abundant in California and lease options to harvest kelp still exist, we presume that kelp harvest ceased due to economic or social reasons. In either case, the decision to stop harvesting kelp suggests the natural product is no longer a valued part of the ‘health’ of the ocean ecosystem, and thus no longer a relevant component of the Ocean Health Index.

Given this situation, the two options for how to include this goal in the assessment were to give Southern and Central California sub-regions a zero for the natural products goal (lowering the overall Index score), while excluding it from the other sub-regions, or to have the goal drop out completely of the assessment. We felt it was much more reasonable to do the latter. If instead one feels that this goal should be given a score of zero for Southern and Central California, the resulting overall Index score would be 66 for Southern California (instead of the current score of 73) and 64 for Central California (instead of 71).

# Methods: Additional Analyses

Results for these analyses are presented in Fig. 4 and Tables 1 and 2 in the main text.

## Time Series of Status Scores

To evaluate how Index scores have changed over time, we calculated current status scores for each goal or sub-goal for which sufficient data existed. We were unable to calculate complete scores (current status plus likely future state) because insufficient data existed to allow for calculation of trend, pressures or resilience dimensions through time for any of the goals. To calculate the current status in times prior to the current year, we held the reference point the same and used previous years values as the present value for that year.

For the fisheries sub-goal, since the latest year of assessment varied across species (as recent as 2011 for some), we had to constrain the current year (i.e., the year of assessment for current status) to 2004 for all stocks to ensure all stocks had the same ‘current year’. Thus, the temporal assessment of fisheries does not span the period of time when MPAs were created throughout California state waters. In the future, when more stocks have been assessed in more recent years, it will be valuable to assess whether or not the potential impact of the MPAs on fisheries status of coastal stocks can be detected by this indicator.

For the lasting special places sub-goal, all land-based (coastal 1 mi) protected areas have recorded dates of creation of 2007 or later. This obviously is not correct, but without more accurate information, we could not assess the temporal change in land protected area extent. Thus, the time series for status of this goal represents only changes in marine protected areas.

##

## Sensitivity Analyses

***Weighting values***

The weights applied to each goal to calculate a single index score for each region are assumed to be equal, even though we know this does not likely represent the true values that individuals would place on each goal. However, it is difficult to derive the values a large group of individuals would place on each goal and it is likely that these values differ along many social and cultural axes. In order to attempt to quantify these values, a workshop was held in San Francisco, California, to elicit preferences that various local stakeholders have for the various goals included in the Ocean Health Index and the weights they would assign to the importance of each goal in their contribution to overall ocean health. We used two methods to elicit preferences based on the tradeoffs that would likely emerge from management decisions within the region. The full methodology for deriving these weights is presented elsewhere (Halpern et al. 2013). Results for the value sets derived at this stakeholder workshop of regional experts are shown in Table S11.

**Table S11:** Weighting schemes derived from stakeholder workshop and those meant to represent different potential value sets (Halpern et al. 2013).

| **Goal** |  |
| --- | --- |
| **Food Provision** | 0.086 |
| **Artisanal Opportunity** | 0.075 |
| **Natural Products** | 0.102 |
| **Carbon Storage** | 0.087 |
| **Coastal Protection** | 0.073 |
| **Coastal Livelihoods and Economies** | 0.053 |
| **Tourism and Recreation** | 0.068 |
| **Sense of Place** | 0.177 |
| **Clean Waters** | 0.205 |
| **Biodiversity** | 0.075 |

***Fisheries sub-goal Sensitivity Analysis***

To include data poor stocks in the assessment of the fisheries sub-goal we applied a score of 0.5 to the unassessed portion of the average catch. This default value was chosen for the following reasons. First, a global assessment has shown that B/B_MSY_ in data poor stocks is roughly 30% lower than assessed stocks (Costello et al., 2012), and in the U.S. west coast assessed stocks had an average B/ B_MSY_ score of 0.7. Second, data poor stocks on the west coast of the United States are managed under highly precautionary catch levels (a fraction of historically-stable catch (Restrepo et al. 1998)), suggesting a significantly lower F/F_MSY_ value for data poor than assessed stocks. In fact, NOAA technical guidance recommends that catch be set as low as 25% of historically stable catch in many data moderate and data poor situations (Restrepo et al. 1998). We assumed F/F_MSY_ was 0.5 for all data poor stocks, likely too high a value. Using these values in Eq. S1a, data poor stocks therefore receive a score of 0.5.

***Tourism and Recreation Sensitivity Analysis***

For the regional analysis of the Tourism and Recreation goal we used a temporal reference point to measure how successfully each sub-region was accomplishing this goal. Therefore, a perfect goal score was dependent on a reference point internal to each sub-region, that is, we measured the goal using no-net loss in participation within each sub-region as a reference point. In the previous global analysis (Halpern et al. 2012) international arrivals were used as a proxy for the value of tourism and recreation in each region, and instead of the temporal reference point used here, a spatial reference point was implemented of 110% of the highest observed value across all EEZs/reporting units. To assess how the regional results would have been altered by the selection of a spatial reference point rather than the temporal one implemented, we ran the goal model using 110% of the highest participation value observed in the U.S. west coast as the reference point (i.e. participation in California). Results for Oregon and Washington varied greatly with the change in reference point as participation in recreation was no longer compared to an internal previously observed level, but rather the level observed in California. This state has inherent advantages that promote increased recreational participation such as higher coastal temperature, more sandy beaches, and less storm action. In light of these inherent differences we selected the temporal reference point as a more informative representation of how well each sub-region was accomplishing the tourism and recreation goal.

***Artisanal Fishing Opportunity Sensitivity Analysis***

For the regional analysis we changed the global artisanal fishing opportunity model to make the best use of information and more closely describe the types of artisanal fishing opportunities pursued in the area (which are not necessarily driven by poverty, as the global model assumed) through measures of physical and economic access to the resource directly, as well as the state of resource management. At the global level, Halpern et al. 2012 assessed artisanal opportunities as a function of need (based on the level of poverty present in a region) and the effectiveness of small-scale fisheries management. To assess the impacts of our model changes we also ran the global version of the artisanal fishing opportunity model using regionally specific data and the addition of stock status information which was unavailable for the global scale analysis, but would ideally be included if such information were to become available. The 2012 global model estimates artisanal fishing opportunity as a function of stock status (S_AO_) and the unmet demand (D_U_) for such opportunity, i.e., the proportion of demand that is not satisfied by available opportunities:

*x_AO_* = (1 – *D_U_*) * *S_AO_*, (Eq. S22)

*D_U_* = (1 – *PPPpcGDP*) * (1 – *O_AO_*), (Eq. S23)

where O_AO_ represents the ‘Artisanal fishing: management effectiveness and opportunity’ score extracted from Figure S4 of Mora et al. 2008 for the U.S. west coast, i.e., the opportunity supplied; PPPpcGDP is the log-transformed GDP adjusted Purchasing Power Parity (PPP) per state for the current year (2010), rescaled from 0 to 1 using the maximum observed value as 1, which were obtained from the U.S. Dept. of Commerce, Bureau of Economic Analysis; and stock status information comes from the NOAA FSSI scores also used in the regional analysis of artisanal fishing opportunities.

***Mariculture Sub-goal Reference Point Sensitivity Analysis***

Due to the local cultural and economic significance of shellfish mariculture in the U.S. west coast we modified the global mariculture sub-goal reference point to more accurately reflect the local values and desired targets. For this model we chose an established Federal target for increases in the domestic production of seafood as we assumed this to be reflective of the regional goals for the production of seafood from mariculture. To test the impacts of our reference point selection we also ran the model for each state using two different spatial reference points (global maximum and U.S. national average observed bivalve production density), a temporal (no-net loss) reference point, and a reference point based on a production function (the maximum recommended per-farm density). Details on each alternative reference point tested are shown in Table S12 below.

International and national production density reference points were eliminated as possible options because the regional potential for production may not be comparable to what is possible elsewhere where local environmental characteristics may be different. The no-net loss perspective was excluded as a possible reference point because it is clear from the literature and local management plans (Dumbauld et al. 2009, Nash 2004, Puget Sound Water Quality Action Team 2000) that the goal for production of seafood is not simply to maintain current production values, but instead to increase production. Lastly, the production function model was excluded as a meaningful reference point because it is based on the production density of a single farm and does not account for the total production density feasible across all potential growing areas. As data are not available for the exact area currently dedicated to individual shellfish farms in each area we had to rely on total potential area in each bay, which likely greatly overestimates the area over which maximum production is actually possible.

**Table S12**. Description and definition of potential alternative mariculture sub-goal reference points

| **Reference Point Type** | **Description** | **Reference point** |
| --- | --- | --- |
| Spatial reference point 1 (China's production density) | Reference point is based on the maximum globally observed shellfish mariculture production density within 3nm of the coast for a single country (China). | 1.635 bivalve tons/ha within 3nm |
| Spatial reference point 2 (U.S. National production density) | Reference point is based on the U.S. national average shellfish mariculture density within 3nm of the coast | 0.020 bivalve tons/ha within 3nm |
| Temporal reference point (No net loss) | Reference point is based on no net loss in shellfish production for each state individually (each state is only compared to its own previous 2005 production values) | 1 - percent loss in state-wide production since 2005 |
| Production Function (Maximum recommended density) | Reference point is based on the maximum recommended density within a single farm (from Nash 2004) | 60 tons/ha |

## Scenario Analyses

We explored three different hypothetical but realistic management decisions to assess how the Index would reflect the consequences of those decisions. We used highly simplified assumptions in these scenarios that largely ignored ecological and social interactions and cascading effects, although the third scenario captures such cascading effects in a limited and simplified way. As such, the intent of these scenarios was not to predict the actual change in Index scores under these management actions, but instead to illustrate the response of the Index under these scenarios and how it could be used to explore system responses to different types of actions. In real decision-making scenarios, information or model outputs on the interactions and cascading effects mentioned above would be used to inform the scenario (e.g., using spatially explicit fisheries and population models to predict changes in fishing yield with and without MPAs). For each scenario we explain why we made the assumptions and where the values came from, again emphasizing that the scenarios are intended to be illustrative rather than prescriptive such that the exact values derived from the assumptions are not critical.

**Scenario 1-** What if 5 years ago land-based runoff had decreased by 25% due to more effective land-use regulations?

For this scenario we made two simple changes. First, we multiplied the pressures associated with land-based nutrients and pesticides (nutrient and organic pollution) by 75% in each pixel. This is equivalent to decreasing the total pressure value for each pollution layer for each sub-region by 25%. These modified pressure layers were then re-applied to the many goals for which they are relevant, as described above and detailed in Table S31. We also made the assumption that this decrease in pollution occurred because regulations were enacted that were implemented and effective, such that every goal that had land-based pollution pressures weighted 2 or 3 in Table S31 had the ‘clean water enforcement and regulation’ resilience measure changed to a score of 1.0 (Table S32).

***Basis for assumptions*:** Although few policy documents state explicit targets for reductions in land-based pollution, reducing this pollution is a widely stated objective. Concrete stated reduction numbers include ‘ensuring a 30% reduction in agricultural pollution runoff to the [San Francisco] Delta by 2012’ (Central Valley Regional Water Quality Board; <http://www.cacoastkeeper.org/document/report-to-legislature-on-delta-agricultural-pollution.pdf>) and ‘reduc[ing] wastewater flows 15% below 2000 levels by 2010’ (City of Santa Monica: <http://www.smgov.net/uploadedFiles/Departments/OSE/Categories/Sustainability/Sustainable-City-Plan.pdf>). We also spoke with Steve Weisberg, Executive Director of Southern California Coastal Water Research Project, who confirmed that 25% reductions were realistic targets for Southern California, suggesting they would be reasonable for the other sub-regions within the US West Coast.

**Scenario 2-** What if restoration efforts had increased wetland and sand dune habitat coverage by 10% above current levels?

For this scenario we simply increased the current extent of sand dunes and wetland habitats in each sub-region by 10%. The revised ‘present value’ scores for these habitats were then incorporated into status assessments for the habitat-based goals (carbon storage, coastal protection, and the habitats sub-goal of biodiversity). We did not change any pressures or resilience measures, even though such a change in habitat extent would almost certainly require management action that would boost resilience scores and decrease associated pressures scores. One could simulate these changes by making assumptions about which actions would need to be taken to achieve such restoration and which pressure layers would decrease in response to those actions.

***Basis for assumptions*:** Coastal habitat restoration is a widely stated objective in most coastal planning documents, in particular along the US West Coast. Examples of explicit restoration values include restoring ‘20% more eelgrass by 2020’ (Puget Sound Management Conference; <http://www.mypugetsound.net/index.php?option=com_docman&task=doc_view&gid=1597&Itemid=238>), ‘50% increase of estuarine habitats’ (Puget Sound Partnership Estuary Restoration; <http://www.psp.wa.gov/downloads/SOS2012/EstuaryRestoration_110112.pdf>) and ‘restoration of at least 15% of degraded ecosystems’ (Aichi Target 15; <http://www.cbd.int/doc/strategic-plan/2011-2020/Aichi-Targets-EN.pdf> ). Changes in the reference point for habitat-based goals would by definition affect how much of a change this scenario would represent within the Index; we have not explored this effect here as it was beyond the scope of this study, but it could easily be done.

**Scenario 3-** What if the Marine Life Protection Act (MLPA) process had not been undertaken and no MPAs added in California since 2007?

For this scenario we tried to simulate some simple cascading effects (through time) by running three increasingly complex versions of the scenario that are based on assumptions about how a management action might change a system through time.

***Iteration a:*** We first assumed that because the MLPA process did not occur, no additional MPAs were created in any of the California sub-regions later than 2007, but also that no additional ecological or social consequences occurred. As such, this first iteration of the scenario only affects the lasting special places sub-goal of the sense of place goal, which is measured in part by the amount of MPAs in the water, and the resilience dimension of other goals for which MPA coverage is relevant (see Table S32).

***Iteration b:*** Building on the first iteration, we then assumed trawling pressure would be higher than it currently is (with MPAs in place) and that some of the benefit to species from current MPAs would be lost. Within the Index framework, we translated these assumptions into a 5% increase in trawling pressure within each of the California sub-regions (a pressure layer for many of the goals), a resulting 5% decrease in the status of soft bottom habitats (which is measured via the proxy of trawling pressure), and a 5% decrease in the scores for the species sub-goal in the biodiversity goal.

***Iteration c:*** Finally, we assumed that along with increased trawling pressure, fishing pressure from other gear types would also be higher, but that these increased fishing pressures would also lead to higher (we assume sustainable) catch, and thus greater food provision from the fisheries sub-goal. We thus increased the food provision score for California by 5% (note that there is very little mariculture in California, so the food provision score is predominantly driven by the fisheries sub-goal), and that all other fishing pressures increase by 5% for all the goals that are affected by fishing pressure.

***Basis for assumptions*:** Iteration a makes the assumption that MPAs have no effect beyond their boundaries or to any aspect of the ocean ecosystem besides biodiversity conservation (or they have not manifested their effects yet), such that their absence only changes the condition of those particular locations. It is an unlikely assumption but serves as a useful baseline to then compare more complex potential interactions. Iteration b assumes that the MPAs reduce fishing pressure by keeping some trawl fisheries out of the protected locations and that this reduction is not compensated by displacement of trawling effort elsewhere in the area, such that an absence of MPAs would lead to higher than current levels of trawling. The exact amount of increased trawling that would occur is difficult to predict; we assumed a 5% increase to allow a small but significant increase to occur. For MPAs that primarily protect rocky habitats, this would be an overestimate, whereas for MPAs that protect soft sediment habitat, this would be a significant underestimate. In the final iteration (iteration c), we assumed that more than just trawl fisheries would move into the MPAs, but that these kinds of fishing tend to be more sustainable (we assumed fully sustainable for the sake of simplicity). This iteration, thus, not only assumes all fishing in the (former) MPA regions would be sustainable but also that MPAs have no benefit to fisheries outside their boundaries, i.e., that all increased fishing in the areas where MPAs currently are would be additional catch beyond what was possible with MPAs in place, or in other words that current MPAs provide no catch to fished areas viaspillover of fish from within MPAs. Other spatial dynamics of fisheries that could affect outcomes depending on where MPAs are located, such as nursery areas or stock aggregations, were also not taken into consideration in this simplified simulation as they were beyond the scope of the exercise.

# Specific Data Layers

## Alien Invasive Species

*Where used*: Pressure for several goals

*Scale:* Global analysis

*Description*: These data come from the global invasive species database (Molnar et al. 2008). This database reports number and type of alien species in each marine ecoregion, with species types categorized as invasive and harmful invasive species. For our purposes, total count of all invasive species was used. Six ecoregions were included in the U.S. west coast analysis: Northern California, Southern California Bight, Oregon, Washington, Vancouver Coast and Shelf, and Puget Trough/Georgia Basin. We intersected the ecoregion data with our reporting units to determine the proportion of each ecoregion that falls within each reporting unit and then assigned this percentage of invasive species from the ecoregion to the reporting unit. The sum of all invasive species within each reporting unit was then rescaled to the maximum global value. See Halpern et al. (2012) for further details.

## Clean Water Enforcement and Regulation-

*Where used*: Resilience measure for many goals

*Scale:* Updated regional data

*Description*: The Clean Water Enforcement and Regulation resilience measure is a composite measure of 3 different enforcement and compliance metrics utilized by the US EPA National Pollutant Discharge Elimination System (NPDES) permit program, which acts to control water pollution by regulating point sources that discharge pollutants into US waters. Yearly metrics measuring the percentage of inspector coverage at NPDES major facilities, the ratio of actual to expected discharge monitoring report submissions, and the percentage of discharge facilities with compliance violations were obtained from the US EPA Enforcement & Compliance History Online (ECHO) database (<http://www.epa-echo.gov/echo/dashboard/dashboard_all>) for California, Oregon and Washington. These values are reported on a 0-100% scale. We used the EPA established ‘National Goals’ for each metric as our reference resilience score. The national goal was 50% for inspector coverage, 95% for monitoring report submission, and 0% for compliance violations. For each of these three metrics we divided the observed value by its reference score (so that when it equals the reference it receives a perfect score of 1) and averaged them to come up with a resilience score for each state.

## Climate Change Resilience

*Where used*: Resilience for coastal protection, carbon storage, and biodiversity goals

*Scale:* Updated regional data

*Description*: This Resilience measure was based on the a U.S. state-level assessment by the Center for Climate and Energy Solutions (2012) that looks at the implementation of Climate Change related initiatives across 4 sectors (Climate Action, Energy Sector, Transportation, and Building Sector). To create a score for this layer each initiative present within a state was given a point, with a potential for 26 points total across all 4 sectors. Scores were then allocated as a percent of the total potential and rescaled between 0 and 1.

## Coastal Access Points

*Where used*: Status for artisanal fishing opportunity goal and lasting special places subgoal

*Scale:* Updated regional data

*Description*: Data on the location of coastal access points come from 3 separate sources for California, Oregon, and Washington. For California coastal access points, data come from the NOAA Environmental Sensitivity Index (ESI), which is a mapping effort to identify all coastal resources that may be at risk in the event of an oil spill including both biological and human resources. Data are available for California in four separate geodatabases by region for a different year in each sub-region — Southern California (2010), Central California (2006), Northern California (2008), and San Francisco Bay (1998). The following access point types were included in our analysis, as they provide public access to the marine environment: A2 (Access), BR (Boat Ramp), M (Marina), RF (Recreational Fishing), and S (Subsistence). Data for Oregon come from the Oregon Coastal Atlas Coastal Access Inventory which provides location and descriptive information for all public beach access points in coastal Oregon. All designated public access types were included in our analysis for Oregon. For Washington, data from the Washington Marine Shoreline Public Access Project were used to identify coastal access sites (See Table S13 for classes used for public access sites in Washington and full description). Using these data we identified the percentage of the entire coast that is within 1 mile of a coastal access point, with the target set at the entire coastal area being within 1 mile of a coastal access point.

**Table S13:** Public Access Class Codes and descriptions for Washington

| **CLASS CODE** | **CLASS DESCRIPTION** | **MORE DETAIL** |
| --- | --- | --- |
| **PUB1** | GOVERNMENT PARK | FEDERAL, STATE, COUNTY, CITY, OR OTHER GOVERNMENT OWNED PARK ACCESSIBLE TO ANYONE |
| **PUB2** | GOVERNMENT OWNED LAND WITH KNOWN PUBLIC ACCESS | FEDERAL, STATE, COUNTY, CITY, OR OTHER GOVERNMENT OWNED LAND (UPLANDS & TIDELANDS) ACCESSIBLE TO ANYONE |
| **PUB3** | GOVERNMENT OWNED TIDELANDS WITH KNOWN PUBLIC ACCESS FROM LAND | GOVERNMENT OWNED TIDELANDS WITH KNOWN PUBLIC ACCESS. ACCESSIBLE FROM PUBLIC ACCESS POINT OR FROM AN ADJACENT PUBLIC BEACH. UPLANDS ARE PRIVATE OR NONACCESSIBLE GOVERNMENT OWNED LAND. |
| **PUBRD** | PUBLIC ROAD END | ROAD END OWNED BY A GOVERNMENT ENTITY AND OPEN TO ANYONE IN THE PUBLIC |
| **PUBBOAT** | PUBLIC BOAT LAUNCH | BOAT LAUNCH OWNED BY A GOVERNMENT ENTITY AND OPEN TO ANYONE IN THE PUBLIC |
| **PUBDOCK** | PUBLIC DOCK | DOCK OWNED BY A GOVERNMENT ENTITY AND OPEN TO ANYONE IN THE PUBLIC |
| **PUBPIER** | PUBLIC PIER | PIER OWNED BY A GOVERNMENT ENTITY AND OPEN TO ANYONE IN THE PUBLIC |
| **PUBMARINA** | PUBLIC MARINA | PROPERTY OWNED BY A GOVERNMENT ENTITY AND TYPICALLY OPEN TO ANYONE IN THE PUBLIC |
| **PUBFERRY** | PUBLIC FERRY TERMINAL | FERRY TERMINAL OWNED BY A GOVERNMENT ENTITY AND OPEN TO ANYONE IN THE PUBLIC |
| **BRIDGE** | BRIDGE ON A PUBLIC ROAD/HIGHWAY | BRIDGE OWNED BY A GOVERNMENT ENTITY (USUALLY DOT) AND OPEN TO ANYONE IN THE PUBLIC. MANY BRIDGES (MUD BAY BRIDGE IN OLYMPIA, STEAMBOAT ISLAND BRIDGE) ARE POPULAR WITH FISHERMEN AND OFFER ACCESS TO THE SHORE. |
| **OPW1** | GOVERNMENT OWNED PARK ACCESSIBLE ONLY BY PERSONAL WATERCRAFT | PARK OWNED BY THE GOVERNMENT ACCESSIBLE TO THE PUBLIC ONLY BY PERSONAL WATERCRAFT BECAUSE IT IS ON AN ISLAND THAT IS NOT SERVICED BY THE STATE FERRY OR UPLANDS ARE PRIVATE OR NON-ACCESSIBLE. |
| **OPW2** | GOVERNMENT OWNED LAND ACCESSIBLE ONLY BY PERSONAL WATERCRAFT | LAND OWNED BY THE GOVERNMENT ACCESSIBLE TO THE PUBLIC ONLY BY PERSONAL WATERCRAFT BECAUSE IT IS ON AN ISLAND THAT IS NOT SERVICED BY THE STATE FERRY OR UPLANDS ARE PRIVATE OR NON-ACCESSIBLE. |
| **OPW3** | GOVERNMENT OWNED TIDELANDSACCESSIBLE ONLY BY PERSONAL WATERCRAFT | TIDELANDS OWNED BY THE GOVERNMENT ACCESSIBLE TO THE PUBLIC ONLY BY PERSONAL WATERCRAFT BECAUSE IT IS ON AN ISLAND THAT IS NOT SERVICED BY THE STATE FERRY OR UPLANDS ARE PRIVATE OR NON-ACCESSIBLE. |

## Coastal Counties

*Where used*: used with other data layers in a variety of dimensions for all goals

*Scale:* Updated regional data

*Description*: Coastal counties were defined as any county within California, Oregon, or Washington that had any marine or bay coastline (Table S14). To identify these counties first we extract a ~10m resolution coastline vector from county data (NOAA 2000; ESRI 2010) for counties immediately adjacent to the Pacific Ocean and inland waters within our region of interest. To calculate county membership for the coastline, we segmented the coastline into simple lines with no more than 2 vertices (and thus the longest line segment is the longest straight-line distance on the coastline), and then computed which county was nearest (in straight-line distance up to a maximum of 10 km) to the center of that line segment. We then dissolved the coastline line segment into a single polyline vector file with one vector per county (where the membership key is a 5 digit state/county Federal Information Processing Standard (FIPS) code).

Table S14 – Counties within study area, including both coastal and inland locations.

| **State** | **Region Code** | **County** | **Coastal?** |
| --- | --- | --- | --- |
| California | Northern California  CA1 | Del Norte | y |
|  |  | Humboldt | y |
|  |  | Mendocino | y |
|  |  | Sonoma | y |
|  | Central California  CA2 | Alameda | y |
|  |  | Contra Costa | y |
|  |  | Marin | y |
|  |  | Monterey | y |
|  |  | Napa | y |
|  |  | Sacramento | inland |
|  |  | San Francisco | y |
|  |  | San Joaquin | inland |
|  |  | San Luis Obispo | y |
|  |  | San Mateo | y |
|  |  | Santa Clara | y |
|  |  | Santa Cruz | y |
|  |  | Solano | y |
|  |  | Yolo | inland |
|  | Southern California  CA3 | Los Angeles | y |
|  |  | Orange | y |
|  |  | San Diego | y |
|  |  | Santa Barbara | y |
|  |  | Ventura | y |
| Oregon | OR | Clatsop | y |
|  |  | Coos | y |
|  |  | Curry | y |
|  |  | Douglas | y |
|  |  | Lane | y |
|  |  | Lincoln | y |
|  |  | Tillamook | y |
| Washington | WA | Clallam | y |
|  |  | Grays Harbor | y |
|  |  | Island | y |
|  |  | Jefferson | y |
|  |  | King | y |
|  |  | Kitsap | y |
|  |  | Mason | y |
|  |  | Pacific | y |
|  |  | Pierce | y |
|  |  | San Juan | y |
|  |  | Skagit | y |
|  |  | Snohomish | y |
|  |  | Thurston | y |
|  |  | Wahkiakum | y |
|  |  | Whatcom | y |

## Coastal Land and Ocean Area

*Where used*: used with other data layers in a variety of dimensions for all goals

*Scale:* Updated regional data

*Description*: To compute a coastal area per county adjacent to the coastline ("coastal pixels"), we first extract a ~10m resolution coastline vector (NOAA 2000) from county data for counties immediately adjacent to the Pacific Ocean and inland waters within our region of interest. We then computed a 1000m buffer from the coastline polyline vector. Then, to merge overlapping buffers with competing county membership, we rasterized the buffer at 10m resolution using the county membership as the cell value and specifying that counties with larger areas win ties. Finally, we vectorized the data using polygon simplification to one coastal area polygon per county. The result is 12,970 km^2^ of coastal area across 41 counties in our study area.

## Coastal population

*Where used*: used with other data layers in a variety of dimensions for all goals

*Scale:* Updated regional data

*Description*: The data come from the NOAA State of the Coast Report, Population Living in Coastal Watershed Counties, 1970-2030. For the most current year (2011), U.S. Census Bureau data are applied for each county included in NOAA’s Coastal Watershed Counties grouping (U.S. Census Bureau 2010).

## Coastline and Coastal Zone Area

*Where used*: used with other data layers in a variety of dimensions for all goals

*Scale:* Global analysis

*Description*: We projected the global land-sea model into the US west coast projection, and then calculated the land, inland, and offshore coastal zone buffers for each of our sub-regions and the entire region. See global analysis (Halpern et al. 2012) for full description.

## Ecological integrity

*Where used*: Resilience for food provision, sense of place, and biodiversity

*Scale:* Global analyses

*Description*: This layer is a slight modification of the marine species layer described below. It is the weighted sum of assessed species, but with weights slightly modified from Table S21, such that: EX = 0.0, CR = 0.2, EN = 0.5, VU = 0.7, NT = 0.9, and LC = 0.99. We then calculated the spatial average of these per-pixel scores based on a 3 nm buffer for goals that are primarily coastal and for the whole EEZ for goals that derived from all ocean waters (see Table S32).

## EPA Beach Closure data

*Where used*: Status and trend for clean waters goal, pressure for many goals

*Scale:* Updated regional data

*Description*: Beach closure data come from the EPA Annual Beach Notification Summaries (US EPA 2011) and give information on the percent of beach days where water samples exceed national pathogen standards by region (these are county level data that we aggregated to the selected regions in this study using the mean value). There were some gaps in the reporting so in California and Washington for 2006 we use state-level data, and for 2008 we use the average value in each county across 2006-2010. We then rescale these data to the maximum exceedence value (0.37) and a minimum of 0.0. The status score is then calculated as 1 – x per state, where x is the rescaled exceedence value for each region for the most recent year of data (2010).

## FDA Action Levels

*Where used*: Status and trend for clean waters goal, pressure for many goals

*Scale:* Updated regional data

*Description*: The US Food and Drug Administration (FDA) establishes action levels for poisonous or deleterious substances in food that represent limits at or above which the FDA will take legal action to remove food products from the market. For shellfish the levels for 11 contaminant categories are used by NOAA to establish a “bad” and “OK” threshold for contamination in local waters for the NOAA State of the Coast Report (2011). These thresholds are used in conjunction with the MusselWatch data to score the Chemicals component of the Clean Waters goal.

**Table S15.** MusselWatch NOAA State of the Coast Chemical Threshold Values-

| **Contaminant** | **ppm (Good)** | **ppm (OK)** | **ppm (Above FDA Action Level- Bad)** |
| --- | --- | --- | --- |
| **Arsenic** | 0 | 43 | 86 |
| **Cadmium** | 0 | 2 | 4 |
| **Chlordane** | 0 | 0.1 | 0.3 |
| **Chromium** | 0 | 6.5 | 13 |
| **DDT** | 0 | 2.5 | 5 |
| **Dieldrin** | 0 | 0.1 | 0.3 |
| **Lead** | 0 | 0.8 | 1.7 |
| **Mercury** | 0 | 0.5 | 1 |
| **Mirex** | 0 | 0.1 | 0.1 |
| **Nickel** | 0 | 44 | 80 |
| **PCB** | 0 | 1 | 2 |

## Fisheries Catch Totals

*Where used*: Status and trend for fisheries sub-goal, aggregation of sub-goals for food provision score

*Scale:* Updated regional data

*Description*: Data for wild-caught fish harvest weight by species in each region come from NOAA National Marine Fisheries Service (NMFS 2012). For the fisheries sub-goal the mean catch over the time series for each species was used to weight the contribution of each B/B_MSY_ and F/F_MSY_ derived score to the overall sub-goal score. The sum of all catch across species in year 2009 was used when combining the two sub-goals (mariculture and fisheries) to weight the contribution of wild-caught fisheries to the overall food provision goal score.

## Fishing Pressure

*Where used*: Pressure for many goals

*Scale:* Updated regional data

*Description*: For California, Oregon, and Washington we use NMFS catch data by species to determine the total amount of fish caught in each year (NMFS 2012). In order to separate the catch into our three California sub-regions we used data provided from the USGS compilation database “Pacific Coast Fisheries GIS Resource Database” (USGS 2012). The California Commercial Fisheries Data (1972-2009) are collected and provided by the California Department of Fish and Game's Marine Fisheries Statistical Unit (USGS 2012) and report catch per designated spatial block in California waters. These values were used to proportionally distribute NMFS catch data into the three California sub-regions by species in order to compare scores across all sub-regions. We then used the same categorical breakdown of species by gear types from Halpern et al. (2009) to assign a predominant gear type to each species caught throughout the time series for Oregon and Washington, and used the gear type designation to classify different gear types into our categories of fishing pressures. Finally, we summed metric tonnes across all catch within each fishing pressure category per year and sub-region, and used 110% of the maximum recorded tonnage as the maximum value for rescaling purposes across all sub-regions.

## Gas Price

*Where used*: Status for artisanal fishing opportunity goal and pressure and resilience for several goals

*Scale:* Updated Regional Data

*Description*: Average yearly gas (diesel) price data per state come from the U.S Energy Information Administration (<http://www.eia.gov/oil_gas/petroleum/data_publications/wrgp/mogas_history.html>). For our purposes we used the trend in the “All Grade, All Formulations Retail Gasoline Prices”, given in average dollars per gallon for each state, divided by the trend in average yearly wages within each state as a measure of change in how much salary individuals in each state were having to dedicate to purchasing gasoline over time. All values were adjusted to year 2000 U.S. dollar values and we used a ‘no-net gain’ reference point, using a 5-year window to compare the change in economic burden of gas price over time.

## Gas Price Solution Score

*Where used*: Resilience measure for fisheries sub-goal and tourism and recreation, artisanal fishing opportunities, and coastal livelihoods and economies goals

*Scale:* Updated Regional Data

*Description*: Gas Price solution scores were derived from the NRDC report “Fighting Oil Addiction: Ranking States’ Gasoline Price Vulnerability and Solutions for Change” (NRDC 2011). This report scores and ranks states based on how well they are diversifying their energy options and moving away from oil dependence as well as how vulnerable each state is to oil price increases. The scores reward states that are adopting significant measures to promote clean vehicles, clean fuels, and smart growth, based on 8 measures:

1. Vehicle GHG Emission Standards
2. State Fleet Efficiency
3. Low-Carbon Fuel Standard
4. Incentives for Electric Vehicles and Charging Infrastructure
5. Vehicle-Miles Traveled Reduction Target
6. Telecommuting Policy
7. Idling Restrictions
8. Growth Management Policies

The report describes how each state was scored as: “In order to calculate a solutions ranking of the 50 states, NRDC started by assigning a value of either a quarter point, half point, one point, or two points to each action in the table that a state currently takes. Actions with a bigger impact on oil dependence received more points:

- *2 points:* Low-carbon fuel standard in place or actively under development
- *1 point****:*** Vehicle GHG emission standards; signed on to low-carbon fuel standard MOU; vehicle-miles traveled reduction target codified or being implemented; smart-growth/growth management policies
- *1/2 point:* Incentives for electric vehicles and charging infrastructure; state fleet efficiency requirements; vehicle miles traveled reduction targets in the process of being established; idling restrictions; telecommuting policies
- *1/4 point:* Unfunded incentives for electric vehicles and charging infrastructure; strong telecommuting policy, but only for state employees

See the full report for a list of source date for each indicator. We used the state-level scores reported by NRCD in Table 4 and normalized them by the highest scoring state in the nation (California) to derive a gas price solution score between 0-1. This score then represents how resilient the state is to potential gas price increases.

## Genetic escapes

*Where used*: Pressure for several goals

*Scale:* Global analyses

*Description*: This layer represents the potential for harmful genetic escapement of cultivated species based on whether they are native or introduced. Data come from the Mariculture Sustainability Index (MSI; Molnar et al. 2008). In the MSI, native species receive the highest score (10), while foreign and introduced species receive the lowest (1) on the premise of potential impacts to local biodiversity if these species were to escape and mate with wild populations. The MSI reports data for 359 country-species combinations (with 60 countries and 86 species represented). The US specific score for mussels and oysters, and the average global score from clams were used for the entire study range. All scores were then rescaled from 0 to 1, using the maximum raw score of 10 and minimum of 1.

## Grading the States Report (Governance)

*Where used*: Pressure and resilience for all goals

*Scale:* Updated regional data

*Description*: The Grading the States Index is compiled by The Pew Charitable Trusts’ Government Performance Project (Barrett and Greene 2008) to measure how well states manage employees, financial details and budgets, information and infrastructure as an overall assessment of the quality of management in each state. These indicators were chosen as a way to reflect the potential for each state in the U.S. to deliver effective policy decisions and practices. Scores were generated based on interviews and surveys of state-level managers and opinion leaders in 2008. Each state is graded from A+ to F-. We took these values and transformed them to numerical values from 1 to 15 and then rescaled from 0 to 1 based on the highest potential score (15).

## Habitat Destruction, Intertidal Construction

*Where used*: Pressure for several goals

*Scale:* Updated regional data

*Description*: These data come from Halpern et al. (2009) and are based on the presence of coastal engineered structures such as riprap, seawalls, jetties, and piers. This layer was derived from the NOAA Environmental Sensitivity Index (ESI) and included codes 1B, 6B, and 8C.

## Habitat Destruction, Intertidal Trampling

*Where used*: Pressure for several goals

*Scale:* Updated regional data

*Description*: To estimate the impact of direct human trampling we used a model developed by Halpern et al. (2009) that utilized California actual beach attendance data to develop a predictive model of beach visitation across all regions included in the U.S. west coast. Predicted beach visitation values were used to score this pressure using 110% of the maximum visitation value recorded as the maximum pressure score, and zero visitations as the minimum. These values were then averaged across our sub-regions.

## Habitat Destruction, subtidal soft-bottom trawling

*Where used*: Pressure for many goals, status for soft-bottom habitats in the biodiversity goal

*Scale:* Updated regional data

*Description*: For California, Oregon, and Washington we use the data provided from the USGS compilation database “Pacific Coast Fisheries GIS Resource Database” (USGS 2012). For California the California Commercial Fisheries Data (1972-2009) were provided by the California Department of Fish and Game's Marine Fisheries Statistical Unit (USGS 2012). For Oregon and Washington trawl data come from Oregon Department of Fish Wildlife and the Washington Department of Fish and Wildlife respectively (USGS 2012). Pounds of all species caught using trawling gears were aggregated within each state for each year in the time series. These values were then applied equally across soft-bottom habitats (any depth) based on data from Halpern et al. (2009). 110% of the maximum catch per area across all regions was used as the maximum pressure value, with a target reference point of zero trawling in any area.

## Iconic species

*Where used*: Status and trend for iconic species sub-goal

*Scale:* Updated regional data

*Description*: The list of iconic species (see Table S16) was developed by regional experts both internal and external to the project. An exhaustive list of potential species that could be considered of high aesthetic value, associated with traditional activities such as fishing, hunting or commerce, or of local ethnic or religious significance to the people of California, Oregon, and Washington was developed and then narrowed based both on internal discussion and data availability from the NatureServe database. Details for the status and trend of these species are described below in the NatureServe data layer description.

**Table S16:** List of regional iconic species

| **Species common name *(scientific name)*** |
| --- |
| Bald Eagle (*Haliaeetus leucocephalus*) |
| Blue Whale (*Balaenoptera musculus*) |
| Bocaccio (*Sebastes paucispinis*) |
| Brown Pelican (*Pelecanus occidentalis*) |
| California Sea Lions (*Zalophus californianus*) |
| Delta Smelt (*Hypomesus transpacificus*) |
| Gray Whale (*Eschrichtius robustus*) |
| Great Blue Heron (*Ardea herodias*) |
| Abalone (*Haliotis* spp.) |
| Humpback Whale (*Megaptera novaeangliae*) |
| Northern Sea Otters (*Enhydra lutris kenyoni*) |
| Orca (*Orcinus orca*) |
| Osprey (*Pandion haliaetus*) |
| Salmon spp. (*Oncorhynchus* spp.) |
| Southern Sea Otters (*Enhydra lutris nereis*) |
| Steelhead (*Onchorynchus mykiss*) |
| Stellar Sea Lions (Eastern pop.) (*Eumetopias jubatus*) |

## Lenfest Report: An Economic, Legal and Institutional Assessment of Enforcement and Compliance in Federally Managed U.S. Commercial Fisheries

*Where used*: Resilience for food provision, artisanal fishing opportunity, sense of place, and biodiversity goals

*Scale:* Updated regional data

*Description*: This study conducted by the Lenfest Ocean Program is aimed at analyzing and quantifying the state of enforcement and compliance within U.S. Federally managed fisheries (www.lenfestocean.org). The study was national in scope, but was limited to case study regions and fisheries. For the Pacific coast the Pacific groundfish trawl fishery was the focus of the study. Included in this research were “(1) an analysis of national and regional fishery enforcement statistics; (2) mail and on-line surveys of fishermen, fishery enforcement staff, and fishery managers and scientists; and (3) interviews with fishermen, fishery enforcement staff, and fishery managers. The statistical results and survey and interview responses were compared with the results of previous studies to corroborate findings and identify trends and changes”. The Lenfest group developed a 58 question survey that was sent out to fishermen, regulators, enforcement staff and researchers in the Northeast, Gulf of Mexico and along the Pacific coast of the United States. For our purposes we broke the survey questions down into ‘Enforcement’ and ‘Compliance’ categories and used the respondents % agreement to score each question. Scores were then aggregated within each category to derive an average ‘Enforcement’ and an average ‘Compliance’ score and were rescaled to between 0-100. These scores were then averaged to come up with a single score for the Pacific coast.

## Mariculture Sustainability Index (MSI) Scores

*Where used*: Status and trend for mariculture sub-goal, fisheries goal

*Scale:* Global analysis

*Description*: For the sustainability measure included in the status measure for the mariculture sub-goal we used three separate indicators from the MSI: “fishmeal use”, “waste treatment”, and “seed and larvae origin”. These three indicators were selected because they are the only internal mariculture-driven stressors with the potential to affect the long term sustainability of mariculture production itself. The MSI reports data for 359 country-species combinations (with 60 countries and 86 species represented) for each assessment criterion. For mussels and oysters US specific scores were used, for clams we had to use the average of all countries for which that species was assessed because it was not included in the US specific assessment. Each species and each assessment criterion was aggregated and averaged based on the proportion of the landings that each locally-harvested shellfish species contributed to the overall catch in each region in the current year. All regional average scores were then rescaled from 0 to 1 using the maximum possible raw MSI score of 10 and minimum of 1, and then weighted equally to come up with a composite sustainability component of the status score. See Table S17 for the MSI scores applied to the shellfish species harvested in the region.

**Table S17:** Sustainability scores from the MSI for harvested shellfish species

| **Species** | **Fishmeal Use** | **Seed and Larvae Origin** | **Waste Treatment** | **Sustainability Score (S_k_)** |
| --- | --- | --- | --- | --- |
| **Clam** | 10 | 4.5 | 9.5 | **0.8** |
| **Mussel** | 10 | 7.25 | 9.75 | **0.93** |
| **Oyster** | 10 | 8 | 10 | **0.9** |

## Marine and Coastal Recreation Participation

*Where used*: Status and trend for tourism and recreation goal

*Scale:* Updated regional data

*Description*: Recreation participation data come from the National Survey on Recreation and the Environment (NSRE) which records participation in 19 coastal and marine recreational activities nationally (Leeworthy et al. 2005; See Table S9 for full list of recreational activities). This survey has been conducted 8 times nationally since 1960. The most recent data available for coastal and marine specific activities is from 2000, however Leeworthy et al. (2005) produced a report entitled “Projected Participation in Marine Recreation: 2005 & 2010” in which they forecast participation rates by activity based on correlated socioeconomic changes using logit equations and projected census data for 2005 and 2010 (see Table S9). We used these nationally applied logit equations and re-calibrated them to actual participation values recorded by the 2000 NSRE survey for each state. We then re-ran the equations using actual 2010 US Census data for the predicting variables to derive a participation rate per 100 state residents for all 19 activities (U.S. Census Bureau 2000, 2010).

## Marine Jobs, Wages, and Revenue

*Where used*: Status and trend for livelihoods and economies goal

*Scale:* Updated regional data

*Description*: These data come from the National Ocean Economic Program (NOEP) for the Ocean Economy (NOEP 2012). Data are currently available for 1990-2009 for six distinct ocean economy sectors, which are comprised of 22 different sub-sectors (see Table S18 for a full list of sectors and associated sub-sectors). NOEP defines the Ocean Economy as the economic activity which indirectly or directly uses the ocean as an input. This is in contrast to the broader category of coastal economy which includes all activity taking place in coastal areas. Data are available per sector for number of establishments, wage and salary employment (number of jobs), total wages, and Gross Domestic Product (GDP). For our analysis we used the wage and salary employment (jobs), wages (wages), and GDP (revenue) data. All economic data were converted into year 2000 dollar values. Values for wages were given as total wages for each sector; we converted these values to average wages per sector by dividing by the number of jobs in that sector in the same year based on the NOEP jobs data. NOEP-derived multiplier values were used on a per-sector, per-state, and per-year basis. For all three Pacific States the multipliers for 2008 and 2009 were not included in the data so the multiplier values were used from the next most recent year available. At the county level some data are considered proprietary, and when this occurred we filled these values with the state average in the same sector and year. Where there were gaps due to proprietary data at the whole state level for a given sector, we gap-filled using fitted values from a linear model as long as there were 3 data points. Where there were fewer than 3 data points, we assumed that sector was not present and filled the gap with a zero value. For the Living Resources sector we use the aggregate sector information at the state and county level for the status calculation. For the sector diversity calculation we use the disaggregated sector information, but calculate a single state-level score for California and apply it to all of the sub-regions in California.

**Table S18:** Sectors included in National Ocean Economic Program (NOEP) for the Ocean Economy:

| **Living Resources** | Fish Hatcheries and Aquaculture |
| --- | --- |
|  | Fishing |
|  | Seafood Markets |
|  | Seafood Processing |
| **Tourism and Recreation** | Amusement and Recreation Services |
|  | Boat Dealers |
|  | Eating and Drinking Places |
|  | Hotels and Lodging |
|  | Marinas |
|  | Recreational Vehicle Parks and Campsites |
|  | Scenic Water Tours |
|  | Sporting Goods Retailers |
|  | Zoos, Aquaria |
| **Shipping & Transport** | Deep Sea Freight |
|  | Marine Passenger |
|  | Marine Transportation Services |
|  | Dredge and Navigation Equipment |
|  | Warehousing |
| **Marine Related Construction** | Marine Related Construction |
| **Ship & Boat Building/Repair** | Ship and Boat Building and Repair |

## Marine Protected Areas

*Where used*: Status and trend for lasting special places sub-goal, resilience measure for many goals

*Scale:* Updated regional data

*Description*: Marine protected area information comes from NOAA’s Marine Protected Areas Inventory (National Marine Protected Areas Center 2012). This geospatial database contains comprehensive information on over 1700 sites within the US and includes information on year of designation and protection status. These data were used to determine the total area covered by marine protected areas within two regions: nearshore (0-3 nm buffer for each sub-region) and offshore (3-200nm). When used for the lasting special places goal, these two regions were treated separately; when used for resilience measures, a single score for the entire region (0-200nm) was used.

## Marine species

*Where used*: Status and trend for species sub-goal of biodiversity goal; ecological integrity resilience measure for several goals

*Scale:* Global analysis

*Description*: For status and trend, marine species listed within the IUCN Red List are used for the U.S. west coast region marine species list. Globally there are 2377 IUCN marine species for which distribution maps exist (Carpenter et al. 2008; Collette et al. 2011; IUCN 2011; Knapp et al.; Polidoro et al. 2010; Schipper et al. 2008; Short et al. 2011); however, only 140 of these species occur within the region so only the species status assessments for these species were included in the sub-goal score. Globally the main taxonomic groups included were: habitat-forming corals, mangroves, seagrasses, sea snakes, marine mammals, seabirds, marine turtles, angelfish, butterflyfish, groupers, wrasses, parrotfish, hagfish, and tuna and billfishes (see also Table S19). Because many of these taxonomic groups occur more often in tropical regions of the world, the species assessment available for the U.S. west coast remain limited. To augment these assessments, supplemental species risk assessments come from Davies and Baum (2012; see Supplemental Marine Species data layer description for full details on these species).

**Table S19.** Species from IUCN assessments included in the Species sub-goal of the Biodiversity goal

| **IUCN assessed species found in U.S. west coast waters** | | | |
| --- | --- | --- | --- |
| Acanthocybium solandri | Epinephelus labriformis | Mustelus californicus | Rhinochimaera pacifica |
| Acipenser medirostris | Erignathus barbatus | Mustelus henlei | Rhinoptera steindachneri |
| Acipenser transmontanus | Eschrichtius robustus | Mustelus lunulatus | Ruppia maritima |
| Alopias vulpinus | Etmopterus bigelowi | Mycteroperca jordani | Sarda chiliensis lineolata |
| Amblyraja hyperborea | Etmopterus lucifer | Mycteroperca xenarcha | Sarda orientalis |
| Arctocephalus townsendi | Etmopterus pusillus | Myliobatis californica | Scomber australasicus |
| Balaenoptera acutorostrata | Eumetopias jubatus | Myxine circifrons | Scomber japonicus |
| Balaenoptera borealis | Euprotomicrus bispinatus | Myxine hubbsi | Scomberomorus concolor |
| Balaenoptera musculus | Galeocerdo cuvier | Negaprion brevirostris | Sebastes paucispinis |
| Balaenoptera physalus | Galeorhinus galeus | Odontaspis ferox | Sebastolobus alascanus |
| Bathyraja aleutica | Grampus griseus | Oncorhynchus nerka | Semicossyphus pulcher |
| Bathyraja parmifera | Halichoeres semicinctus | Oxyjulis californica | Serranus huascarii |
| Bathyraja trachura | Harriotta raleighana | Paralabrax maculatofasciatus | Sphoeroides pachygaster |
| Callorhinchus callorynchus | Hemanthias peruanus | Paralabrax nebulifer | Sphyrna lewini |
| Callorhinus ursinus | Hemanthias signifer | Peponocephala electra | Sphyrna tiburo |
| Carcharhinus brachyurus | Hexanchus griseus | Phoca vitulina | Sphyrna zygaena |
| Carcharhinus falciformis | Hexatrygon bickelli | Phocoena phocoena | Squalus acanthias |
| Carcharhinus galapagensis | Hippocampus ingens | Phocoenoides dalli | Squatina californica |
| Carcharhinus limbatus | Hippocampus trimaculatus | Phyllospadix scouleri | Stenella coeruleoalba |
| Carcharhinus longimanus | Hydrolagus colliei | Phyllospadix serrulatus | Steno bredanensis |
| Carcharhinus obscurus | Hyporthodus acanthistius | Phyllospadix torreyi | Stereolepis gigas |
| Carcharhinus plumbeus | Isistius brasiliensis | Physeter macrocephalus | Thunnus alalunga |
| Carcharodon carcharias | Isurus oxyrinchus | Platyrhinoidis triseriata | Thunnus albacares |
| Caretta caretta | Isurus paucus | Pomacanthus zonipectus | Thunnus obesus |
| Cetorhinus maximus | Katsuwonus pelamis | Prionace glauca | Thunnus orientalis |
| Chaetodon humeralis | Lagenorhynchus obliquidens | Prognathodes falcifer | Torpedo californica |
| Chlamydoselachus anguineus | Lamna ditropis | Pronotogrammus multifasciatus | Tursiops truncatus |
| Coryphaena equiselis | Lampetra camtschatica | Pseudocarcharias kamoharai | Urobatis halleri |
| Dalatias licha | Liopropoma fasciatum | Pteroplatytrygon violacea | Xiphias gladius |
| Delphinus delphis | Lissodelphis borealis | Pungitius pungitius | Zalophus californianus |
| Dermatolepis dermatolepis | Megaptera novaeangliae | Raja binoculata | Ziphius cavirostris |
| Dermochelys coriacea | Mirounga angustirostris | Raja rhina | Zostera asiatica |
| Discopyge tschudii | Mobula japanica | Raja stellulata | Zostera japonica |
| Echinorhinus cookei | Mobula thurstoni | Rhincodon typus | Zostera marina |
| Enhydra lutris | Mugil cephalus | Rhinobatos productus | Zostera pacifica |

## NatureServe Species Assessments

*Where used*: Status and trend for iconic species sub-goal

*Scale:* Updated regional data

*Description*: NatureServe provides the conservation status for species at both global and regional scales using 6 categories: Secure, Apparently Secure, Vulnerable, Imperiled, Critically Imperiled, Presumed/Possibly Extinct. The NatureServe database is specific to the US and Canada and is updated and refined regularly based on information from natural heritage programs across the region. Status for each species is assessed at three distinct geographic scales: Global, National, and State/Province. Where available we use the State/Province status assessment; when that is unknown or unavailable we use the national status, and finally the global status if all other scales are unavailable. NatureServe utilizes information from field surveys, monitoring activities, expert consultation and scientific publications to assess all species. The NatureServe risk categories have been shown to correlate well with IUCN categories at the global scale (Goodenough 2012; Keith et al. 2004; Mehlman et al. 2004; Regan et al. 2004). Thus we scored the status categories in the same manner as was done with the IUCN categories in the global assessment (also used here in the species sub-goal for biodiveristy), as described in Butchart et al. (see Table 20 for categories and ranks and Table S21 for how they map to IUCN risk categories).

**Table S20:** NatureServe ranks, definitions, and criteria

| **Rank** | **Definition** | **Criteria** |
| --- | --- | --- |
| **X** | **Presumed Extinct** | Not located despite intensive searches and virtually no likelihood of rediscovery. |
| **1** | **Critically Imperiled** | At very high risk of extinction due to extreme rarity (often 5 or fewer populations), very steep declines, or other factors. |
| **2** | **Imperiled** | At high risk of extinction or elimination due to very restricted range, very few populations, steep declines, or other factors. |
| **3** | **Vulnerable** | At moderate risk of extinction or elimination due to a restricted range, relatively few populations, recent and widespread declines, or other factors. |
| **4** | **Apparently Secure** | Uncommon but not rare; some cause for long-term concern due to declines or other factors. |
| **5** | **Secure** | Common; widespread and abundant. |

**Table S21:** NatureServe and corresponding IUCN categories and weighting

| **NatureServe Threat Category** | **IUCN Threat Category** | **Weight** |
| --- | --- | --- |
| 5 Secure | LC Least Concern | 1 |
| 4 Apparently Secure | NT Near Threatened | 0.8 |
| 3 Vulnerable | VU Vulnerable | 0.6 |
| 2 Imperiled | EN Endangered | 0.4 |
| 1 Critically Imperiled | CR Critically Endangered | 0.2 |
| X Extinct | EX Extinct | 0 |

## NOAA Fish Stock Sustainability Index (FSSI) Scores

*Where used*: Status and trend for artisanal fishing opportunity goal

*Scale:* Updated regional data

*Description*: The FSSI is a sustainability performance measure produced quarterly by NOAA for 230 commercial and recreational fish stocks across the United States (NMFS 2012). Fish stocks are selected for inclusion in the index based on criteria to determine those that are most important to both the commercial and recreational fishing community. There are 54 stocks included under the jurisdiction of the Pacific Fisheries Management Council (see Table S22 for a full list of species included and Table S36 for details on FSSI scores for all stocks). FSSI scores are calculated based on five criteria: “overfished” status is known, **“**overfishing” status is known, “overfishing” is not occurring (for stocks with known “overfishing” status), stock biomass is above the “overfished” level defined for the stock, and stock biomass is at or above 80% of the biomass that produces maximum sustainable yield (B_MSY_; see Table S22a for scoring breakdown). For this study, the average score across species per region was used.

**Table S22**. Species scientific and common name included in FSSI scores

| **Scientific Name** | **Common Name** | **Scientific Name** | **Common Name** |
| --- | --- | --- | --- |
| Anoplopoma fimbria | Sablefish | Sebastes carnatus | Gopher rockfish |
| Citharichthys sordidus | Pacific sanddab | Sebastes chlorostictus | Greenspotted rockfish |
| Coryphaenoides acrolepis | Pacific grenadier | Sebastes crameri | Darkblotched rockfish |
| Engraulis mordax | Northern anchovy- Northern Pacific Coast | Sebastes diploproa | Splitnose rockfish |
| Engraulis mordax | Northern anchovy- Southern Pacific Coast | Sebastes elongatus | Greenstriped rockfish |
| Eopsetta jordani | Petrale sole | Sebastes entomelas | Widow rockfish |
| Gadus macrocephalus | Pacific cod | Sebastes flavidus | Yellowtail rockfish |
| Glyptocephalus zachirus | Rex sole | Sebastes goodei | Chilipepper |
| Hexagrammos decagrammus | Kelp greenling | Sebastes jordani | Shortbelly rockfish |
| Katsuwonus pelamis | Skipjack tuna | Sebastes levis | Cowcod |
| Loligo opalescens | Opalescent inshore squid | Sebastes melanops | Black rockfish |
| Merluccius productus | Pacific hake | Sebastes melanostomas | Blackgill rockfish |
| Microstomus pacificus | Dover sole | Sebastes miniatus | Vermilion rockfish |
| Ophiodon elongatus | Lingcod | Sebastes mystinus | Blue rockfish |
| Platichthys stellatus | Starry flounder | Sebastes paucispinis | Bocaccio |
| Pleuronectes vetulus | English sole | Sebastes pinniger | Canary rockfish |
| Prionace glauca | Blue shark | Sebastes ruberrimus | Yelloweye rockfish |
| Psettichthys melanostictus | Sand sole | Sebastolobus alascanus | Shortspine thornyhead |
| Raja rhina | Longnose skate | Sebastolobus altivelis | Longspine thornyhead |
| Reinhardtius stomias | Arrowtooth flounder | Squalus acanthias | Spiny dogfish |
| Sardinops sagax | Pacific sardine | Tetrapturus audax | Striped marlin |
| Scomber japonicus | Pacific chub mackerel | Thunnus alalunga | Albacore |
| Scorpaena guttata | California scorpionfish | Thunnus albacares | Yellowfin tuna |
| Scorpaenichthys marmoratus | Cabezon | Thunnus obesus | Bigeye tuna |
| Sebastes aleutianus | Rougheye rockfish | Thunnus orientalis | Pacific bluefin tuna |
| Sebastes alutus | Pacific ocean perch | Trachurus symmetricus | Jack mackerel |
| Sebastes auriculatus | Brown Rockfish | Xiphias gladius | Swordfish |

**Table S22a**. NOAA FSSI Scoring criteria and points awarded.

| **FSSI Criteria** | **Points Awarded** |
| --- | --- |
| “Overfished” status is known | 0.5 |
| “Overfishing” status is known | 0.5 |
| Overfishing is not occurring (for stocks with known “overfishing” status) | 1 |
| Stock biomass is above the “overfished” level defined for the stock | 1 |
| Stock biomass is at or above 80% of the biomass that produces maximum sustainable yield (BMSY) | 1 |
| **Total** | **4** |

##

## NOAA MusselWatch Data

*Where used*: Status and trend for clean waters goal

*Scale:* Updated regional data

*Description*: NOAA MusselWatch data come from marine sediment and bivalve tissue samples collected from U.S. coastal regions during 1986-2009 (O’Conner & Lauenstein 2005, 2006). These samples have measured concentrations for major and trace elements and a suite of organic chemical constituents. Our analysis filters these data to include only the bivalve tissue samples from sampling sites in California, Oregon, and Washington. Also, we omitted samples during years 1997, 1999, 2001, 2003, 2005, 2007, and 2009 when the data were incomplete, mainly in Washington. After filtering, our data include 16,054 organic chemical and 4,343 trace element samples from 81 different sites. We established concentration thresholds for tissue samples in 11 contaminant categories based on the NOAA State of the Coast Report that are originally derived from FDA Action Levels (see table S23 below; Arsenic, Cadmium, Chlordane, Chromium, DDT, Dieldrin, Lead, Mercury, Mirex, Nickel, and PCB), then assigned a numerical score to each sample categorically as 0.0 (bad), 0.5 (ok), and 1.0 (good). We aggregated the scores by computing the mean for each contaminant category, grouped by region and year.

**Table S23:** Breakdown of NOAA MusselWatch sites into the U.S. west coast Sub-regions. Site codes are from O’Conner & Lauenstein (2006, Table 3, pp. 268-276).

| **Sub-region** | **MusselWatch site code** |
| --- | --- |
| **CA1** | BBBE EUSB GCGC HMBJ KRFR PALH PCFB PDSC SGSG SRDM |
| **CA2** | ANAI CBAP DRDP FIEL MBES MBML MBSC MBVG PGLP PLSR PPJB PRNS SFDB SFEM SFSM SFYB SLSL SPSM SPSP SSSS TBSR |
| **CA3** | ABWJ AHCM AHLG ANMI CCSB CDRF CPSB DNPT IBNJ LARM LBBW LJLJ MBVB MDSJ MULG MUOS NBWJ NHPB OSBJ PCPC PDPD PLLH PVRP RBMJ SANM SBSB SCBR SCFP SCRF SDCB SDHI SMOH SNIF SPFP TBSM TJRE |
| **OR** | CBCH CBRP CRSJ CRYB TBHP YBOP YHFC YHSS YHYH |
| **WA** | BBSM CBBP CBTP CRNJ EBDH EBFR GHWJ JFCF JFNB PRPR PSCC PSEF PSEH PSHC PSHI PSKP PSMF PSPA PSPT PSSS SIWP SSBI WBNA WIPP |

## Noise and Light Pollution

*Where used*: Pressure for several goals

*Scale:* Updated regional data

*Description*: To estimate the impact of this stressor in the region we used data available from Halpern et al. (2009) for noise/light pollution. This analysis utilized data from the stable lights at night database (<http://www.ngdc.noaa.gov/dmsp/downloadV4composites.html>) and lights coming from offshore oil rigs and coastal land areas that impact ocean pixels.

## Nutrients

*Where used*: Status and trend for clean waters goal, pressure for many goals

*Scale:* Updated regional data

*Description*: To model nutrient concentrations in the coastal marine environment we use the Halpern et al. 2009 nutrient input layer. This is modeled using a plume distribution seeded with county-level average annual fertilizer use and nitrogen input (USGS 1992-2001, as cited by Halpern et al. 2009). We then created an impact layer mask, derived using the union of all habitats and impact layers, to exclude false positives from true zeros scores. We then ran zonal statistics for the 3 nm offshore zones. The Status score is then equal to 1-x, where x is the zonal mean. The pressure measure is calculated as 1 – the status score, or simply x. The trend is calculated as the change in annual fertilizer input over the last 5 years for which there is input data available per county, and then area weighted to achieve a final value per year.

## Opportunity Index (Social Capital)

*Where used*: Pressure and resilience for all goals

*Scale:* Updated regional data

*Description*: The Opportunity Index was created by Opportunity Nation (Opportunity Nation 2012) as an alternative to the limited information commonly used to assess economic strength and security, namely poverty rates and GDP. This index seeks to provide communities with more diverse and informative data to allow for progression towards increased economic mobility and opportunity. The Opportunity Index measures three dimensions of community opportunity to produce a single overall score for 2,400 counties and all 50 U.S states: Economy, Education, and Community (See Table S24 for all included indicators by dimension). Indicators are selected to measure the opportunities that are available in communities based on their established connection to expanding or restricting economic mobility and opportunity. Most scores are available at both the county and state level, however three indicators are only available at the state level, so these scores were applied equally across all counties in each state. Scores were taken for all coastal counties with the score from each sub-region coming from the area weighted average score across all counties.

**Table S24:** Opportunity Index Themes and Indicators

| **DIMENSION** | **THEME** | **INDICATOR** |
| --- | --- | --- |
| **Jobs and Local Economy Dimension** | JOBS | Unemployment Rate (%) |
|  | WAGES | Median Household Income ($) |
|  | POVERTY | Poverty (% of population below poverty line) |
|  | INEQUALITY | 80/20 Ratio (Ratio of household income at the 80th percentile to that at the 20th percentile) |
|  | ASSETS | Banking Institutions (commercial banks, savings institutions, and credit unions per 1,000 residents) |
|  | AFFORDABLE HOUSING | Households Spending Less than 30% of Household Income on Housing (%) |
|  | INTERNET ACCESS | High-Speed Internet (% of households for states; 5-level categories for counties) |
| **Education Dimension** | PRESCHOOL ENROLLMENT | Preschool (% ages 3 and 4 in school) |
|  | ON-TIME HIGH SCHOOL GRADUATION | On-Time High School Graduation (% of freshmen who graduate in four years) |
|  | POST-SECONDARY EDUCATION | Bachelor's Degree or Higher (% of adults 25 and over) |
| **Community Health and Civic Life Dimension** | CIVIC ENGAGEMENT | Group Membership (% of adults 18 and over involved in social, civic, sports, and religious groups) **[STATES ONLY]** |
|  | VOLUNTEERISM | Volunteerism (% of adults ages 18 and older who did volunteer work any time in the previous year) **[STATES ONLY]** |
|  | YOUTH ECONOMIC AND ACADEMIC INCLUSION | Teenagers Not in School and Not Working (% ages 16-19) **[STATES ONLY]** |
|  | SAFETY | Violent Crime (per 100,000 population) or Homicide (per 100,000) for counties where violent crime rates were not available |
|  | ACCESS TO HEALTH CARE | Primary Care Providers (per 100,000 population) |
|  | ACCESS TO HEALTHY FOOD | Grocery Stores and Supermarkets (% of zip codes with at least one ) |

## pH

*Where used*: Pressure for several goals

*Scale:* Global analysis

*Description*: As was done in the global assessment this pressure models the difference in the distribution of the aragonite saturation state of the ocean in pre-industrial (~1870) and modern times (2000-2009). Changes in the aragonite saturation state can be attributed to changes in the concentration of CO_2_ and thus we use the difference between the pre-industrial and modern times as a proxy for ocean acidification due to human influences. These data are modeled at 1-degree resolution from Halpern et al. (2009) and use global-scale data clipped to the U.S. west coast extent.

## Public Land Ownership

*Where used*: Status for artisanal fishing opportunity goal and lasting special places sub-goal

*Scale:* Updated regional data

*Description*: Public Land Ownership data come from the Protected Area Database of the United States (PAD-US v.1.2) geodatabase created by the United States Geological Survey (USGS) National Gap Analysis Program (GAP). This geodatabase provides public land ownership and conservation information for the entire United States. Data include area, land ownership, level of protection, recreational and cultural uses, as well as other protection and ownership metrics. We considered all lands that were designated as publically owned or privately dedicated to conservation to be public lands that were either accessible by the public or managed for the benefit of public resources, except those owned by the U.S. Department of Defense (DOD). Data were filtered to exclude only the DOD based on the Own_Name feature attribute. We then determined the percentage of all areas within a 1 mile inland coastal buffer that were publically owned and managed for each sub-region.

## Sea Surface Temperature (SST) anomalies

*Where used*: Pressure for several goals

*Scale:* Global analysis

*Description*: As was done in the global assessment this pressure is assessed using the number of positive temperature anomalies that exceed the natural range of variation for a given location, i.e. the degree to which a location experiences unnaturally warm temperature. It is not a measure of absolute temperature at a location. These data come from Halpern et al. (2009) and are clipped to the range of the U.S. west coast.

## Shellfish Growing Areas

*Where used*: Status and trend for mariculture sub-goal

*Scale:* Updated regional data

*Description*: Potential shellfish growing areas were calculated as the total bay area in each sub-region where shellfish are currently harvested from mariculture practices. Total km^2^ covered by each bay come from Dumbauld et al. (2009) and sources therein. Only bays with currently existing mariculture were included in this calculation.

## Shellfish Mariculture Yield

*Where used*: Status and trend for mariculture and fisheries sub-goals

*Scale:* Updated regional data

*Description*: Values for the yield of all shellfish species harvested in each region come from data compiled by the Pacific Coast Shellfish Growers Association (PCSGA) for 2005 and 2009. These data are the most comprehensive, inclusive and available data we are aware of for the region, as many other sources do not include free access of full production statistics on a per-farm or per-region basis. Data are given in pounds of shellfish produced per state per year. These data were collected by the PCSGA from the following organizations: Oregon Department of Agriculture, Powell, Seiler and Co, Certified Public Accountants for Willapa, private shellfish companies in California and Washington.

## Socioeconomic Census Data

*Where used*: Status and trend for tourism and recreation

*Scale:* Updated regional data

*Description*: Data on socioeconomic variables within California, Oregon, and Washington come from the official 2010 US Census (U.S. Census Bureau 2000, 2010). Variables determined by Leeworthy et al. (2005) were extracted from the census data to inform logit models to predict the change in participation rates across 19 coastal recreation activities. See Table S25 for full list of socioeconomic variables used in the analysis.

Table S25. Socioeconomic input variables compiled from US Census for 2000 and 2010.

| **CODE** | **Factors** | **CA** | | **OR** | | **WA** | | | | |
| --- | --- | --- | --- | --- | --- | --- | --- | --- | --- | --- |
|  | Year | 2000 | 2010 | 2000 | 2010 | 2000 | | 2010 | | |
|  |  |  |  |  |  |  | |  | | |
|  | Total Pop | 33,871,648 | 37,253,956 | 3,421,399 | 3,831,074 | 5,894,121 | | 6,724,540 | | |
|  | Total Pop (Civilian Non-Institutionalized) |  |  |  | 99.2% |  | | 98.7% | | |
|  | Total Pop (16+) | 77.0% | 78.1% | 79.6% | 80.0% | 78.7% | | 79.2% | | |
|  |  |  |  |  |  |  | |  | | |
|  | **Age** |  |  |  |  |  | |  | | |
| age16_24 | 16-24 years old | 18.5% | 18.9% | 17.4% | 17.7% | 17.6% | | 18.3% | | |
| age25_34 | 25-34 years old | 20.0% | 18.0% | 17.3% | 18.3% | 18.1% | | 18.5% | | |
| age35_44 | 35-44 years old | 21.0% | 17.5% | 19.3% | 17.4% | 21.0% | | 18.0% | | |
| age45_54 | 45-54 years old | 16.6% | 17.7% | 18.6% | 18.8% | 18.2% | | 19.6% | | |
| age55_64 | 55-64 years old | 10.0% | 13.6% | 11.2% | 17.8% | 10.7% | | 16.5% | | |
| age65p | 65+ years old | 13.8% | 14.3% | 16.1% | 10.1% | 14.3% | | 9.1% | | |
|  |  |  |  |  |  |  | |  | | |
| ccounty | Coastal Resident | 71.6% | 68.5% | 17.9% | 17.0% | 69.1% | | 68.6% | | |
|  |  |  |  |  |  |  | |  | | |
| urban | **Urban Resident** | 97.6% | 97.7% | 78.7% | 81.0% | 82.0% | | 84.1% | | |
|  |  |  |  |  |  |  | |  | | |
|  | **Educational Attainment** |  |  |  |  |  | |  | | |
| educ11 | Less than High School | 23.2% | 19.3% | 14.9% | 11.3% | 12.9% | | 10.2% | | |
| educhs | High School | 20.1% | 20.8% | 26.3% | 25.0% | 24.9% | | 23.8% | | |
| educcoll | Some College or College Degree | 47.1% | 48.9% | 50.2% | 53.2% | 52.9% | | 54.9% | | |
| educgrad | Master's, Professional Degree, or Doctorate | 9.5% | 11.0% | 8.7% | 10.5% | 9.3% | | 11.1% | | |
| educoth | Other | 0.0% | 0.0% | 0.0% | 0.0% | 0.0% | | 0.0% | | |
|  |  |  |  |  |  |  | |  | | |
|  | **Household Income** |  |  |  |  |  | |  | | |
| inc25 | $0-$25,000 | 25.5% | 21.6% | 28.5% | 25.8% | 24.7% | | 21.1% | | |
| inc50 | $25,000-$50,000 | 26.6% | 22.3% | 31.6% | 27.3% | 29.7% | | 23.8% | | |
| inc100 | $50,000-$100,000 | 30.7% | 29.8% | 29.9% | 30.4% | 33.0% | | 33.0% | | |
| inc100p | $100,000+ | 17.3% | 26.4% | 10.0% | 16.4% | 12.6% | | 22.1% | | |
| incmiss | Income Missing | 0.0% | 0.0% | 0.0% | 0.0% | 0.0% | | 0.0% | | |
|  |  |  |  |  |  |  | |  | | |
|  | **Race/Ethnicity** |  |  |  |  |  | |  | | |
| white | White, not Hispanic | 46.7% | 40.1% | 83.5% | 78.5% | 78.9% | | 72.5% | | |
| black | Black, not Hispanic | 6.7% | 5.8% | 1.6% | 1.7% | 3.2% | | 3.4% | | |
| native | Native American or Pacific Islander, not Hispanic | 1.3% | 0.8% | 1.6% | 1.4% | | 2.0% | | 1.9% |
| asian | Asian, not Hispanic | 10.9% | 12.8% | 3.0% | 3.6% | | 5.5% | | 7.1% |
| hispanic | Hispanic | 32.4% | 37.6% | 8.0% | 11.7% | | 7.5% | | 11.2% |
|  | Other, 2+, etc. | 2.0% | 2.8% | 2.3% | 3.0% | | 2.9% | | 3.9% |
|  |  |  |  |  |  | |  | |  |
|  | **Sex** |  |  |  |  | |  | |  |
| male | Male | 49.8% | 49.7% | 49.6% | 49.5% | | 49.8% | | 49.8% |
|  |  |  |  |  |  | |  | |  |
|  | **Census Division** |  |  |  |  | |  | |  |
| cendiv1 | New England | 0.0% | 0.0% | 0.0% | 0.0% | | 0.0% | | 0.0% |
| cendiv2 | Middle Atlantic | 0.0% | 0.0% | 0.0% | 0.0% | | 0.0% | | 0.0% |
| cendiv3 | South Atlantic | 0.0% | 0.0% | 0.0% | 0.0% | | 0.0% | | 0.0% |
| cendiv4 | East South Central | 0.0% | 0.0% | 0.0% | 0.0% | | 0.0% | | 0.0% |
| cendiv5 | West South Central | 0.0% | 0.0% | 0.0% | 0.0% | | 0.0% | | 0.0% |
| cendiv6 | East North Central | 0.0% | 0.0% | 0.0% | 0.0% | | 0.0% | | 0.0% |
| cendiv7 | West North Central | 0.0% | 0.0% | 0.0% | 0.0% | | 0.0% | | 0.0% |
| cendiv8 | Mountain | 0.0% | 0.0% | 0.0% | 0.0% | | 0.0% | | 0.0% |
| cendiv9 | Pacific | 100.0% | 100.0% | 100.0% | 100.0% | | 100.0% | | 100.0% |

## State Competitiveness Report

*Where used*: Resilience measure in the livelihoods and economies goal

*Scale:* Updated regional data

*Description*: The State Competitiveness Report is produced by Beacon Hill Institute on an annual basis and aggregates key microeconomic variables into a single index to measure the economic competitiveness of each state. A state is considered by Beacon Hill Institute to be competitive “if it has in place the policies and conditions that ensure and sustain a high level of per capita income and its continued growth”. Sub-indicators used to assess each state include: Government and fiscal policies, Security, Infrastructure, Human resources, Technology, Business incubation, Openness, and Environmental Policy. Index scores from 2011 were used for all state resilience measures. Overall competitiveness scores are based on a simple average of the eight normalized sub-indices (mean=5, SD=1), which is then again normalized to have a mean of 5 and a standard deviation of 1. We then assigned a score to each state, from 0 to 1, where 1 is the maximum possible index score of 7.39 and 0 is the minimum of 2.88. See the 2011 State Competitiveness Report (<http://www.beaconhill.org/Compete11/Compete2011.pdf>) for full methodological detail. This index was selected to be included in this regional assessment because of its comprehensive nature and because it is most often compared to the Global Competitiveness Index at the country region scale, which was used to measure competitiveness in the Ocean Health Index global assessment.

## State level GDP

*Where used*: Status and trend for economies sub-goal

*Scale:* Updated regional data

*Description*: State level GDP values come from the US Bureau of Economic Analysis (2012) and were used as an adjustment factor for revenue values. This adjustment ensures that changes in the economies sub-goal score reflects only changes specific to revenue from marine-related sectors. If those sectors keep pace with growth in state level GDP or sustain losses no greater than state level decreases in GDP, then the score is 1.0, i.e., no net loss occurred. Raw GDP values are reported from the Bureau of Economic Analysis. We then adjusted these dollar estimates for inflation, and all values are given in year 2000 dollars.

## State level average wages

*Where used*: Status and trend for livelihoods sub-goal

*Scale:* Updated regional data

*Description*: State level wages per capita data come from the US Bureau of Economic Analysis (2012) and were used as an adjustment factor for wages values. This adjustment ensures that changes in the livelihoods sub-goal score reflects only changes specific to wages from marine-related sectors, while if they keep pace with growth in state level average wages or sustain losses comparable to state level decreases in average wages, the score is 1, i.e., no-net loss occurred. Raw average wage values are reported from the Bureau of Economic Analysis. We then adjusted these dollar estimates for inflation, and all values are given in year 2000 dollars.

## State level employment

*Where used*: Status and trend for livelihoods sub-goal

*Scale:* Updated regional data

*Description*: State level employment (number of jobs) data come from the US Bureau of Economic Analysis (2012) and were used as an adjustment factor for jobs values. This adjustment ensures that changes in the livelihoods sub-goal score reflects only changes specific to jobs from marine-related sectors, while if jobs keep pace with growth in state level jobs or sustain losses comparable to state level decreases in jobs the score is 1, i.e., no-net loss occurred.

## Supplemental Marine Species

*Where used*: Status and trend species sub-goal of biodiversity; ecological integrity resilience measure for several goals

*Scale:* Updated regional data

*Description*: Supplemental species risk assessments come from Davies and Baum (2012). This assessment showed that fisheries stock assessments can be used to obtain reasonable proxy values for IUCN risk assessments and provided some calculated scores using their approach. Of these estimated threat categories, 23 were for species present in the U.S. west coast region and were added to the IUCN assessments for the biodiversity calculation. See Table S26 for a full list of additional species and estimated threat status category.

**Table S26.** Additional species and corresponding IUCN threat category (see Table S21) as estimated by Davies and Baum 2012.

| **Common Name** | **Scientific Name** | **Estimated IUCN Threat Category** |
| --- | --- | --- |
| Arrowtooth flounder Pacific Coast | Reinhardtius stomias | NT |
| Blackgill rockfish Pacific Coast | Sebastes melanostomus | NT |
| Black rockfish Northern Pacific Coast | Sebastes melanops | NT |
| Blackrock fish Southern Pacific Coast | Sebastes melanops | NT |
| Blue rockfish California | Sebastes mystinus | VU |
| Cabezon Northern California | Scorpaenichthys marmoratus | NT |
| Cabezon Southern California | Scorpaenichthys marmoratus | NT |
| Cowcod Southern California | Sebastes levis | CR |
| Canary rockfish Pacific Coast | Sebastes pinniger | EN |
| Dover sole Pacific Coast | Microstomus pacicus | NT |
| English sole Pacific Coast | Parophrys vetulus | NT |
| Gopher rockfish Southern Pacific Coast | Sebastes carnatus | NT |
| Kelp greenling Oregon Coast | Hexagrammos decagrammus | NT |
| Longspine thornyhead Pacific Coast | Sebastolobus altivelis | NT |
| Pacific hake Pacific Coast | Merluccius productus | VU |
| Pacific ocean perch Pacific Coast | Sebastes alutus | EN |
| Petrale sole Northern Pacific Coast | Eopsetta jordani | NT |
| Petrale sole Southern Pacific Coast | Eopsetta jordani | NT |
| Starry flounder Northern Pacific Coast | Platichthys stellatus | NT |
| Starry flounder Southern Pacific Coast | Platichthys stellatus | NT |
| Yelloweye rockfish Pacific Coast | Sebastes ruberrimus | EN |
| Yellowtail rockfish Northern Pacific Coast | Sebastes avidus | NT |

## Trash

*Where used*: Status and trend for clean waters goal, pressure for many goals

*Scale:* Updated regional data

*Description*: For the trash layer we used beach cleanup data from the Ocean Conservancy (2010), which reports the amount of trash cleaned up in each state on their yearly International Coastal Cleanup day. These data are reported as pounds per mile of coastline for each state (California values were evenly distributed across the 3 sub-regions because we do not know exactly where the trash came from). We rescaled values from 0 to 1, setting the highest density of trash recorded over the entire time period for all coastal states in the U.S. equal to 1.0 (8135 lbs/mile; found in Oregon in 2010). The status score is then calculated as 1 – (*x* / 8135) where *x* is pounds per mile in each year.

Although the number of people collecting trash is also reported per state, we cannot assume any reliable relationship between the amount of trash collected and the number of people (i.e. whether all trash is collected over the same stretch of beach regardless of the number of people or as a linear or other function), and so we did not use this measure of effort when standardizing the data.

Table S27. Beach cleanup data in pounds per mile.

| **State** | **2007** | **2008** | **2009** | **2010** | **2011** |
| --- | --- | --- | --- | --- | --- |
| CA | 1,084 | 1,745 | 1,693 | 1,333 | 2,142 |
| OR | 5,718 | 14 | 1,494 | 8,135 | 134 |
| WA | 141 | 124 | 247 | 288 | 281 |
| **Total** | **6,942** | **1,883** | **3,433** | **9,756** | **2,556** |

## USDA NASS fertilizer data

*Where used*: Trend for clean waters goal

*Scale:* Updated regional data

*Description*: Trend data for the nutrients layer come from USDA NASS fertilizer data in units of “acres applied” reported by county. We use the 3 most recent data points, collected in years 1997, 2002, and 2007. The minimum and maximum acres applied across all counties and years were used to rescale from 0 to 1, respectively. The scores for 1997, 2002, and 2007 are then calculated on a per county basis as 1-x, where x is the rescaled acres applied per total county acreage. The trend per county is then calculated as the trend in scores across the available observations.

## UV

*Where used*: Pressure for several goals

*Scale:* Global analysis

*Description*: As was done in the global assessment this pressure is measured as the number of times between 2000 and 2004, in each 1 degree cell, that the monthly average UV radiation exceeded the climatological mean + 1 standard deviation within the entire dataset (1996-2004). These values were summed across the 12 months to provide a single value, ranging from 0-19. We did not use the metric of change in these anomalous values from the early period to the current period, as was done for SST data, since the entire UV dataset covers only 9 years. See Halpern et al. 2009 for full details on this dataset.

# ALL HABITATS

## Salt marshes

*Where used*: Status and trend for carbon storage, coastal protection, and biodiversity goals

*Scale:* Updated regional data

*Reference condition (C_r_)*: 50% of historical areal extent present roughly in the 1850s

*Description*: The condition of salt marsh habitats (i.e., wetlands) is calculated based on the percent loss of salt marsh habitats from estimates of historical (generally between 1850-1890) extent from a collection of sources that report habitat extent for some areas within each sub-region (see Table S29 for list of sources and associated reference point years), with a goal of maintaining at least 50% of historical extent in each sub region. The total current extent of salt marsh was calculated as the total extent of all coastal wetland categories in 2006 (see below regarding NOAA C-CAP data). Loss of salt marsh habitats in the region has been extensive since urbanization began. At a state-wide scale, loss of wetlands is estimated at 91% for the state of California, and 31% and 38% for Washington and Oregon, respectively (Dahl 1990). For the coastal zone, habitat loss estimates are generally much higher than for inland areas across the country, with the exception of California, which has lost much of its inland wetland habitats to land conversion and farming (California State of the Wetlands Report). Because much of the loss of salt marsh habitats occurred before monitoring efforts began, available land-use change data for recent years do not capture the actual dramatic extent of loss of these habitats. As such, we chose to set an ambitious yet realistic reference point of at least 50% of intact historical habitat extent as a goal.

Land cover change data for the entire west coast of the United States is available from the NOAA Coastal Change Analysis Program (C-CAP) from 1996-2006. Change analyses have been performed on these data from 1996-2002, and from 2002-2006 based on 23 different land cover classification categories. For this analysis, categories considered to be coastal salt marshes included Estuarine Forested Wetland, Estuarine Scrub/Scrub Wetland, and Estuarine Emergent Wetland (see Table S28 for full description of land cover categories used). We used the change in land cover from 2002-2006 to calculate the trend in habitat condition. It is noted that the EPA is currently in the process of surveying the condition of salt marshes nationally based on estimates of ecological integrity and the presence of stressors on salt marshes habitats. However, this report and the associated data are not expected until at least 2013. These data could supplement our current salt marshes extent data in the future to more accurately assess the condition of salt marshes within the region, however it is too early in the assessment process to predict how and if these data will integrate with extent estimates.

Pressures on salt marshes include pollution, intertidal habitat destruction, and invasive species, as well as governance and social capital pressures. On the resilience side, clean water enforcement and regulation measures, protected area coverage, and governance and social capital measures were included. See Tables S31 and S32 for all pressure and resilience measures applied in the habitats sub-goal.

**Table S28.** NOAA C-CAP land cover class and description utilized in the calculation of salt marsh habitat condition

| **NOAA C-CAP Land Cover Class and Code** | **Description** |
| --- | --- |
| **Estuarine Forested Wetland (16)** | Includes tidal wetlands dominated by woody vegetation greater than or equal to 5 meters in height, and all such wetlands that occur in tidal areas in which salinity due to ocean-derived salts is equal to or greater than 0.5 percent. Total vegetation coverage is greater than 20 percent. |
| **Estuarine Scrub / Shrub Wetland (17)** | Includes tidal wetlands dominated by woody vegetation less than 5 meters in height, and all such wetlands that occur in tidal areas in which salinity due to ocean-derived salts is equal to or greater than 0.5 percent. Total vegetation coverage is greater than 20 percent. |
| **Estuarine Emergent Wetland (18)** | Includes all tidal wetlands dominated by erect, rooted, herbaceous hydrophytes (excluding mosses and lichens). Wetlands that occur in tidal areas in which salinity due to ocean-derived salts is equal to or greater than 0.5 percent and that are present for most of the growing season in most years. Total vegetation cover is greater than 80 percent. Perennial plants usually dominate these wetlands. |

**Table S29.** Sources for historical loss of salt marsh habitats

| **Region** | **Reference** | **Reference Years** |
| --- | --- | --- |
| **Washington** | Collins, B.D. and A.J. Sheikh. 2005 | Early 1850s and early 1890s |
| **Oregon** | Good, J.W. 2000 | 1870 |
| **Northern California** | Barnhart et al. 1992 | 1897 |
| **Central California** | U. S. Environmental Protection Agency. 1999. and  Van Dyke, E. and K. Wasson 2005 | 1800, 1870 |
| **Southern California** | California Department of Fish and Game. 2001a | 1850s to mid-1870s |

## Sand Dunes

*Where used*: Status and trend for coastal protection and biodiversity goals

*Scale:* Updated regional data

*Reference condition (C_r_)*: 100% of the areal extent in 1960

*Description*: Sand dune condition was measured as the change in habitat coverage from ~1960 to 2006 within each sub-region using land cover data for the current extent and dune survey maps (Cooper 1958, 1967) for historical extent. There is no spatially explicit long-term time-series for sand dunes on the west coast, but land cover change data for the entire west coast of the United States is available from the NOAA Coastal Change Analysis Program (C-CAP) from 1996-2006. Change analyses have been performed on these data between 1996 and 2002, and 2002 and 2006 based on 23 different land cover classification categories. We use the C-CAP land cover classifications of Barren Land, Scrub/Shrub, and Unconsolidated Shore (Table S30) within a 1 km buffer of historical sand dune areas as a proxy for current sand dune habitat. We used this 1km buffer to account for the shifting and patchy nature of sand dunes habitats, and we generalized the sand dune habitat classified areas within this 1 km strip, buffered them by 100 m, and then computed the coverage areas using convex hulls to better align with the historical coverage data. The resulting current coverage layer was then compared to the coverage derived from digitizing and georeferencing sand dune maps found in Cooper (1958) for Oregon and Washington and Cooper (1967) for California. Such information allowed for the calculation of the percent of sand dune habitats lost per sub-region between 1960 and 2006. With the target of zero loss of sand dunes over the time period, the condition is then calculated as 1 – the percent loss of sand dune habitats from 1960-2006.

The trend in sand dune habitat was calculated as the trend in habitat extent in the C-CAP land cover data from 2002 to 2006. For sand dunes a limited group of pressures were applied, including intertidal habitat destruction as well as governance and social capital measures on the social side. Resilience measures applied to sand dunes included protected area coverage and governance and social capital indicators. See Table S31 and S32 for all pressure and resilience measures applied in the habitats sub-goal.

**Table S30:** NOAA C-CAP land cover classes and descriptions for identifying sand dune habitats.

| **NOAA C-CAP Land Cover Class and Code** | **Description** |
| --- | --- |
| **Barren Land (20)** | Contains areas of bedrock, desert pavement, scarps, talus, slides, volcanic material, glacial debris, sand dunes, strip mines, gravel pits, and other accumulations of earth material. Generally, vegetation accounts for less than 10 percent of total cover. |
| **Unconsolidated Shore (19)** | Includes material such as silt, sand, or gravel that is subject to inundation and redistribution due to the action of water. Substrates lack vegetation except for pioneering plants that become established during brief periods when growing conditions are favorable. |
| **Scrub/Shrub (12)** | Contains areas dominated by shrubs less than 5 meters tall with shrub canopy typically greater than 20 percent of total vegetation. This class includes tree shrubs, young trees in an early successional stage, or trees stunted from environmental conditions. |

## Seagrasses

*Where used*: Status and trend for carbon storage, coastal protection, and biodiversity goals

*Scale:* Updated regional data

*Reference condition (C_r_)*: Zero pressure to coastal areas from nutrient input.

*Description*: Due to the lack of historical observational data for seagrasses along the west coast, the condition of seagrasses is calculated based on nutrient input pressures applied to the coastal marine system. Nutrient input and the associated increases in turbidity have been shown to negatively impact the condition and coverage of seagrass meadows (Burkholder et al. 1992; Duarte 2002; Orth et al. 2006). We therefore used a nutrient input model taken from Halpern et al. 2009 to measure the amount of pressure present in each sub-region within the coastal zone (defined here as the area within the 100 m depth contour) as a proxy for seagrass condition. Trends in seagrasses were calculated as the trend in habitat extent over the most recent years for which extent data are available. Data used for trend calculations spanned from 2 to 5 years depending on availability, and were compiled from individual sources on a bay-to-bay basis, with all trend data being more recent than 2000 (Boyer and Wyllie-Echeverria 2010, CDFG 2001, CDFG 2010, CRM Inc 2010, Mach et al. 2010, SCCWRP 2011, U.S. Navy 2008, WA DNR 2010). Current extent was calculated for each sub-region based on the seagrass compilation for west coast Essential Fish Habitat (EFH) Environmental Impact Statement data, which is a compilation of 17 different seagrass cover data sources. While these sources span a rather long temporal range, it is the most comprehensive seagrass coverage estimate for the west coast and it is expected that variations across these years would overestimate extent in some cases and underestimate in others, likely resulting in a bias-canceling affect.

Pollution, intertidal habitat destruction, invasive species, and sea surface temperature are the ecological and physical pressures likely to impact seagrasses, as well as governance and social capital pressures (see Table S31). For resilience measures, clean water act enforcement and regulation, protected area coverage, climate change regulations, governance and social capital measures were utilized (see Table S32).

## Soft-bottom

*Where used*: Status and trend for biodiversity goal

*Scale:* Updated regional data

*Reference condition (C_r_)*: Zero pressure from bottom trawl fishing

*Description*: As was the case for the global study, we used bottom-trawling pressure on soft-bottom habitats as a proxy for overall habitat condition. Soft-bottom habitat condition was therefore calculated based on the amount of fish caught across the total area by trawlers in all sub-regions, rescaled so that no bottom-trawling equals zero pressure, and 1 is the maximum pressure limit of 110% of the maximum value of fish caught per soft-bottom area throughout the time series. For California, Oregon, and Washington we use the data provided from the USGS compilation database “Pacific Coast Fisheries GIS Resource Database”. For California the California Commercial Fisheries Data (1972-2009) were provided by the California Department of Fish and Game's Marine Fisheries Statistical Unit. For Oregon and Washington, trawl data came from Oregon Department of Fish Wildlife and the Washington Department of Fish and Wildlife respectively.

# Additional Tables

**Table S31:** Pressure measures included per goal and their associated weights. Weights are color-coded for ease of reference. Blank cells indicate that the stressor is not relevant to that goal or sub-goal. The ‘x’ symbols for social pressures indicate where they were used (social pressures were not ranked 1 to 3).

|  |  | **ECOLOGICAL** | | | | | | | | | | | | | | **PHYSICAL** | | | **SOCIAL** | | | |
| --- | --- | --- | --- | --- | --- | --- | --- | --- | --- | --- | --- | --- | --- | --- | --- | --- | --- | --- | --- | --- | --- | --- |
|  |  | **Pollution** | | | | | | **Habitat Destruction** | | | **Spp Pollution** | | **Fishing Pressure** | | | **Climate Change** | | | **Social** | | | |
| **GOAL** | **SUB-GOAL or SUB-COMPONENT** | Chemicals | Human Pathogens | Nutrients | Trash | Noise Pollution | Light Pollution | HD subtidal soft bottom | HD intertidal trampling | HD intertidal Construction | Alien invasive species | Genetic Escapes | Commercial High bycatch | Commercial Low bycatch | Artisanal Low bycatch | SST | pH | UV | Gas Prices | Governance (Grading the States Report) | Social Capital/Community Opportunity Index | State Competitiveness Report |
| **FOOD PROVISION** | **Fishing** | **1** |  | **1** |  |  |  | **2** | **1** | **1** | **1** | **1** | **3** | **1** | **1** | **1** |  |  | **x** | **x** | **x** |  |
|  | **Mariculture** | **2** |  | **3** |  |  |  |  |  |  |  |  |  |  |  |  | **1** |  |  | **x** | **x** |  |
| **CLEAN WATERS** |  | **3** | **3** | **3** | **3** |  |  |  |  |  |  |  |  |  |  |  |  |  |  | **x** | **x** |  |
| **COASTLINE PROTECTION** | **Seagrasses** | **2** |  | **3** |  |  |  |  | **3** | **3** | **1** |  |  |  |  | **2** |  |  |  | **x** | **x** |  |
|  | **Sand Dunes** |  |  |  |  |  |  |  | **3** | **3** |  |  |  |  |  |  |  |  |  | **x** | **x** |  |
|  | **Salt marshes** | **2** |  | **2** |  |  |  |  | **3** | **3** | **1** |  |  |  |  |  |  |  |  | **x** | **x** |  |
| **CARBON STORAGE** | **Seagrasses** | **2** |  | **3** |  |  |  |  | **3** | **3** | **1** |  |  |  |  | **2** |  |  |  | **x** | **x** |  |
|  | **Salt marshes** | **2** |  | **2** |  |  |  |  | **3** | **3** | **1** |  |  |  |  |  |  |  |  | **x** | **x** |  |
| **TOURISM & RECREATION** | **Tourism & Rec** | **3** | **3** | **3** | **3** |  |  |  |  |  |  |  |  |  |  |  |  |  | **x** | **x** | **x** |  |
| **ARTISANAL OPPORUNITY** |  | **1** |  | **1** |  |  |  | **2** | **1** | **1** | **1** |  | **2** | **1** |  |  |  |  | **x** | **x** | **x** |  |
| **SENSE OF PLACE** | **Special Places** | **2** |  | **2** | **3** | **2** |  |  | **3** | **3** | **1** |  |  |  |  |  |  |  |  | **x** | **x** |  |
|  | **Iconic Species** | **3** |  | **1** | **1** | **2** | **1** |  | **3** | **3** | **1** |  | **2** |  |  | **1** | **1** |  |  | **x** | **x** |  |
| **BIODIVERSITY** | **Habitats - Seagrasses** | **2** |  | **3** |  |  |  |  | **3** | **3** | **1** |  |  |  |  | **2** |  |  |  | **x** | **x** |  |
|  | **Habitats - Salt marshes** | **1** |  | **2** |  |  |  |  | **3** | **3** | **1** |  |  |  |  |  |  |  |  | **x** | **x** |  |
|  | **Habitats - Soft bottom** | **2** |  | **2** |  |  |  | **3** |  |  | **1** |  | **3** | **1** | **1** |  |  |  |  | **x** | **x** |  |
|  | **Habitats - Sand Dunes** |  |  |  |  |  |  |  | **3** | **3** |  |  |  |  |  |  |  |  |  | **x** | **x** |  |
|  | **Species** | **2** |  | **3** | **1** | **1** | **1** | **3** | **2** | **2** | **1** | **1** | **3** | **1** | **1** | **1** | **1** | **1** |  | **x** | **x** |  |
| **LIVELIHOODS** | **Living Resources** | **1** | **0** | **2** | **0** | **0** | **0** | **2** | **1** | **1** | **1** | **1** | **3** | **1** | **1** | **1** | **1** | **0** | **x** | **x** | **x** | **x** |
|  | **Tourism and Recreation** | **3** | **3** | **3** | **3** | **1** | **0** | **0** | **0** | **0** | **0** | **0** | **0** | **0** | **0** | **0** | **0** | **0** | **x** | **x** | **x** | **x** |
|  | **Shipping & Transport** | **0** | **0** | **0** | **0** | **0** | **0** | **0** | **0** | **0** | **1** | **0** | **0** | **0** | **0** | **0** | **0** | **0** | **x** | **x** | **x** | **x** |
|  | **Marine Related Construction** | **0** | **0** | **0** | **0** | **0** | **0** | **0** | **0** | **0** | **0** | **0** | **0** | **0** | **0** | **0** | **0** | **0** | **x** | **x** | **x** | **x** |
|  | **Ship & Boat building/repair** | **0** | **0** | **0** | **0** | **0** | **0** | **0** | **0** | **0** | **0** | **0** | **0** | **0** | **0** | **0** | **0** | **0** | **x** | **x** | **x** | **x** |
|  | **Livelhoods Average** | **1** | **1** | **1** | **1** | **0** | **0** | **0** | **0** | **0** | **0** | **0** | **1** | **0** | **0** | **0** | **0** | **0** | **x** | **x** | **x** | **x** |
|  | **Economies Aaverage** | **1** | **1** | **1** | **1** | **0** | **0** | **0** | **0** | **0** | **0** | **0** | **1** | **0** | **0** | **0** | **0** | **0** | **x** | **x** | **x** | **x** |

**Table S32:** Resilience measures included per goal. Blank cells indicate that the measure is not relevant to that goal or sub-goal.

|  | |  | **Ecological Resilience** | | | | | | | **Social Resilience** | | | |
| --- | --- | --- | --- | --- | --- | --- | --- | --- | --- | --- | --- | --- | --- |
|  | |  | **Regulations** | | | | | **Ecological** | |  |  |  |  |
| **GOAL** | **SUB-GOAL** | | **Clean Water Enforcement and Regulation** | **Lenfest Fisheries Report** | **MPA Coverage (coastal)** | **MPA Coverage (EEZ)** | **Climate Change** | **Ecological Integrity (coastal)** | **Ecological Integrity (EEZ)** | **Gas Price Solution Score** | **Governance (Grading the States Report)** | **Social Capital/ (Opportunity Index)** | **State Competitiveness Report** |
| **FOOD PROVISION** | **Fishing** | |  | **X** |  | **X** |  |  | **X** | **X** | **X** | **X** |  |
|  | **Mariculture** | | **X** |  |  |  |  |  |  |  | **X** | **X** |  |
| **CLEAN WATERS** |  | | **X** |  |  |  |  |  |  |  | **X** | **X** |  |
| **COASTAL PROTECTION** | **Seagrasses** | | **X** |  | **X** |  | **X** |  |  |  | **X** | **X** |  |
|  | **Sand dunes** | |  |  | **X** |  |  |  |  |  | **X** | **X** |  |
|  | **Salt marshes** | | **X** |  | **X** |  |  |  |  |  | **X** | **X** |  |
| **CARBON STORAGE** | **Seagrasses** | | **X** |  | **X** |  | **X** |  |  |  | **X** | **X** |  |
|  | **Salt marshes** | | **X** |  | **X** |  |  |  |  |  | **X** | **X** |  |
| **TOURISM & RECREATION** |  | | **X** |  |  |  |  |  |  | **X** | **X** | **X** |  |
| **ARTISANAL OPPORUNITY** |  | |  | **X** | **X** |  |  | **X** |  | **X** | **X** | **X** |  |
| **SENSE OF PLACE** | **Special Places** | | **X** |  |  | **X** |  |  |  |  | **X** | **X** |  |
|  | **Iconic Species** | | **X** | **X** |  | **X** |  |  | **X** |  | **X** | **X** |  |
| **BIODIVERSITY** | **Habitats - Seagrasses** | | **X** |  | **X** |  | **X** |  | **X** |  | **X** | **X** |  |
|  | **Habitats - Salt marshes** | | **X** |  | **X** |  |  |  | **X** |  | **X** | **X** |  |
|  | **Habitats - Soft bottom** | | **X** | **X** | **X** |  |  |  | **X** |  | **X** | **X** |  |
|  | **Habitats - Sand dunes** | |  |  | **X** |  |  |  |  |  | **X** | **X** |  |
|  | **Species** | | **X** | **X** |  | **X** |  |  |  |  | **X** | **X** |  |
| **LIVELIHOODS** | **Livelihoods** | |  |  |  |  |  |  |  | **X** | **X** | **X** | **X** |
|  | **Economies** | |  |  |  |  |  |  |  | **X** | **X** | **X** | **X** |

**Table S33:** Overall Index, goal and sub-goal scores for every reporting unit (region) in the U.S. west coast study area. Goals (two-letter codes) and sub-goals (three-letter codes) are reported separately (see Fig. 1 in accompanying manuscript for letter codes); LE, SP, and BD goals are the average of sub-goal scores; FP scores are the weighted average of subgoal scores (weights provided in Table S5).

**Table S34:** Full Index scores and per-region, per-goal and sub-goal scores for all four dimensions (status, trend, pressures, and resilience). Region codes are: CA1 = Northern California, CA2 = Central California, CA3 = Southern California, OR = Oregon, WA = Washington.

|  |  |  | Dimension | | | |
| --- | --- | --- | --- | --- | --- | --- |
| (Sub)Goal | Region Code | Score | Status | Trend | Pressures | Resilience |
| FIS | CA1 | 79 | 80 | -0.20 | 30 | 60 |
|  | CA2 | 79 | 80 | -0.20 | 27 | 63 |
|  | CA3 | 79 | 80 | -0.20 | 29 | 63 |
|  | OR | 56 | 58 | -0.17 | 38 | 58 |
|  | WA | 64 | 62 | -0.04 | 28 | 61 |
| FP | CA1 | 77 | 79 | -0.19 | 31 | 60 |
|  | CA2 | 78 | 79 | -0.19 | 27 | 63 |
|  | CA3 | 78 | 79 | -0.19 | 29 | 63 |
|  | OR | 56 | 57 | -0.17 | 38 | 58 |
|  | WA | 53 | 52 | -0.06 | 28 | 55 |
| MAR | CA1 | 24 | 23 | -0.01 | 34 | 63 |
|  | CA2 | 24 | 23 | -0.01 | 32 | 67 |
|  | CA3 | 25 | 23 | -0.01 | 30 | 66 |
|  | OR | 5 | 5 | -0.13 | 35 | 54 |
|  | WA | 27 | 27 | -0.09 | 27 | 42 |
| AO | CA1 | 51 | 48 | 0.01 | 28 | 63 |
|  | CA2 | 57 | 53 | 0.01 | 24 | 65 |
|  | CA3 | 55 | 52 | 0.01 | 25 | 65 |
|  | OR | 69 | 66 | 0.01 | 35 | 61 |
|  | WA | 47 | 44 | 0.05 | 25 | 64 |
| CS | CA1 | 46 | 46 | -0.16 | 26 | 55 |
|  | CA2 | 54 | 52 | -0.02 | 22 | 59 |
|  | CA3 | 61 | 59 | -0.04 | 22 | 58 |
|  | OR | 73 | 70 | -0.01 | 25 | 53 |
|  | WA | 69 | 64 | 0.06 | 18 | 54 |
| CP | CA1 | 51 | 50 | -0.12 | 24 | 55 |
|  | CA2 | 55 | 52 | -0.05 | 22 | 59 |
|  | CA3 | 57 | 57 | -0.14 | 22 | 58 |
|  | OR | 71 | 70 | -0.11 | 24 | 53 |
|  | WA | 57 | 55 | -0.07 | 16 | 54 |
| TR | CA1 | 99 | 98 | 0.14 | 41 | 70 |
|  | CA2 | 99 | 98 | 0.14 | 44 | 72 |
|  | CA3 | 99 | 98 | 0.14 | 45 | 72 |
|  | OR | 100 | 100 | 0.65 | 38 | 57 |
|  | WA | 100 | 100 | 0.58 | 37 | 38 |
| LIV | CA1 | 83 | 82 | -0.18 | 25 | 69 |
|  | CA2 | 94 | 91 | -0.13 | 24 | 73 |
|  | CA3 | 93 | 90 | -0.12 | 24 | 72 |
|  | OR | 96 | 92 | 0.02 | 24 | 68 |
|  | WA | 70 | 70 | -0.29 | 18 | 74 |
| LE | CA1 | 74 | 74 | -0.22 | 25 | 69 |
|  | CA2 | 90 | 86 | -0.11 | 24 | 73 |
|  | CA3 | 97 | 94 | -0.11 | 24 | 72 |
|  | OR | 98 | 96 | 0.20 | 24 | 68 |
|  | WA | 63 | 64 | -0.31 | 18 | 74 |
| ECO | CA1 | 65 | 66 | -0.27 | 25 | 69 |
|  | CA2 | 86 | 82 | -0.10 | 24 | 73 |
|  | CA3 | 99 | 98 | -0.09 | 24 | 72 |
|  | OR | 100 | 100 | 0.37 | 24 | 68 |
|  | WA | 56 | 57 | -0.34 | 18 | 74 |
| ICO | CA1 | 57 | 54 | 0.07 | 33 | 56 |
|  | CA2 | 54 | 52 | 0.01 | 31 | 60 |
|  | CA3 | 57 | 53 | 0.04 | 30 | 60 |
|  | OR | 64 | 61 | 0.03 | 32 | 56 |
|  | WA | 63 | 60 | -0.05 | 25 | 63 |
| SP | CA1 | 48 | 45 | 0.04 | 32 | 51 |
|  | CA2 | 48 | 46 | 0.01 | 30 | 55 |
|  | CA3 | 52 | 49 | 0.03 | 30 | 54 |
|  | OR | 44 | 42 | 0.02 | 30 | 49 |
|  | WA | 45 | 43 | -0.03 | 23 | 52 |
| LSP | CA1 | 38 | 37 | 0.01 | 32 | 45 |
|  | CA2 | 42 | 41 | 0.01 | 30 | 50 |
|  | CA3 | 47 | 45 | 0.01 | 30 | 49 |
|  | OR | 25 | 24 | 0.00 | 28 | 42 |
|  | WA | 28 | 27 | 0.00 | 21 | 41 |
| CW | CA1 | 89 | 85 | 0.02 | 35 | 63 |
|  | CA2 | 85 | 80 | 0.02 | 35 | 67 |
|  | CA3 | 85 | 82 | -0.02 | 37 | 66 |
|  | OR | 89 | 85 | 0.04 | 33 | 54 |
|  | WA | 86 | 84 | 0.01 | 28 | 42 |
| HAB | CA1 | 65 | 65 | -0.16 | 28 | 59 |
|  | CA2 | 74 | 71 | -0.07 | 22 | 64 |
|  | CA3 | 73 | 71 | -0.08 | 25 | 63 |
|  | OR | 64 | 64 | -0.09 | 37 | 60 |
|  | WA | 65 | 61 | -0.01 | 24 | 67 |
| BD | CA1 | 66 | 67 | -0.16 | 32 | 54 |
|  | CA2 | 72 | 70 | -0.11 | 27 | 59 |
|  | CA3 | 72 | 71 | -0.11 | 28 | 58 |
|  | OR | 65 | 66 | -0.10 | 40 | 54 |
|  | WA | 69 | 66 | -0.02 | 28 | 58 |
| SPP | CA1 | 66 | 68 | -0.17 | 35 | 50 |
|  | CA2 | 69 | 70 | -0.15 | 32 | 54 |
|  | CA3 | 72 | 72 | -0.13 | 30 | 53 |
|  | OR | 66 | 68 | -0.11 | 43 | 48 |
|  | WA | 73 | 72 | -0.03 | 32 | 50 |

**Table S35**. Equations and variables used for all goals and sub-goals.

(begins next page)

**Table S36**. List of 54 species for which NOAA provides FSSI assessments and the annual FSSI score used for calculation of OHI (from NOAA quarterly reports).

|  |  | **Official FSSI Score per year** | | | | | |
| --- | --- | --- | --- | --- | --- | --- | --- |
| **FMP** | **Stock** | **2011** | **2010** | **2009** | **2008** | **2007** | **2006** |
| USWCF - HMS / PPFWPRE ^§^ | Albacore - North Pacific | 0 | 0 | 0 | 0 | 0 | 0 |
| Pacific Coast Groundfish | Arrowtooth flounder - Pacific Coast | 4 | 4 | 4 | 4 | 4 | 3 |
| Pacific Coast Groundfish | Bank rockfish - California | 1.5 | 3 | 3 | 3 | 3 | 3 |
| USWCF - HMS / PPFWPRE ^§^ | Bigeye tuna - Pacific | 3 | 2 | 2 | 2 | 2 | 2 |
| Pacific Coast Groundfish | Black rockfish - Northern Pacific Coast | 4 | 4 | 4 | 4 | 4 | 4 |
| Pacific Coast Groundfish | Blackgill rockfish - Southern California | 1.5 | 4 | 4 | 4 | 4 | 4 |
| Pacific Coast Groundfish | Blue rockfish - California | 1.5 | 1.5 | 1.5 | 1.5 | 1.5 | 0 |
| USWCF - HMS / PPFWPRE ^§^ | Blue shark - Pacific | 4 | 4 | 4 | 4 | 4 | 4 |
| Pacific Coast Groundfish | Bocaccio - Southern Pacific Coast | 3 | 3 | 3 | 2 | 2 | 2 |
| Pacific Coast Groundfish | Brown rockfish - Pacific Coast | 0 | 0 | 0 | 0 | 0 | 0 |
| Pacific Coast Groundfish | Cabezon - California | 4 | 4 | 4 | 4 | 4 | 4 |
| Pacific Coast Groundfish | California scorpionfish - Southern California | 4 | 4 | 4 | 4 | 2.5 | 2.5 |
| Pacific Coast Groundfish | Canary rockfish - Pacific Coast | 2 | 2 | 2 | 3 | 3 | 2 |
| Pacific Coast Groundfish | Chilipepper - Southern Pacific Coast | 4 | 4 | 4 | 4 | 4 | 4 |
| Pacific Coast Groundfish | Cowcod - Southern California | 2 | 2 | 2 | 2 | 2 | 2 |
| Pacific Coast Groundfish | Darkblotched rockfish - Pacific Coast | 3 | 3 | 3 | 2 | 2 | 2 |
| USWCF - HMS / PPFWPRE ^§^ | Dolphinfish - Pacific | 0 | 0 | 0 | 0 | 0 | 0 |
| Pacific Coast Groundfish | Dover sole - Pacific Coast | 4 | 4 | 4 | 4 | 4 | 4 |
| Pacific Coast Groundfish | English sole - Pacific Coast | 4 | 4 | 4 | 4 | 4 | 4 |
| Pacific Coast Groundfish | Gopher rockfish - Northern California | 2.5 | 4 | 4 | 4 | 2.5 | 2.5 |
| Coastal Pelagic Species | Jack mackerel - Pacific Coast | 1.5 | 1.5 | 1.5 | 1.5 | 1.5 | 1.5 |
| Pacific Coast Groundfish | Kelp greenling - Oregon | 2.5 | 2.5 | 2.5 | 2.5 | 2.5 | 2.5 |
| Pacific Coast Groundfish | Lingcod - Pacific Coast | 4 | 4 | 4 | 4 | 4 | 4 |
| Pacific Coast Groundfish | Longnose skate - Pacific Coast | 4 | 2.5 | 2.5 | 2.5 | 2.5 | 0 |
| Pacific Coast Groundfish | Longspine thornyhead - Pacific Coast | 4 | 4 | 4 | 4 | 4 | 4 |
| Coastal Pelagic Species | Northern anchovy - Northern Pacific Coast | 0 | 0 | 0 | 0 | 0 | 0 |
| Coastal Pelagic Species | Northern anchovy - Southern Pacific Coast | 1.5 | 1.5 | 1.5 | 1.5 | 1.5 | 1.5 |
| Coastal Pelagic Species | Opalescent inshore squid - Pacific Coast | 0 | 0 | 0 | 0 | 0 | 0 |
| USWCF - HMS / PPFWPRE ^§^ | Pacific bluefin tuna - Pacific | 3 | 3 | 0 | 0 | 0 | 0 |
| Coastal Pelagic Species | Pacific chub mackerel - Pacific Coast | 4 | 4 | 4 | 4 | 4 | 4 |
| Pacific Coast Groundfish | Pacific cod - Pacific Coast | 1.5 | 1.5 | 0 | 0 | 0 | 0 |
| Pacific Coast Groundfish | Pacific grenadier - Pacific Coast | 0 | 0 | 0 | 0 | 0 | 0 |
| Pacific Coast Groundfish | Pacific hake - Pacific Coast | 4 | 3 | 3 | 4 | 4 | 3 |
| Pacific Coast Groundfish | Pacific ocean perch - Pacific Coast | 2 | 3 | 3 | 3 | 3 | 2 |
| Pacific Coast Groundfish | Pacific sanddab - Pacific Coast | 0 | 0 | 0 | 0 | 0 | 0 |
| Coastal Pelagic Species | Pacific sardine - Pacific Coast | 4 | 4 | 4 | 4 | 4 | 4 |
| Pacific Coast Groundfish | Petrale sole - Pacific Coast | 3 | 2 | 2 | 4 | 4 | 3 |
| Pacific Coast Groundfish | Rex sole - Pacific Coast | 0 | 0 | 0 | 0 | 0 | 0 |
| Pacific Coast Groundfish | Rougheye rockfish - Pacific Coast | 0 | 0 | 0 | 0 | 0 | 0 |
| Pacific Coast Groundfish | Sablefish - Pacific Coast | 4 | 4 | 4 | 4 | 4 | 4 |
| Pacific Coast Groundfish | Sand sole - Pacific Coast | 0 | 0 | 0 | 0 | 0 | 0 |
| Pacific Coast Groundfish | Shortbelly rockfish - Pacific Coast | 4 | 4 | 4 | 4 | 4 | 3 |
| Pacific Coast Groundfish | Shortspine thornyhead - Pacific Coast | 4 | 4 | 4 | 4 | 4 | 4 |
| USWCF - HMS / PPFWPRE ^§^ | Skipjack tuna - Eastern Tropical Pacific | 4 | 4 | 4 | 4 | 4 | 4 |
| Pacific Coast Groundfish | Spiny dogfish - Pacific Coast | 2.5 | 0 | 0 | 0 | 0 | 0 |
| Pacific Coast Groundfish | Splitnose rockfish - Pacific Coast | 4 | 4 | 4 | 1.5 | 1.5 | 0 |
| Pacific Coast Groundfish | Starry flounder - Pacific Coast | 4 | 4 | 4 | 4 | 2.5 | 2.5 |
| USWCF - HMS / PPFWPRE ^§^ | Striped marlin - Eastern Tropical Pacific | 4 | 4 | 4 | 4 | 4 | 4 |
| USWCF - HMS / PPFWPRE ^§^ | Swordfish - North Pacific | 4 | 4 | 4 | 4 | 4 | 4 |
| Pacific Coast Groundfish | Vermilion rockfish - California | 0 | 0 | 0 | 0 | 0 | 0 |
| Pacific Coast Groundfish | Widow rockfish - Pacific Coast | 4 | 3 | 3 | 3 | 3 | 3 |
| Pacific Coast Groundfish | Yelloweye rockfish - Pacific Coast | 2 | 2 | 2 | 2 | 2 | 2 |
| USWCF - HMS / PPFWPRE ^§^ | Yellowfin tuna - Eastern Tropical Pacific | 4 | 3 | 3 | 3 | 3 | 3 |
| Pacific Coast Groundfish | Yellowtail rockfish - Northern Pacific Coast | 4 | 4 | 4 | 4 | 4 | 4 |
| § U.S. West Coast Fisheries for Highly Migratory Species / Pacific Pelagic Fisheries of the Western Pacific Region Ecosystem | | | | | | | |

**Table S37**. Details for all data layers used in calculations for each of the goals in the Ocean Health Index.

| **Data Layer** | **Brief Description** | **Dimension** | **Start Year** | **End Year** | **Reference** |
| --- | --- | --- | --- | --- | --- |
| Alien Invasive Species | Number of alien species per marine ecoregion | Pressure | 2008 | 2008 | Molnar et al. 2008 |
| Clean Waters Enforcement and Regulation | Enforcement and compliance metrics utilized by the US EPA National Pollutant Discharge Elimination System (NPDES) permit program | Resilience | 2004 | 2010 | US EPA Enforcement & Compliance History Online (ECHO) database |
| Climate Change Resilience | Implementation of Climate Change related initiatives across 4 sectors | Resilience | 2012 | 2012 | Center for Climate and Energy Solutions (2012) |
| Coastal Access Points | Presence of artisanal poison fishing practices | Status | 1998 | 2012 | NOAA Environmental Sensitivity Index (ESI), Oregon Coastal Atlas Coastal Access Inventory, Washington Marine Shoreline Public Access Project |
| Coastal Counties | Counties with marine or bay coastline | All | 2010 | 2010 | NOAA 2000; ESRI 2010 |
| Coastal Land and Ocean Area | Coastal area per county adjacent to the coastline | All | 2000 | 2000 | NOAA 2000 |
| Coastal population | Population within NOAA Coastal Watershed Counties | All | 2011 | 2011 | NOAA State of the Coast Report |
| Coastline and Coastal Zone Area | Land, inland, and offshore coastal zone buffers for each of our sub-regions and the entire region | All |  |  | Halpern et al. 2012 |
| Ecological integrity | Status of species biological diversity | Resilience | 2011 | 2011 | IUCN 2011 |
| EPA Beach Closure data | Percent of beach days where water samples exceed national pathogen standards by region | Status | 2006 | 2010 | US EPA 2011 |
| FDA Action Levels | Action levels for poisonous or deleterious substances in food that represent limits at or above which the FDA will take legal action to remove food products from the market | Status | 2011 | 2011 | NOAA State of the Coast Report 2011 |
| Fisheries Catch Totals | Catch of all species across years and sub-regions | Status, Trend | 1950 | 2011 | NMFS 2012 |
| Fishing Pressure | Catch of all species across years and sub-regions, gear type used | Pressure | 1950, 1972 | 2011, 2009 | NMFS 2012, USGS 2012 |
| Gas Price | Average yearly gas (diesel) price | Status, Pressure, Resilience | 2000 | 2012 | U.S Energy Information Administration 2012 |
| Gas Price Solution Score | Scores state on how well they are diversifying energy options, moving away from oil dependence, and vulnerability to oil price increases | Resilience | 2011 | 2011 | NRDC 2011 |
| Genetic escapes | Mariculture Sustainability Index (MSI): native or introduced indicator | Pressure | 1994 | 2003 | Trujillo 2008 |
| Grading the States Report (Governance) | Quality of management in each state measured by how well states manage employees, financial details and budgets, information and infrastructure | Pressure, Resilience | 2008 | 2008 | Barrett & Greene 2008 |
| Habitat Destruction, Intertidal Construction | Presence of coastal engineered structures such as riprap, seawalls, jetties, and piers | Pressure | 1998 | 2010 | Halpern et al. 2009 |
| Habitat Destruction, Intertidal Trampling | Model to estimate the impact of direct human trampling | Pressure | 2009 | 2009 | Halpern et al. 2009 |
| Habitat Destruction, subtidal soft-bottom trawling | Pounds of all species caught using trawling gears applied across all soft-bottom habitats | Status, Pressure | 1972 | 2009 | USGS 2012 |
| Lenfest Report | State of enforcement and compliance within U.S. Federally managed fisheries | Resilience | 2009 | 2009 | Lenfest Ocean Program 2009 |
| Mariculture Sustainability Index (MSI): mariculture sustainability and mariculture regulations | Mariculture Sustainability Index (MSI): Mariculture regulations include traceability and code of practice indicators. Mariculture sustainability includes fishmeal use, waste treatment, and seed and larvae origin indicators | Resilience, Status | 1994 | 2003 | Trujillo 2008 |
| Marine and Coastal Recreation Participation | Participation rates by marine-related activity | Status, Trend | 2000 | 2010 | Leeworthy et al. 2005 |
| Marine Jobs, Wages, and Revenue | Marine jobs, wages, and revenue data for six distinct ocean economy sectors, which are comprised of 22 different sub-sectors | Status, Trend | 1990 | 2009 | NOEP 2012 |
| Marine Protected Areas | Year of designation and protection status for over 1700 US protected sites | Status, Trend, Resilience | 2012 | 2012 | National Marine Protected Areas Center 2012 |
| Marine species | IUCN threat category and spatial distribution of marine species | Status, Trend, Resilience | 2011 | 2011 | Carpenter et al. 2008, Schipper et al. 2008, Polidoro et al. 2010, Collette et al. 2011, Knapp et al. 2011, Kaschner et al. 2010 |
| NatureServe Species Assessments | NatureServe threat category and spatial distribution of marine species | Status, Trend | 2012 | 2012 | NatureServe 2012 |
| NOAA Fish Stock Sustainability Index (FSSI) Scores | Sustainability performance measure produced quarterly by NOAA for 230 commercial and recreational fish stocks across the United States | Status, Trend | 2008 | 2012 | NMFS 2012 |
| NOAA MusselWatch Data | Organic chemical and major and trace element concentrations in marine sediment and bivalve tissue samples | Status, Trend | 1986 | 2009 | NOAA MusselWatch 2011 |
| Noise and Light Pollution | Stable lights at night database and lights coming from offshore oil rigs and coastal land areas that impact ocean pixels | Pressure | 2003 | 2003 | Halpern et al. 2009 |
| Nutrients | Modeled N input from fertilizer use | Status, Trend, Pressure | 1992 | 2001 | Halpern et al. 2009, USGS 2001 |
| Opportunity Index (Social Capital) | Measure the community potential to expand economic mobility and opportunity through indicators of economy, education, and community | Pressure | 2012 | 2012 | Opportunity Nation 2012 |
| pH (Ocean acidification) | Change in aragonite saturation state (ASS) levels | Pressure | 1870/ 2000 | 2009 | Halpern et al. 2009 |
| Public Land Ownership | Land ownership, level of protection, recreational and cultural uses, as well as other protection and ownership metrics | Status | 2012 | 2012 | Protected Area Database of the United States (PAD-US v.1.2) |
| Salt marshes | Areal extent of salt marsh habitats | Status, Trend | 1996 | 2006 | NOAA Coastal Change Analysis Program (C-CAP) 2006 |
| Sand Dunes | Areal extent of sand dune habitats | Status, Trend | 1996 | 2006 | NOAA Coastal Change Analysis Program (C-CAP) 2006 |
| Seagrasses | Modeled status for change in condition based on nutrient input pressures | Status, Trend | 1992 | 2001 | Halpern et al. 2009, USGS 2001 |
| Sea surface temperature (SST) anomalies | Sea surface temperature anomalies | Pressure | 1985 | 2005 | Halpern et al. 2008 |
| Shellfish Growing Areas | Total bay area in each sub region where shellfish are currently harvested from mariculture practices | Status, Trend | 2009 | 2009 | Dumbauld et al. 2009 |
| Shellfish Mariculture Yield | Pounds of shellfish produced per state per year | Status, Trend | 2005 | 2009 | Pacific Coast Shellfish Growers Association 2009 |
| Socioeconomic Census Data | Data on socioeconomic variables by state | Status, Trend | 2010 | 2010 | U.S. Census Bureau 2000, 2010 |
| Soft-bottom | Soft-bottom subtidal habitat extent, and modeled status for change in condition | Status, Trend | 1972 | 2009 | Halpern et al. 2009, USGS 2012 |
| State Competitiveness Report | Aggregates key microeconomic variables into a single index to measure the economic competitiveness of each state | Resilience | 2011 | 2011 | Beacon Hill Institute 2011 |
| State level GDP | State level GDP values | Status, Trend | 2007 | 2011 | US Bureau of Economic Analysis 2012 |
| State level average wages | State level wages per capita | Status, Trend | 2007 | 2011 | US Bureau of Economic Analysis 2012 |
| State level employment | State level employment (number of jobs) | Status, Trend | 2007 | 2011 | US Bureau of Economic Analysis 2012 |
| Supplemental Marine Species | Supplemental species risk assessments | Status, Trend, Resilience | 2012 | 2012 | Davies and Baum 2012 |
| Trash | Amount of trash cleaned up in each state on their yearly International Coastal Cleanup day | Status, Trend, Pressure | 2007 | 2011 | Ocean Conservancy 2010 |
| USDA NASS fertilizer data | Fertilizer data in units of “acres applied” reported by county | Trend | 1997 | 2007 | USDA NASS |
| UV | Anomalies in intensity of ultraviolet (UV) radiation | Pressure | 1996 | 2004 | Halpern et al. 2008 |

**Table S38**. Data used for each dimension (status, trend, pressures and resilience) for each goal and subgoal.

| **Goal** | **Sub-Goal** | **Status** | **Trend** | **Pressures** | **Resilience** |
| --- | --- | --- | --- | --- | --- |
| **Food Provision** | Fishing | B/BMSY and F/FMSY estimates from stock assessments | Change in Status over time | Chemicals | Lenfest Fisheries Report |
|  |  |  |  | Nutrients | MPA Coverage |
|  |  |  |  | Habitat destruction: subtidal soft bottom | Diversity Index |
|  |  |  |  | Habitat destruction: intertidal trampling | Gas Price Solution Score |
|  |  |  |  | Habitat destruction: intertidal Construction | Governance (Grading the States Report) |
|  |  |  |  | Alien species | Social Capital (Community Index) |
|  |  |  |  | Genetic escapes |  |
|  |  |  |  | Commercial fishing: high bycatch |  |
|  |  |  |  | Commercial fishing: low bycatch |  |
|  |  |  |  | Artisanal fishing: low bycatch |  |
|  |  |  |  | SST |  |
|  |  |  |  | Gas Price Solution Score |  |
|  |  |  |  | Grading the States Report |  |
|  |  |  |  | Community Opportunity Index |  |
|  | Mariculture | Mariculture yield | Change in Status over time | Chemicals | Clean Water Enforcement and Regulation |
|  |  | Degree of sustainability of culture |  | Nutrients | Governance (Grading the States Report) |
|  |  |  |  | pH | Social Capital (Community Index) |
|  |  |  |  | Governance (Grading the States Report) |  |
|  |  |  |  | Social Capital (Community Index) |  |
| **Artisanal Fishing Opportunities** | Artisanal Fishing Opportunities | **Physical Access:** One coastal access points per mile of coastline **Economic Access:** No increase in the ratio of fuel price to median income over a five-year period  **Resource Access:** Perfect sustainability score for all fish stocks | Change in Status over time | Chemicals | Lenfest Fisheries Report |
|  |  |  |  | Nutrients | MPA Coverage |
|  |  |  |  | Habitat destruction: subtidal soft bottom | Diversity Index |
|  |  |  |  | Habitat destruction: intertidal trampling | Gas Price Solution Score |
|  |  |  |  | Habitat destruction: intertidal construction | Governance (Grading the States Report) |
|  |  |  |  | Alien species | Social Capital (Community Index) |
|  |  |  |  | Commercial fishing: high bycatch |  |
|  |  |  |  | Commercial fishing: low bycatch |  |
|  |  |  |  | Gas Price Solution Score |  |
|  |  |  |  | Governance (Grading the States Report) |  |
|  |  |  |  | Social Capital (Community Index) |  |
| **Carbon Storage** | Seagrass | Zero pressure to coastal areas from nutrient input | Change in Status over time | Chemicals | Clean Water Enforcement and Regulation |
|  |  |  |  | Nutrients | MPA Coverage |
|  |  |  |  | Habitat destruction: intertidal trampling | Climate Change |
|  |  |  |  | Habitat destruction: intertidal construction | Governance (Grading the States Report) |
|  |  |  |  | Alien species | Social Capital (Community Index) |
|  |  |  |  | SST |  |
|  |  |  |  | Governance (Grading the States Report) |  |
|  |  |  |  | Social Capital (Community Index) |  |
|  | Salt marsh | Area | Change in Status over time | Chemicals | Clean Water Enforcement and Regulation |
|  |  | Condtion |  | Nutrients | MPA Coverage |
|  |  |  |  | Habitat destruction: intertidal trampling | Governance (Grading the States Report) |
|  |  |  |  | Habitat destruction: intertidal construction | Social Capital (Community Index) |
|  |  |  |  | Alien species |  |
|  |  |  |  | Governance (Grading the States Report) |  |
|  |  |  |  | Social Capital (Community Index) |  |
| **Coastal Protection** | Sand dunes | Area | Change in Status over time | Habitat destruction: intertidal trampling | MPA Coverage |
|  |  | Condition |  | Habitat destruction: intertidal construction | Governance (Grading the States Report) |
|  |  |  |  | Governance (Grading the States Report) | Social Capital (Community Index) |
|  |  |  |  | Social Capital (Community Index) |  |
|  | Seagrass | Zero pressure to coastal areas from nutrient input | Change in Status over time | Chemicals | Clean Water Enforcement and Regulation |
|  |  |  |  | Nutrients | MPA Coverage |
|  |  |  |  | Habitat destruction: intertidal trampling | Climate Change |
|  |  |  |  | Habitat destruction: intertidal construction | Governance (Grading the States Report) |
|  |  |  |  | Alien species | Social Capital (Community Index) |
|  |  |  |  | SST |  |
|  |  |  |  | Governance (Grading the States Report) |  |
|  |  |  |  | Social Capital (Community Index) |  |
|  | Salt marsh | Area | Change in Status over time | Chemicals | Clean Water Enforcement and Regulation |
|  |  | Condition |  | Nutrients | MPA Coverage |
|  |  |  |  | Habitat destruction: intertidal trampling | Governance (Grading the States Report) |
|  |  |  |  | Habitat destruction: intertidal construction | Social Capital (Community Index) |
|  |  |  |  | Alien species |  |
|  |  |  |  | Governance (Grading the States Report) |  |
|  |  |  |  | Social Capital (Community Index) |  |
| **Coastal Livelihoods & Economies** | Livelihoods | Recent change in marine jobs across sectors | Trend in Livelihoods Status | Chemicals | Gas Price Solution Score |
|  |  | Recent change in marine wages across sectors |  | Human Pathogens | Grading the States Report |
|  |  |  |  | Nutrients | Governance (Grading the States Report) |
|  |  |  |  | Trash | Social Capital (Community Index) |
|  |  |  |  | Noise pollution |  |
|  |  |  |  | Habitat destruction: subtidal soft bottom |  |
|  |  |  |  | Habitat destruction: intertidal trampling |  |
|  |  |  |  | Habitat destruction: intertidal Construction |  |
|  |  |  |  | Alien species |  |
|  |  |  |  | Genetic escapes |  |
|  |  |  |  | Commercial fishing: high bycatch |  |
|  |  |  |  | Commercial fishing: low bycatch |  |
|  |  |  |  | Artisanal fishing: low bycatch |  |
|  |  |  |  | SST |  |
|  |  |  |  | pH |  |
|  |  |  |  | Gas Price Solution Score |  |
|  |  |  |  | Governance (Grading the States Report) |  |
|  |  |  |  | Social Capital (Community Index) |  |
|  |  |  |  | State Competitiveness Report |  |
|  | Economies | Recent change in marine revenue across sectors | Trend in Economies Status | Chemicals | Gas Price Solution Score |
|  |  |  |  | Human Pathogens | Governance (Grading the States Report) |
|  |  |  |  | Nutrients | Social Capital (Community Index) |
|  |  |  |  | Trash | State Competitiveness Report |
|  |  |  |  | Noise pollution |  |
|  |  |  |  | Habitat destruction: subtidal soft bottom |  |
|  |  |  |  | Habitat destruction: intertidal trampling |  |
|  |  |  |  | Habitat destruction: intertidal Construction |  |
|  |  |  |  | Alien species |  |
|  |  |  |  | Genetic escapes |  |
|  |  |  |  | Commercial fishing: high bycatch |  |
|  |  |  |  | Commercial fishing: low bycatch |  |
|  |  |  |  | Artisanal fishing: low bycatch |  |
|  |  |  |  | SST |  |
|  |  |  |  | pH |  |
|  |  |  |  | Gas Price Solution Score |  |
|  |  |  |  | Governance (Grading the States Report) |  |
|  |  |  |  | Social Capital (Community Index) |  |
|  |  |  |  | State Competitiveness Report |  |
| **Tourism & Recreation** | Tourism & Recreation | Per capita participation rates in 19 marine-related activites | Change in Status over time | Chemicals | Clean Water Enforcement and Regulation |
|  |  |  |  | Human Pathogens | Gas Price Solution Score |
|  |  |  |  | Nutrients | Governance (Grading the States Report) |
|  |  |  |  | Trash | Social Capital (Community Index) |
|  |  |  |  | Gas Price Solution Score |  |
|  |  |  |  | Governance (Grading the States Report) |  |
|  |  |  |  | Social Capital (Community Index) |  |
| **Sense of Place** | Iconic Species | Iconic species list | Change in population status | Chemicals | Clean Water Enforcement and Regulation |
|  |  | Iconic species distribution |  | Nutrients | Lenfest Fisheries Report |
|  |  | % of Iconic species of Least Concern |  | Trash | MPA Coverage |
|  |  |  |  | Noise pollution | Diversity Index |
|  |  |  |  | Light Pollution | Governance (Grading the States Report) |
|  |  |  |  | Habitat destruction: intertidal trampling | Social Capital (Community Index) |
|  |  |  |  | Habitat destruction: intertidal Construction |  |
|  |  |  |  | Alien species |  |
|  |  |  |  | Commercial fishing: high bycatch |  |
|  |  |  |  | SST |  |
|  |  |  |  | pH |  |
|  |  |  |  | Governance (Grading the States Report) |  |
|  |  |  |  | Social Capital (Community Index) |  |
|  | Lasting Special Places | Marine protected areas, coastal | Yearly increase in protected areas | Chemicals | Clean Water Enforcement and Regulation |
|  |  | % coastline protected |  | Nutrients | MPA Coverage |
|  |  |  |  | Trash | Governance (Grading the States Report) |
|  |  |  |  | Noise pollution | Social Capital (Community Index) |
|  |  |  |  | Habitat destruction: intertidal trampling |  |
|  |  |  |  | Habitat destruction: intertidal Construction |  |
|  |  |  |  | Alien species |  |
|  |  |  |  | Governance (Grading the States Report) |  |
|  |  |  |  | Social Capital (Community Index) |  |
| **Clean Waters** | Clean Waters | Nutrient pollution | Nutrients | Chemicals | Clean Water Enforcement and Regulation |
|  |  | Pathogens Pollution | Pathogens | Human Pathogens | Governance (Grading the States Report) |
|  |  | Chemical pollution | Chemicals | Nutrients | Social Capital (Community Index) |
|  |  | Trash pollution | Trash | Trash |  |
|  |  |  |  | Governance (Grading the States Report) |  |
|  |  |  |  | Social Capital (Community Index) |  |
| **Biodiversity** | Species | Species threat categories | Change in population status | Chemicals | Clean Water Enforcement and Regulation |
|  |  | Species range maps |  | Nutrients | Lenfest Fisheries Report |
|  |  |  |  | Trash | MPA Coverage |
|  |  |  |  | Noise pollution | Governance (Grading the States Report) |
|  |  |  |  | Light Pollution | Social Capital (Community Index) |
|  |  |  |  | Habitat destruction: subtidal soft bottom |  |
|  |  |  |  | Habitat destruction: intertidal trampling |  |
|  |  |  |  | Habitat destruction: intertidal Construction |  |
|  |  |  |  | Alien species |  |
|  |  |  |  | Genetic escapes |  |
|  |  |  |  | Commercial fishing: high bycatch |  |
|  |  |  |  | Commercial fishing: low bycatch |  |
|  |  |  |  | Artisanal fishing: low bycatch |  |
|  |  |  |  | SST |  |
|  |  |  |  | pH |  |
|  |  |  |  | UV |  |
|  |  |  |  | Governance (Grading the States Report) |  |
|  |  |  |  | Social Capital (Community Index) |  |
|  | Soft bottom | Area | Change in Status over time | Chemicals | Clean Water Enforcement and Regulation |
|  |  | Condition |  | Nutrients | Lenfest Fisheries Report |
|  |  |  |  | Habitat destruction: subtidal soft bottom | MPA Coverage |
|  |  |  |  | Alien species | Diversity Index |
|  |  |  |  | Commercial fishing: high bycatch | Governance (Grading the States Report) |
|  |  |  |  | Commercial fishing: low bycatch | Social Capital (Community Index) |
|  |  |  |  | Artisanal fishing: low bycatch |  |
|  |  |  |  | Governance (Grading the States Report) |  |
|  |  |  |  | Social Capital (Community Index) |  |
|  | Sand dunes | Area | Change in Status over time | Habitat destruction: intertidal trampling | MPA Coverage |
|  |  | Condition |  | Habitat destruction: intertidal Construction | Governance (Grading the States Report) |
|  |  |  |  | Governance (Grading the States Report) | Social Capital (Community Index) |
|  |  |  |  | Social Capital (Community Index) |  |
|  | Seagrass | Zero pressure to coastal areas from nutrient input | Change in Status over time | Chemicals | Clean Water Enforcement and Regulation |
|  |  |  |  | Nutrients | MPA Coverage |
|  |  |  |  | Habitat destruction: intertidal trampling | Climate Change |
|  |  |  |  | Habitat destruction: intertidal construction | Governance (Grading the States Report) |
|  |  |  |  | Alien species | Social Capital (Community Index) |
|  |  |  |  | SST |  |
|  |  |  |  | Governance (Grading the States Report) |  |
|  |  |  |  | Social Capital (Community Index) |  |
|  | Salt marsh | Area | Change in Status over time | Chemicals | Clean Water Enforcement and Regulation |
|  |  | Condition |  | Nutrients | MPA Coverage |
|  |  |  |  | Habitat destruction: intertidal trampling | Governance (Grading the States Report) |
|  |  |  |  | Habitat destruction: intertidal construction | Social Capital (Community Index) |
|  |  |  |  | Alien species |  |
|  |  |  |  | Governance (Grading the States Report) |  |
|  |  |  |  | Social Capital (Community Index) |  |

# Supplemental Figures

**Figure S1**. Modified Kobe plot showing rescaling scheme for B/Bmsy and F/Fmsy used in the Fisheries sub-goal. Capital letters within the plot refer to the three different rules in equation S1b; lower case letters refer to the 7 different rules in Eq. S1c (ordered sequentially). The region where B and f intersect as well as region d represent a perfect fisheries status score (=1.0). Red lines represent where scores are perfect prior to accounting for a buffer; dashed lines around red lines indicate the width of the buffer, within which fisheries status is also scored 1.0. The dashed line separating region ‘c’ from ‘a’ are all given a score of 0 because fishing mortality is beyond sustainable levels.


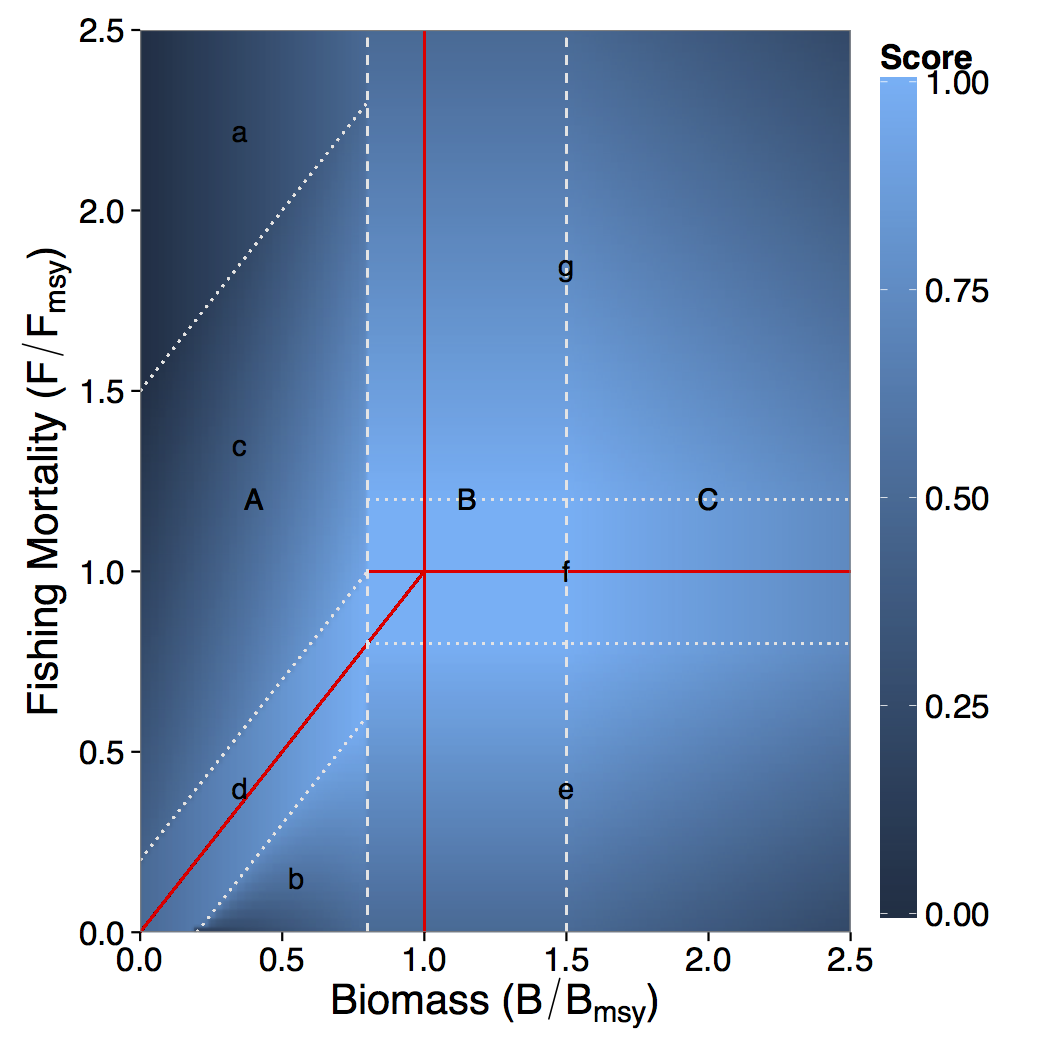


Figure S2: Raster allocation method for lasting special places model for public coastline access with 1 mile resolution. Dark areas represent coastline area with accessibility from access point(s) within 1 mile, and light areas represent coastline area without.

**References**

Arkema, K.K., Guannel, G., Verutes, G., Wood, S.A., Guerry, A., Ruckelshaus, M., Kareiva, Lacayo, P.M., and Silver, J.M. 2013. Coastal habitats shield people and property from sea-level rise and storms. Nature Climate Change, DOI: 10.1038/NCLIMATE1944.

Barnosky, A. D., N. Matzke, S. Tomiya, G. O. U. Wogan, B. Swartz, T. B. Quental, C. Marshall, J. L. McGuire, E. L. Lindsey, K. C. Maguire, B. Mersey, and E. A. Ferrer. 2011. Has the Earth's sixth mass extinction already arrived? Nature **471**:51-57.

Barrett, K., and R. Greene. 2008. Grading the States 2008 Report, The Pew Charitable Trusts’ Government Performance Project

Burkholder, J. M., K. M. Mason, and H. B. Glasgow. 1992. Water-column nitrate enrichment promotes decline of eelgrass Zostera marina – evidence from seasonal mesocosm experiments. Marine Ecology-Progress Series **81**:163-178.

Burns, B, G. Barker, N. Fitzgerald, C. Gillies, J. Innes, J. McLennan, E. Spurr, D. Thornburrow, and C. Watts. 2006. Selection of iconic species for research within IO3 of the Sustaining and Restoring Biodiversity OBI. Landcare Research Contract Report: LC0506/125. Landcare Research New Zealand.

Butchart, S. H. M., H. R. Akcakaya, J. Chanson, J. E. M. Baillie, B. Collen, S. Quader, W. R. Turner, R. Amin, S. N. Stuart, and C. Hilton-Taylor. 2007. Improvements to the Red List Index. Plos One **2**.

California Department of Fish and Game. 2001a. The Status of Habitats and Water Quality in California’s Coastal and Marine Environment. Pp. 29-45; and pp. 481-493 in: **California's Living Marine Resources: A Status Report** (ANR Publication #SG01-11), W.S. Leet, C.M. Dewees, R. Klingbeil and E.J. Larson (eds.)

California Department of Fish and Game. 2001b. Aquaculture Overview. Pp.493-512 in: **California's Living Marine Resources: A Status Report** (ANR Publication #SG01-11), W.S. Leet, C.M. Dewees, R. Klingbeil and E.J. Larson (eds.)

California Department of Fish and Game. 2008. Status of the Fisheries Report 2008.

CAOCEAN (California Ocean and Coastal Environmental Access Network) 2008. available at: <http://projects.atlas.ca.gov/projects/caloceanoceanan/>

Carpenter, K. E., M. Abrar, G. Aeby, R. B. Aronson, S. Banks, A. Bruckner, A. Chiriboga, J. Cortes, J. C. Delbeek, L. DeVantier, G. J. Edgar, A. J. Edwards, D. Fenner, H. M. Guzman, B. W. Hoeksema, G. Hodgson, O. Johan, W. Y. Licuanan, S. R. Livingstone, E. R. Lovell, J. A. Moore, D. O. Obura, D. Ochavillo, B. A. Polidoro, W. F. Precht, M. C. Quibilan, C. Reboton, Z. T. Richards, A. D. Rogers, J. Sanciangco, A. Sheppard, C. Sheppard, J. Smith, S. Stuart, E. Turak, J. E. N. Veron, C. Wallace, E. Weil, and E. Wood. 2008. One-third of reef-building corals face elevated extinction risk from climate change and local impacts. Science **321**:560-563.

Center for Climate and Energy Solutions. 2012. Available at: <http://www.c2es.org/us-states-regions>

Collette, B. B., K. E. Carpenter, B. A. Polidoro, M. J. Juan-Jorda, A. Boustany, D. J. Die, C. Elfes, W. Fox, J. Graves, L. R. Harrison, R. McManus, C. V. Minte-Vera, R. Nelson, V. Restrepo, J. Schratwieser, C. L. Sun, A. Amorim, M. B. Peres, C. Canales, G. Cardenas, S. K. Chang, W. C. Chiang, N. D. Leite, H. Harwell, R. Lessa, F. L. Fredou, H. A. Oxenford, R. Serra, K. T. Shao, R. Sumaila, S. P. Wang, R. Watson, and E. Yanez. 2011. High Value and Long Life-Double Jeopardy for Tunas and Billfishes. Science **333**:291-292.

Collins, B., and Sheikh, A., 2005, Historic reconstruction, classification and change analysis of Puget Sound tidal marshes: Olympia, Wash., Washington Department of Natural Resources, Aquatic Resources Division.

Cooper, W.S. 1958. Coastal Sand Dunes of Oregon and Washington. Geological Society of America, Memoir 72. New York, NY. 169 pp.

Cooper, W.S. 1967. Coastal Dunes of California. Geological Society of America, Memoir 104. Boulder, CO. 131 pp.

Costello, C., D. Ovando, R. Hilborn, S. D. Gaines, and S. E. Lester. 2012. Status and solutions for the world's unassessed fisheries. Science **338**:517-520.

Davies, T. D., and J. K. Baum. 2012. Extinction Risk and Overfishing: Reconciling Conservation and Fisheries Perspectives on the Status of Marine Fishes. Scientific Reports **2**.

Dick, E. J., and A. D. MacCall. 2010. Estimates of Sustainable Yield for 50 Data-poor Stocks in the Pacific Coast Groundfish Fishery Management Plan, NOAA Technical Memorandum NMFS.

Donato, D. C., J. B. Kauffman, D. Murdiyarso, S. Kurnianto, M. Stidham, and M. Kanninen. 2011. Mangroves among the most carbon-rich forests in the tropics. Nature Geoscience **4**:293-297.

Duarte, C. M. 2002. The future of seagrass meadows. Environmental Conservation **29**:192-206.

Dumbauld, B. R., J. L. Ruesink, and S. S. Rumrill. 2009. The ecological role of bivalve shellfish aquaculture in the estuarine environment: A review with application to oyster and clam culture in West Coast (USA) estuaries. Aquaculture **290**:196-223.

ESRI. 2010. Data and Maps for ArcGIS 10. Redlands, CA: ESRI, Inc.

Farr, T. G., and E. al. 2007. The shuttle radar topography mission. Reviews in Geophysics **45**:RG2004.

Good, J.W. 2000. Estuarine Ecosystems. pp. 23-24 in Summary of Current Status and Health of Oregon’s Marine Ecosystems. Statewide Summary. Oregon State of the Environment Report, Oregon Progress Board.

Goodenough, A. E. 2012. Differences in two species-at-risk classification schemes for North American mammals. Journal for Nature Conservation **20**:117-124.

Halpern, B. S., C. V. Kappel, K. A. Selkoe, F. Micheli, C. Ebert, C. Kontgis, C. M. Crain, R. Martone, C. Shearer, and S. J. Teck. 2009. Mapping cumulative human impacts to California Current marine ecosystems Conservation Letters **2**:138-148.

Halpern, B. S., C. Longo, D. Hardy, K. L. McLeod, J. F. Samhouri, S. K. Katona, K. Kleisner, S. E. Lester, J. O'Leary, M. Ranelletti, A. A. Rosenberg, C. Scarborough, E. R. Selig, B. D. Best, D. R. Brumbaugh, F. S. Chapin, L. B. Crowder, K. L. Daly, S. C. Doney, C. Elfes, M. J. Fogarty, S. D. Gaines, K. I. Jacobsen, L. B. Karrer, H. M. Leslie, E. Neeley, D. Pauly, S. Polasky, B. Ris, K. St Martin, G. S. Stone, U. R. Sumaila, and D. Zeller. 2012. An index to assess the health and benefits of the global ocean. Nature **488**:615-620.

Halpern, B. S., C. Longo, K. L. McLeod, R. G. Cooke, B. Fischhoff, J. F. Samhouri, and C. Scarborough. 2013. Elicited preferences for components of ocean health in the California Current. Marine Policy **42**:68-73.

Hughes, T. P., A. H. Baird, D. R. Bellwood, M. Card, S. R. Connolly, C. Folke, R. Grosberg, O. Hoegh-Guldberg, J. B. C. Jackson, J. Kleypas, J. M. Lough, P. Marshall, M. Nystrom, S. R. Palumbi, J. M. Pandolfi, B. Rosen, and J. Roughgarden. 2003. Climate change, human impacts, and the resilience of coral reefs. Science **301**:929-933.

IUCN. 2011.

Lenfest Ocean Program. 2009. An Economic, Legal and Institutional Assessment of Enforcement and Compliance in Federally Managed U.S. Commercial Fisheries.

Keith, D. A., M. A. McCarthy, H. Regan, T. Regan, C. Bowles, C. Drill, C. Craig, B. Pellow, M. A. Burgman, L. L. Master, M. Ruckelshaus, B. Mackenzie, S. J. Andelman, and P. R. Wade. 2004. Protocols for listing threatened species can forecast extinction. Ecology Letters **7**:1101-1108.

Knapp, L., M. M. Mincarone, H. Harwell, B. Polidoro, J. Sanciangco, and K. Carpenter. Conservation status of the world's hagfish species and the loss of phylogenetic diversity and ecosystem function. Aquatic Conservation-Marine and Freshwater Ecosystems **21**:401-411.

McLeod, E., G. L. Chmura, S. Bouillon, R. Salm, M. Bjork, C. M. Duarte, C. E. Lovelock, W. H. Schlesinger, and B. R. Silliman. 2011. A blueprint for blue carbon: toward an improved understanding of the role of vegetated coastal habitats in sequestering CO2. Frontiers in Ecology and the Environment **9**:552-560.

Mehlman, D. W., K. V. Rosenberg, J. V. Wells, and B. Robertson. 2004. A comparison of North American avian conservation priority ranking systems. Biological Conservation **120**:383-390.

Molnar, J. L., R. L. Gamboa, C. Revenga, and M. D. Spalding. 2008. Assessing the global threat of invasive species to marine biodiversity. Frontiers in Ecology and the Environment **6**:485-492.

Nash, C.E. (2004). Achieving policy objectives to increase the value of the seafood industry in the United States: the technical feasibility and associated constraints. Food Policy 29(6):621–641.

National Marine Fisheries Service (NMFS). 2012. Status of the Stocks: Report on the Status of the U.S. Fisheries for 2011.

National Marine Fisheries Service (NMFS). 2012. Annual Commercial Landing Statistics. Available at: <http://www.st.nmfs.noaa.gov/st1/commercial/landings/annual_landings.html>

National Marine Protected Areas Center. 2012. MPA Inventory Database. Available at: <http://www.mpa.gov/dataanalysis/mpainventory/>

National Ocean Economics Program (NOEP). 2012. Ocean Economy Data. Available at: <http://www.oceaneconomics.org/Market/ocean/oceanEcon.asp>

NOAA State of the Coast Report. 2011. Available at: http://stateofthecoast.noaa.gov/musselwatch/welcome.html

NOAA. 2000. Medium Resolution Digital Vector Shoreline. National Oceanic and Atmospheric Administration, National Ocean Service, from http://coastalgeospatial.noaa.gov/shoreline.html

National Oceanic and Atmospheric Administration (NOAA). 2006. NOAA Fisheries Glossary. Revised Edition, June 2006. NOAA Technical Memorandum NMFS-F/SPO-69. Available at: <http://www.st.nmfs.noaa.gov/st4/documents/FishGlossary.pdf>

National Resources Defense Council (NRDC). 2011. Fishint Oil Addiction: Ranking States’ Gasoline Price Vulnerability and Solutions for Change. Report Prepared by David Gardiner & Associates, LLC. Available at: <http://www.nrdc.org/energy/states/>

Natural Capital Project. 2011. Available at <http://invest.ecoinformatics.org/tooldocumentation/>

InVEST_Documentation_v2.1.pdf.

Nellemann, C., Corcoran, E., Duarte, C. M., Valdés, L., De Young, C., Fonseca, L., Grimsditch, G. (Eds). 2009. Blue Carbon. A Rapid Response Assessment. United Nations Environment Programme, GRID-Arendal,

[www.grida.no](http://www.grida.no)

O'Connor, T.P. and G.G. Lauenstein. 2005. Status and trends of copper concentrations in mussels and oysters in the USA. Marine Chemistry, 97(1–2: 49–59)

O'Connor, T.P. and G.G. Lauenstein. 2006. Trends in chemical concentrations in mussels and oysters collected along the US coast: Update to 2003. Marine Environmental Research, 62:261-285.

Opportunity Nation. 2012. Opportunity Index. Available at <http://opportunityindex.org/>

Oregon State of the Environment Report. Summary and Current Status of Oregon’s Estuarine Ecosystems. Chapter III. Health of Natural Systems and Resources. Pp. 33-44; http://www.oregon.gov/dsl/wetland/docs/soer_ch33.pdf

Orth, R. J., T. J. B. Carruthers, W. C. Dennison, C. M. Duarte, J. W. Fourqurean, K. L. Heck, Jr., A. R. Hughes, G. A. Kendrick, W. J. Kenworthy, S. Olyarnik, F. T. Short, M. Waycott, and S. L. Williams. 2006. A global crisis for seagrass ecosystems. Bioscience **56**:987-996.

Polidoro, B. A., K. E. Carpenter, L. Collins, N. C. Duke, A. M. Ellison, J. C. Ellison, E. J. Farnsworth, E. S. Fernando, K. Kathiresan, N. E. Koedam, S. R. Livingstone, T. Miyagi, G. E. Moore, N. N. Vien, J. E. Ong, J. H. Primavera, S. G. Salmo, J. C. Sanciangco, S. Sukardjo, Y. M. Wang, and J. W. H. Yong. 2010. The Loss of Species: Mangrove Extinction Risk and Geographic Areas of Global Concern. Plos One **5**.

Regan, T. J., L. L. Master, and G. A. Hammerson. 2004. Capturing expert knowledge for threatened species assessments: a case study using NatureServe conservation status ranks. Acta Oecologica-International Journal of Ecology **26**:95-107.

Restrepo, V. R., G. G. Thompson, P. M. Mace, W. L. Gabriel, L. L. Low, A. D. MacCall, R. D. Methot, J. E. Powers, B. L. Taylor, P. R. Wade, and W. J. F. 1998. Technical Guidance On the Use of Precautionary Approaches to Implementing National Standard 1 of the Magnuson-Stevens Fishery Conservation and Management Act NOAA Technical Memorandum NMFS-F/SPO-##.

Samhouri, J. F., S. E. Lester, E. R. Selig, B. S. Halpern, M. J. Fogarty, C. Longo, and K. L. McLeod. 2012. Sea sick? Setting targets to assess ocean health and ecosystem services. Ecosphere.

Schipper, J., J. S. Chanson, F. Chiozza, N. A. Cox, M. Hoffmann, V. Katariya, J. Lamoreux, A. S. L. Rodrigues, S. N. Stuart, H. J. Temple, J. Baillie, L. Boitani, T. E. Lacher, Jr., R. A. Mittermeier, A. T. Smith, D. Absolon, J. M. Aguiar, G. Amori, N. Bakkour, R. Baldi, R. J. Berridge, J. Bielby, P. A. Black, J. J. Blanc, T. M. Brooks, J. A. Burton, T. M. Butynski, G. Catullo, R. Chapman, Z. Cokeliss, B. Collen, J. Conroy, J. G. Cooke, G. A. B. da Fonseca, A. E. Derocher, H. T. Dublin, J. W. Duckworth, L. Emmons, R. H. Emslie, M. Festa-Bianchet, M. Foster, S. Foster, D. L. Garshelis, C. Gates, M. Gimenez-Dixon, S. Gonzalez, J. F. Gonzalez-Maya, T. C. Good, G. Hammerson, P. S. Hammond, D. Happold, M. Happold, J. Hare, R. B. Harris, C. E. Hawkins, M. Haywood, L. R. Heaney, S. Hedges, K. M. Helgen, C. Hilton-Taylor, S. A. Hussain, N. Ishii, T. A. Jefferson, R. K. B. Jenkins, C. H. Johnston, M. Keith, J. Kingdon, D. H. Knox, K. M. Kovacs, P. Langhammer, K. Leus, R. Lewison, G. Lichtenstein, L. F. Lowry, Z. Macavoy, G. M. Mace, D. P. Mallon, M. Masi, M. W. McKnight, R. A. Medellin, P. Medici, G. Mills, P. D. Moehlman, S. Molur, A. Mora, K. Nowell, J. F. Oates, W. Olech, W. R. L. Oliver, M. Oprea, B. D. Patterson, W. F. Perrin, B. A. Polidoro, C. Pollock, A. Powel, Y. Protas, P. Racey, J. Ragle, P. Ramani, G. Rathbun, R. R. Reeves, S. B. Reilly, J. E. Reynolds, III, C. Rondinini, R. G. Rosell-Ambal, M. Rulli, A. B. Rylands, S. Savini, C. J. Schank, W. Sechrest, C. Self-Sullivan, A. Shoemaker, C. Sillero-Zubiri, N. De Silva, D. E. Smith, C. Srinivasulu, P. J. Stephenson, N. van Strien, B. K. Talukdar, B. L. Taylor, R. Timmins, D. G. Tirira, M. F. Tognelli, K. Tsytsulina, L. M. Veiga, J.-C. Vie, E. A. Williamson, S. A. Wyatt, Y. Xie, and B. E. Young. 2008. The status of the world's land and marine mammals: Diversity, threat, and knowledge. Science **322**:225-230.

Selig, E.R., C. Longo, **B.S. Halpern**, B.D. Best, D. Hardy, C.T. Elfes, C. Scarborough, K.M. Kleisner and S. Katona. 2013. Assessing global marine biodiversity status within a coupled socio-ecological perspective. PLoS One 8(4): e60284.

Short, F. T., B. Polidoro, S. R. Livingstone, K. E. Carpenter, S. Bandeira, J. S. Bujang, H. P. Calumpong, T. J. B. Carruthers, R. G. Coles, W. C. Dennison, P. L. A. Erftemeijer, M. D. Fortes, A. S. Freeman, T. G. Jagtap, A. H. M. Kamal, G. A. Kendrick, W. J. Kenworthy, Y. A. La Nafie, I. M. Nasution, R. J. Orth, A. Prathep, J. C. Sanciangco, B. van Tussenbroek, S. G. Vergara, M. Waycott, and J. C. Zieman. 2011. Extinction risk assessment of the world's seagrass species. Biological Conservation **144**:1961-1971.

Trujillo, P. 2008. Using a mariculture sustainability index to rank the performance of 53 countries in J. Alder, and D. Pauly, editors. The Performance of 53 Countries in Managing Marine Resources. Fisheries Centre Research Reports Fisheries Centre, University of British Columbia, Vancouver, Canada.

U.S. Bureau of Economic Analysis. 2012. GDP by State. Available at: <http://www.bea.gov/itable/index.cfm>

U.S. Census Bureau. 2010. Census 2010 Summary Files, Demographic Profiles, American Community Survey 1-Year Estimates, and Quick Fact Summaries. Available at: [http://factfinder2.census.gov](http://factfinder2.census.gov/)

U.S. Census Bureau. 2000. Census 2000 Summary Files. Available at: [http://factfinder2.census.gov](http://factfinder2.census.gov/)

U.S. Department of Agriculture (USDA), National Agricultural Statistics Service (NASS). 2005. Census of Aquaculture. USDA 2002 Census of Agriculture.

U.S. Environmental Protection Agency (US EPA). 2011. EPA’s Beach Report Swimming Season Statistics. Available at: <http://water.epa.gov/type/oceb/beaches/beaches_index.cfm>

USGS. 2012. Pacific Coast Fisheries GIS Resource Database CD-ROM. Available upon request.

Van Dyke, E. and K. Wasson. 2005. Historical ecology of a Central California Estuary: 150 years of habitat change. Estuaries 28(2): 173–189.

Wessel, P., and W. H. F. Smith. 1996. A global self-consistent, hierarchical, high-resolution shoreline database. Journal of Geophysical Research **101**:8741-8743.
